# Genetic influences on eight psychiatric disorders based on family data of 4 408 646 full and half-siblings, and genetic data of 333 748 cases and controls

**DOI:** 10.1017/S0033291718002039

**Published:** 2018-09-17

**Authors:** E. Pettersson, P. Lichtenstein, H. Larsson, J. Song, Attention Deficit/Hyperactivity Disorder Working Group of the iPSYCH-Broad-PGC Consortium, Autism Spectrum Disorder Working Group of the iPSYCH-Broad-PGC Consortium, Bipolar Disorder Working Group of the PGC, Eating Disorder Working Group of the PGC, Major Depressive Disorder Working Group of the PGC, Obsessive Compulsive Disorders and Tourette Syndrome Working Group of the PGC, Schizophrenia CLOZUK, Substance Use Disorder Working Group of the PGC, A. Agrawal, A. D. Børglum, C. M. Bulik, M. J. Daly, L. K. Davis, D. Demontis, H. J. Edenberg, J. Grove, J. Gelernter, B. M. Neale, A. F. Pardiñas, E. Stahl, J. T. R. Walters, R. Walters, P. F. Sullivan, D. Posthuma, T. J. C. Polderman

**Affiliations:** 1 Department of Medical Epidemiology and Biostatistics, Karolinska Institutet, Stockholm, Sweden; 2 School of Medical Sciences, Örebro University, Örebro, Sweden; 3 Department of Psychiatry, Washington University in Saint Louis School of Medicine, Saint Louis, MO, USA; 4 Department of Biomedicine, Aarhus University, Aarhus, Denmark; 5 iPSYCH, The Lundbeck Foundation Initiative for Integrative Psychiatric Research, Aarhus, Denmark; 6 iSEQ, Centre for Integrative Sequencing, Aarhus University, Aarhus, Denmark; 7 University of North Carolina at Chapel Hill, Chapel Hill, NC, USA; 8 Analytic and Translational Genetics Unit (ATGU), Department of Medicine, Massachusetts General Hospital and Harvard Medical School, Boston, Massachusetts, USA; 9 Program in Medical and Population Genetics, Broad Institute of Harvard and MIT, Cambridge, Massachusetts, USA; 10 Stanley Center for Psychiatric Research, Broad Institute of Harvard and MIT, Cambridge, Massachusetts, USA; 11 Department of Medicine, Division of Genetic Medicine, Vanderbilt Genetics Institute, Vanderbilt University Medical Center, Nashville, TN, USA; 12 Indiana University School of Medicine, Biochemistry and Molecular Biology, Indianapolis, IN, USA; 13 Indiana University School of Medicine, Medical and Molecular Genetics, Indianapolis, IN, USA; 14 BiRC-Bioinformatics Research Centre, Aarhus University, Aarhus, Denmark; 15 Yale University School of Medicine, Genetics and Neurobiology, New Haven, CT, USA; 16 US Department of Veterans Affairs, Psychiatry, West Haven, CT, USA; 17 Yale University School of Medicine, Psychiatry, New Haven, CT, USA; 18 Medical Research Council Centre for Neuropsychiatric Genetics and Genomics, Cardiff University, Cardiff, Wales; 19 Division of Psychiatric Genomics, Icahn School of Medicine at Mount Sinai, New York, NY, USA; 20 Department of Genetics and Psychiatry, University of North Carolina at Chapel Hill, Chapel Hill, NC, USA; 21 Department of Complex Trait Genetics, Center for Neurogenomics and Cognitive Research (CNCR), Amsterdam Neuroscience, VU University Amsterdam, Amsterdam, The Netherlands; 22 Department of Clinical Genetics, VU University Medical Center (VUMC), Amsterdam, The Netherlands

**Keywords:** ADHD, alcohol dependence, anorexia nervosa, autism spectrum disorders, bipolar disorder, genes, heritability, major depressive disorder, obsessive compulsive disorder, schizophrenia

## Abstract

**Background:**

Most studies underline the contribution of heritable factors for psychiatric disorders. However, heritability estimates depend on the population under study, diagnostic instruments, and study designs that each has its inherent assumptions, strengths, and biases. We aim to test the homogeneity in heritability estimates between two powerful, and state of the art study designs for eight psychiatric disorders.

**Methods:**

We assessed heritability based on data of Swedish siblings (*N* = 4 408 646 full and maternal half-siblings), and based on summary data of eight samples with measured genotypes (*N* = 125 533 cases and 208 215 controls). All data were based on standard diagnostic criteria. Eight psychiatric disorders were studied: (1) alcohol dependence (AD), (2) anorexia nervosa, (3) attention deficit/hyperactivity disorder (ADHD), (4) autism spectrum disorder, (5) bipolar disorder, (6) major depressive disorder, (7) obsessive-compulsive disorder (OCD), and (8) schizophrenia.

**Results:**

Heritability estimates from sibling data varied from 0.30 for Major Depression to 0.80 for ADHD. The estimates based on the measured genotypes were lower, ranging from 0.10 for AD to 0.28 for OCD, but were significant, and correlated positively (0.19) with national sibling-based estimates. When removing OCD from the data the correlation increased to 0.50.

**Conclusions:**

Given the unique character of each study design, the convergent findings for these eight psychiatric conditions suggest that heritability estimates are robust across different methods. The findings also highlight large differences in genetic and environmental influences between psychiatric disorders, providing future directions for etiological psychiatric research.

## Introduction

Psychiatric disorders place an enormous burden on medical resources and society in general (Eaton *et al*., 2008; Petrou *et al*., 2010). For most disorders, the causal factors are as yet largely unknown which limits treatment options considerably. A better understanding of the etiology of psychiatric disorders is a crucial step towards advancing treatment and intervention strategies.

Twin studies showed that genetic factors play an important role in the etiology of psychiatric traits. Heritability estimates (h^2^, i.e. the inherited contribution of genetic variance to trait variance) range from 35% for major depression to over 60% for schizophrenia (SCZ) (Polderman *et al*., 2015). The remaining variance is explained by non-genetic factors perhaps including non-identifiable environmental factors. Another method to derive estimates of genetic and environmental variance is the use of pedigree data (e.g. parents and children, siblings and half-siblings) from large national registers (Pettersson *et al*., 2016). Additionally, rapid methodological developments have recently advanced the use of summary data of genome-wide association studies (GWAS) in which heritability is inferred from the linkage disequilibrium scores (LDSC) of single nucleotide polymorphisms (SNP) (i.e. the ability of a SNP to tag other genetic variants) (Bulik-Sullivan *et al*., 2015). Yet, it is recognized that these heritability estimates do not capture all genetic factors contributing to variance in the trait (such as rare genetic effects), and hence can be viewed as lower bound estimates.

While heritability is conceptualized as a single population parameter, estimates depend on the population under study, ascertainment, diagnostic instruments, and study design. Estimates may also change over time due to variations in diagnostic criteria (Zablotsky *et al*., 2015; Thomas *et al*., 2015), an increase in awareness and detection of psychiatric disorders (Van Naarden Braun *et al*., 2015), changes in the exposure to environmental factors (Rokholm *et al*., 2011), or changes in social situations (Kendler *et al*., 2000).

Using two different methods, this study capitalizes on the largest and most powerful data-sets to date, to estimate the heritability of eight psychiatric conditions: (1) alcohol dependence (AD), (2) anorexia nervosa (AN), (3) attention deficit/hyperactivity disorder (ADHD), (4) autism spectrum disorder (ASD), (5) bipolar disorder (BIP), (6) major depressive disorder (MDD), (7) obsessive-compulsive disorder (OCD), and (8) SCZ. First, we use a large Swedish national cohort (h^2^-national) that currently includes over 20 million full and maternal half-sibling pairs. Unlike most twin studies, the Swedish sibling sample uses clinical diagnoses derived from medical in- and out-patient treatment registers, instead of surveys. Second, we use summary data of eight large samples of subjects with measured SNPs (h^2^-SNP). The uniqueness is estimating heritability from very large samples, based on genetic similarities inferred from distantly related people. As in the h^2^-national design, case status in the h^2^-SNP design is based on diagnostic criteria.

Although each study design has its own strengths, they also have study-specific biases and assumptions (listed in Table 1). For instance, in the national register data that we use, not all affected individuals seek help or are correctly diagnosed. The sibling method also relies on certain assumptions, such as a 100% shared environment despite age differences between siblings, and despite the fact that half-siblings might live in two families (with the biological mother and with the biological father) and thus spend potentially less time together than full siblings (Moffitt *et al*., 2010; Pettersson *et al*., 2016). However, confounding with shared environmental factors is likely excluded in the h^2^-SNP design. Yet, in contrast to sibling studies that assume to capture all possible genetic effects, the h^2^-SNP analysis is based on genome-wide variation that is derived from a selection of *common* genetic variants only (Vinkhuyzen *et al*., 2013). By including a large number of observations from both study designs, our study is adequately powered to robustly estimate in each design the heritability, despite relying on different sets of assumptions and methodologies.

**Table 1.** Strengths, limitations and assumptions of each study design in estimating heritability (h^2^) of psychiatric disorders

Study design	Strengths	Limitations	Assumptions
National sibling design (h^2^–national)	Implicitly includes effects of common and rare genetic effects	No info on actual causal genetic variants	Equal shared sibling environment, also for half siblings
	Psychiatric status based on clinical diagnosis	Needs large samples sizes (tens of thousands of cases) to be able to estimate heritability	Random mating
			Reflection of general population
			No gene × environment interaction
			No gene × environment correlation
SNP-based design (h^2^-SNP)	Based on measured genetic variants	Includes (a priori) tagged common genetic effects only	Random mating
	No confounding with shared environmental factors	Needs large samples sizes (hundreds of thousands of cases and controls) to be able to extract small genetic effects	Reflection of general population
	Psychiatric status based on clinical diagnosis		No gene × environment interaction
			No gene × environment correlation

The aim of this study is to provide a test of the homogeneity in heritability estimates between family-based data (h^2^-national) and SNP-based data for eight psychiatric conditions. Our hypothesis is that h^2^-SNP is lower, but correlates positively with the family-based estimates. In addition, our design can illustrate the differences in etiology between the psychiatric conditions, guiding future directions for etiological research in psychiatry.

## Method

### National sibling cohort (h^2^-national)

Personal identification numbers unique to each individual in Sweden were used to create a national population-based cohort from which twins were excluded. Information was extracted from the National Patient Register, which includes all public psychiatric inpatient diagnoses in Sweden since 1973 and outpatient diagnoses since 2001, assigned by the attending physician with a non-hierarchical diagnostic structure in accord with ICD version 8 (1969–1986), 9 (1987–1996), or 10 (1997–present). The ICD codes for the eight disorders are presented in Table S1 in the Supplementary. We used the Multi-Generation Register to link individuals to their full and maternal half-siblings registered as living in Sweden since 1961 and born in Sweden since 1932. Only two siblings per family were included, starting with the oldest siblings in each family followed by the next oldest sibling, but only if born within 5 years of the first sibling to maximize the probability that they had experienced a similar rearing (i.e. shared) environment. If the two eldest were born more than 5 years apart, we proceeded to the second oldest sibling pair within the family, and so on. The final sample size of full- and maternal half-siblings varied by diagnosis. To ensure that the younger sibling in each pair had lived long enough to receive a potential diagnosis, pairs in which the younger sibling was younger than 5 (for ADHD and ASD), 10 (for AD, AN, MDD, and OCD), or 15 years old (BIP and SCZ) were excluded.

The sibling design assumes (a) that full siblings share an average of 50% of additive genetic effects and 25% of non-additive genetic effects, (b) that maternal half-siblings share an average of 25% of additive genetic effects and 0% of non-additive genetic effects, and (c) that shared environmental effects are 100% shared between both full and maternal half-siblings, and that (d) non-shared environmental effects are unique to each individual. By comparing the observed tetrachoric correlations between the binary diagnoses for full and maternal half-siblings, we estimated the contribution of genetic variance (h^2^-national), and shared and non-shared environmental variance. The analyses were carried out in Mplus (Muthén and Muthén, 1998) using the mean- and variance-adjusted unweighted least squares estimator. We regressed out the effects of sex and age from all diagnoses. For ADHD and ASD, we limited the birth year to 1990 and beyond because these diagnoses only existed in ICD 9 and 10.

### Genetic data (h^2^-SNP)

The h^2^-SNP estimates were based on the most recent available data for all eight disorders in the Psychiatric Genomics Consortium (PGC)(Psychiatric GWAS Consortium Coordinating Committee *et al*., 2009; Psychiatric GWAS Consortium Steering Committee, 2009) (see Study cohort details in the Supplementary). The LDSC approach was used to estimate the h^2^-SNP (Bulik-Sullivan *et al*., 2015). In brief, this method is based on the LDSC of a SNP, which reflects its ability to tag other SNPs. The more SNPs are tagged, the higher the probability that this represents a polygenic signal instead. Therefore, by taking LD into account this method is able to distinguish spurious associations due to population stratification from the true polygenic signal. The LDSC method is also robust to confounding due to shared environmental effects, and is very efficient as it can be applied to GWAS summary statistics (Evans *et al*., 2018). Of note, GWAS usually include millions of common variants, but not rare variants. The heritability (h^2^-SNP), adjusted for the prevalence of the disorder, is inferred from the slope of the regression.

### Analyses

Differences in heritability estimates were tested using *d* = h_1_^2^ − h_2_^2^, 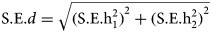. The ratio *Z* = *d*/s.e.(*d*) gives a test of the null hypothesis that the difference *d* is zero, by comparing the value of *Z* to the standard normal distribution(Altman and Bland, 2003). A significance threshold of 0.05 was Bonferroni corrected to accommodate multiple testing (i.e. a correction for eight tests).

## Results

**Fig. 1.**
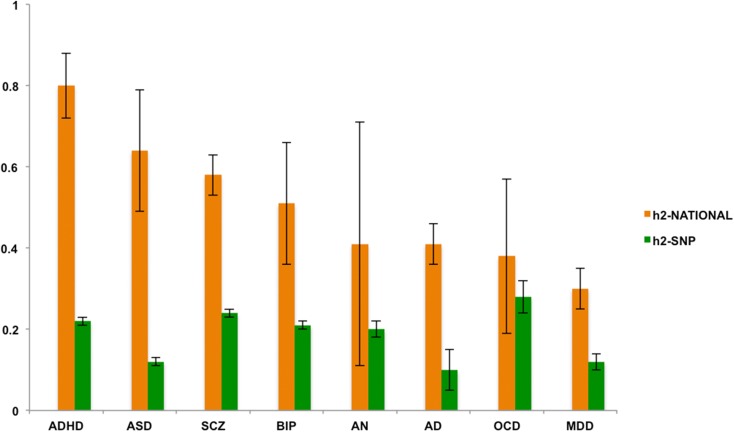
h^2^-national and h^2^-SNP estimates ordered from low to high based on h^2^-national. Note: Error bars represent standard errors. AD, alcohol dependence; ADHD, attention deficit/hyperactivity disorder; AN, anorexia nervosa; ASD, autism spectrum disorder; BIP, bipolar disorder; MDD, major depressive disorder; OCD, obsessive-compulsive disorder; SCZ, schizophrenia.

Estimates of shared environmental effects were non-significant in the sibling analyses. The heritability estimates (Fig. 1) showed significant differences between the h^2^-national estimates and the h^2^-SNP estimates. The latter were significantly lower (corrected *p* < 0.02), except for AN, BIP, and OCD where the h^2^-SNP estimates did not significantly differ from h^2^-national (corrected *p* > 0.30). However, these differences should be interpreted with caution as due to the somewhat smaller samples sizes of these particular disorders, the standard errors (s.e.) of h^2^-national were relatively wide for AN, BIP, and OCD. Of note, the s.e. is in general sensitive to sample size, and in particular for h^2^-national because the full and half-sibling groups only differ by 0.25 in genetic relatedness. Additionally, the nature of summary data of large consortium designs implies that included samples have been genotyped on different platforms and chips, potentially increasing the s.e. of h^2^-SNP.

Heritability estimates from the two designs correlated positively (*r* 0.19). However, this correlation was mainly driven by OCD that showed the highest h^2^-SNP and lowest h^2^-national; when removing OCD this correlation increased to 0.50. The high h^2^-SNP is probably due to the fact that the OCD sample is heavily ascertained from highly multiplex families and early age of onset cases, and consists thus of the most severe and genetically loaded cases.

Tables S2 and S3 in the Supplementary show detailed sample characteristics of each study design.

## Discussion

We estimated the heritability of eight psychiatric conditions using two different study designs: a national sibling design, and a SNP-based design. Estimates derived from the two study designs consistently show that all disorders are moderate to highly heritable but also showed large differences between disorders. The correlation between the family-based and SNP-based estimates was positive (0.50 when leaving out OCD) suggesting that a higher family-based heritability is associated with a larger (aggregated) effect of SNPs. This supports the hypothesis that, apart from rare variants, common genetic variants play an important role in psychiatric disorders, and thus confirms the polygenic nature of these complex traits (Visscher *et al*., 2012).

The heritability estimates based on the large national sibling study (h^2^-national) were remarkably similar to previous twin studies of psychiatric traits (Polderman *et al*., 2015), despite different assessment strategies, with twin studies being survey-based, and as such based on psychiatric trait measures, and the national sibling study based on clinical diagnoses. This might suggest that heritability estimates are robust across different diagnostic tools and measures. It is also in line with studies that reported high genetic correlations between survey-based psychiatric traits and clinical diagnoses, e.g. for ASD (Colvert *et al*., 2015), ADHD (Lubke *et al*., 2009), and psychosis (Zavos *et al*., 2014), suggesting an overlap in genetic factors between psychiatric traits as measured in the general population and clinical disorders.

Heritability estimates based on SNP data (h^2^-SNP) were, as expected, lower than the family-based designs (Yang *et al*., 2017). An obvious explanation for these differences is that h^2^-SNP is based on measured common (and not rare) genotypes, whereas the other design is based on familial relationships and hence includes estimates of genetic factors shared by relatives that are rare in populations. The largest differences between the family-based and h^2^-SNP estimates were observed for the neurodevelopmental traits ADHD and ASD, and for SCZ. Indeed, rare variant risk effects have been reported for ADHD, ASD, and SCZ (Williams *et al*., 2010; Hiroi *et al*., 2013; Sanders *et al*., 2015), although a recent well-powered study on SCZ showed that the explained variance due to rare variants was about 20% of the total explained variance (0.85% for rare variants *v.* 3.4% for common variants) (CNV and Schizophrenia Working Groups of the Psychiatric Genomics Consortium & Psychosis Endophenotypes International Consortium, 2017).

Another explanation for the discrepancy between family-based and SNP-based estimates for ADHD, ASD, and SCZ could be the presence of non-additive effects resulting in overestimates of the narrow-sense heritability in the family-based design, when non-additive influences are removed from the statistical models. Non-additive factors contributing to trait variance have indeed been reported for ADHD (Rietveld *et al*., 2004). Lower h^2^-SNP might also indicate the presence of disorder heterogeneity, that is, a disorder is viewed as a single disorder but actually being a combination of disorder dimensions that has biologically distinct causal factors. As this affects GWAS, and hence h^2^-SNP, most substantially, this explains the lower h^2^-SNP but is also informative about potentially underlying disorder mechanisms (Wray and Maier, 2014). For ADHD and ASD specifically, the inclusion of trio data (i.e. case-pseudo control design) may have underestimated h^2^-SNP due to an increased polygenic burden on the un-transmitted chromosomes (Peyrot *et al*., 2016*a*), although the trio samples were small compared with the much larger case-control samples.

In general, the nature of the large consortium designs on which SNP-based heritability is based likely increases the standard error, all of which will impact on h^2^-SNP. The h^2^-SNP of the eight psychiatric traits as observed in the current study should, therefore, be considered as lower-bound estimates of SNP-heritability. Interestingly, the smallest difference in family-based and SNP-based estimates between both designs was for OCD (respectively, 0.38% *v.* 0.28%). As mentioned previously, the SNP-based estimate of OCD was based on a clinical sample of most severe and therefore probably most genetically loaded cases. Yet, standard errors for both the family-based and SNP-based estimate of OCD were relatively large, so these findings should be interpreted with caution. In a similar vein, one should not stretch the interpretation of the SNP based AD estimate as it derives from one of the smaller genetic samples.

The family-based estimates showed substantial differences in the relative contributions of genes and environment across the eight psychiatric conditions: Heritability estimates for AD, AN, MDD, and OCD were relatively low, ranging from 30 to 41%. However, the prevalence of AN in the family data was low and hence, statistical power was limited, as illustrated by the large standard error in these data. Still, the estimate of 41% for AN in the family data confirms heritability estimates based on twin studies (Polderman *et al*., 2015), also in clinical samples (Mazzeo *et al*., 2009). Heritability estimates of ADHD, ASD, BIP, and SCZ showed the highest narrow-sense heritability estimates between 51 and 80%. With the dramatic increase in sample sizes, the recent endeavors to identify genes that could explain the heritability of psychiatric disorders is becoming more successful. For instance, 108 significantly associated genetic loci were identified for SCZ in a sample of almost 37 000 cases and over 113 000 controls (Schizophrenia Working Group of the Psychiatric Genomics Consortium, 2014). Follow-up analyses on biological pathways revealed that some of the associated genes play an important role in the immune system. However, high heritability not necessarily implies that genetic associations are easy to detect. For example, a recent study in 16 539 ASD cases and over 150 000 controls resulted in only one associated genetic locus (Warrier *et al*., 2017). Similarly, for ADHD only very recently the first 12 associated genetic loci have been published (Demontis *et al*., 2017), illustrating that gene identification in the psychiatric domain is a long and complex avenue.

The fact that heritability estimates show variation across disorders and is never estimated >90% indicates that, discounting potential measurement error, stochasticity, and non-definable environmental factors, definable environmental factors might play an important role in the etiology of psychiatric disorders. In particular, for AD, AN, MDD, and OCD with heritability estimates <50%, environmental etiological research might elucidate crucial pathways that significantly increase the risk for these traits. In addition, gene by environment interaction will likely play a role in the development of psychiatric disorders (Uher and Zwicker, 2017). For instance, one study showed that the effect of a genetic risk for MDD increased in individuals with childhood trauma (Peyrot *et al*., 2014). In other words, given a genetic vulnerability, exposure to certain environmental risk factors will increase the risk for disorder development. However, two recent larger studies (Mullins *et al*., 2016; Peyrot *et al*., 2017) showed no interaction effect. All in all, the empirical evidence for gene by environment interaction in psychiatric disorders is as yet limited (Wray and Maier, 2014) but increasing sample sizes and careful assessments of environmental risk factors are crucial in future research aiming to elucidate causal routes in psychiatric disorders.

## Limitations

Our study has potential limitations. First, the concept of shared environment is not straightforward in the sibling design. Siblings are born at different points in time, do not share the prenatal environment, and are born into different family structures (e.g. the first child born to young parents *v.* the second child born to older parents who already have one child). We aimed to minimize potential time effects by limiting age differences between siblings to a maximum of 5 years. Moreover, additional analyses comparing siblings born within 1–2 *v.* 4–5 years apart showed very similar results (data not shown). Second, in the national sibling design, we assume that the shared environment of full and half siblings is the same. This assumption seems correct: A recent study showed that the vast majority of both Swedish full and maternal half-siblings tend to live together throughout childhood (Pettersson *et al*., 2016). Third, the inclusion age limit for the different disorders in the national sibling design was relatively young (e.g. minimum age of 10 years old for AD, AN, MDD, and OCD) to strike a balance between power on the one hand, and clinical generalizability on the other. However, we cannot rule out that children of that age develop such a disorder later in life. Yet, two additional sets of sensitivity analyses in which only older subjects were included, showed very similar results (online Tables A1 and A2). Fourth, the national sibling cohort lacked information from primary care, which might result in false negatives, in particular, for disorders from the internalizing spectrum and drug abuse (Sundquist *et al*., 2017). However, this source of bias probably has limited influence on the heritability estimates as it is unlikely to differentially impact full- *v.* maternal half-siblings. Nevertheless, failure to include information from primary care decreases power, and limits the generalizability of the study results to the more severe forms of mental health problems that warrant attention by outpatient specialists and inpatient services. Fifth, we compare family-based h^2^ estimates, that were derived from a Swedish sample only, with h^2^-SNP results that were based on a variety of cohorts. Although these are all of the European descent, there might be heterogeneity in h^2^-SNP between cohorts, which makes the comparison with Swedish data less precise. We, therefore, examined heterogeneity in estimates of SCZ, as for this disorder a large Swedish sample contributed to the GWAS from which the h^2^-SNP was derived (Schizophrenia Working Group of the Psychiatric Genomics Consortium, 2014). Supplementary Table S7 of this study clearly shows that the h^2^-SNP based on the Swedish cohort equals h^2^-SNP estimates from other samples of similar size (i.e. Germany, UK), suggesting that the Swedish data are fairly comparable with other data from European descent.

Lastly, both designs assume random mating but a large-scale study in psychiatric populations (Nordsletten *et al*., 2016) observed substantial non-random mating within and across disorders. A recent study, however, concluded that non-random mating has only a very modest effect on SNP-based heritability estimates in psychiatric traits (Peyrot *et al*., 2016*b*).

## Conclusion

In sum, this study presents a converging picture of the etiology of eight psychiatric disorders. SNP-based estimates were as expected lower but correlated with the family-based estimates. Additionally, the findings highlight large differences in genetic and environmental influences between psychiatric disorders. In contrast to ASD, ADHD, BIP, and SCZ, where genetic influences are most important, non-genetic influences play a large role in AD, AN, MDD, and OCD.

## Supplementary material

For supplementary material accompanying this paper visit https://doi.org/10.1017/S0033291718002039. click here to view supplementary material

## Appendices

### Sensitivity analyses

We conducted two sets of sensitivity analyses to examine whether the pre-specified age of the second sibling might have been too young. First, we conducted the analyses in the adult population only by including pairs in which the youngest sibling was 20 years old or older. Results are displayed in Table A1, and were very similar for all disorders except for OCD, for which the heritability estimate decreased from 0.38 to 0.24. However, due to a lack of maternal half-sibling cases, the standard error about this estimate was 0.20, indicating a lack of precision such that the two estimates did not differ significantly.

Second, we plotted the distributions of the age at first diagnosis for each disorder. We then re-ran the analyses but only included pairs where the younger sibling was at least as old as the median age of diagnosis. This way, the youngest siblings were more likely to have lived through the risk period of onset. Results are displayed in Table A2, and show that the estimates are remarkably similar to the original analyses (although the standard errors are larger).

In sum, the heritability estimates remained very similar regardless of whether we relied on our original age cutoffs, or on adults or the median age of onset.

**Table A1.** H^2^ of psychiatric diagnoses based on ICD 8, 9, and 10 in adults of >19 years

	Frequencies								
	Full siblings			Maternal half-siblings			Tetrachoric correlation		
	Cases	Controls	Prevalence (%)	Cases	Controls	Prevalence (%)	Full sibs	Maternal half-sibs	h^2^
Alcohol dependence	87 773	3 043 417	2.80	10 589	157 287	6.31	0.253 (0.004)	0.142 (0.012)	0.444 (0.052)
Anorexia nervosa	8067	3 123 123	0.26	657	167 219	0.39	0.207 (0.020)	0.120 (0.076)	0.349 (0.316)
Attention deficit hyperactivity disorder	5003	209 959	2.33	895	8927	9.11	0.445 (0.014)	0.175 (0.040)	0.852 (0.171)
Autism spectrum disorder	2430	212 532	1.13	237	9585	2.41	0.388 (0.022)	0.055 (0.098)	0.665 (0.402)
Bipolar disorder	18 655	3 112 535	0.60	1619	166 257	0.96	0.303 (0.009)	0.167 (0.038)	0.543 (0.158)
Major depressive disorder	121 015	3 010 175	3.86	12 377	155 499	7.37	0.194 (0.004)	0.117 (0.011)	0.307 (0.048)
Obsessive compulsive disorder	11 553	3 119 637	0.37	1003	166 873	0.60	0.228 (0.014)	0.167 (0.048)	0.244 (0.198)
Schizophrenia	15 178	3 116 012	0.48	1106	166 770	0.66	0.330 (0.010)	0.050 (0.058)	0.569 (0.049)

Note. Standard errors are presented in parentheses.

Sex and age was regressed out from all disorders.

For ADHD and Autism, birth year was limited to 1990 or higher.

Youngest sibling born at age 20 or later.

**Table A2.** H^2^ of psychiatric diagnoses based on ICD 8, 9 , and 10, with the younger sibling being at least as old as the median age of diagnosis

	Frequencies									
	Full siblings			Maternal half-siblings				Tetrachoric correlation		
	Cases	Controls	Prevalence (%)	Cases	Controls	Prevalence (%)	Median age of disorder onset	Full sibs	Maternal half sibs	h^2^
Schizophrenia	14 188	2 335 292	0.60	967	112 193	0.85	34	0.326 (0.010)	0.053 (0.059)	0.564 (0.050)
Bipolar	14 496	2 184 334	0.66	998	103 240	0.96	37	0.297 (0.011)	0.166 (0.048)	0.525 (0.197)
ADHD	10 452	408 760	2.49	2308	21 324	9.77	16	0.455 (0.009)	0.257 (0.024)	0.807 (0.104)
Autism	4900	414 312	1.17	653	22 979	2.76	16	0.410 (0.015)	0.223 (0.049)	0.748 (0.204)
MDD	82 840	2 063 646	3.86	6642	94 294	6.58	38	0.169 (0.004)	0.108 (0.016)	0.242 (0.065)
Anorexia	8706	3 277 732	0.26	700	177 898	0.39	16	0.211 (0.019)	0.115 (0.075)	0.375 (0.309)
OCD	9478	2 751 410	0.34	781	139 567	0.56	26	0.222 (0.016)	0.155 (0.053)	0.267 (0.220)
SUDS & Alcohol	66 035	2 080 451	3.08	6481	94 455	6.42	38	0.265 (0.005)	0 .177 (0.015)	0.351 (0.065)

Note. Standard errors are presented in parentheses.

Sex and age were regressed out from all disorders.

For ADHD and autism, birth year was limited to 1990 or higher.

Youngest sibling being at least as old as disorder median age of onset or later.
