# Supplementary material for: Genetic influences on eight psychiatric disorders based on family data of 4 408 646 full and half-siblings, and genetic data of 333 748 cases and controls
Source: Psychol Med. 2018 Sep 17;49(7):1166–73. doi: 10.1017/S0033291718002039 (PMC6421104; doi:10.1017/S0033291718002039)
Supplement: Supplementary file 1 [file S0033291718002039sup.zip › S0033291718002039sup001.docx]

**SUPPLEMENTARY MATERIAL: Figure and Tables, and study cohort details, authors and affiliations for h^2^-SNP data**

**Genetic influences on eight psychiatric disorders based on family data of 4 408 646 full and half siblings, and genetic data of 333 748 cases and controls**

**By Polderman et al.**

Table of contents Page

**SUPPLEMENTARY TABLES**

Table 1: ICD codes for all eight disorders as used for h^2^-national estimate 2

Table 2: Descriptives national sibling study 3

Table 3: Descriptives SNP-based studies 4

**SUPPLEMENTARY STUDY COHORT DETAILS** h^2^-SNP

Alcohol Dependence 5

Anorexia Nervosa 9

Attention Deficit/Hyperactivity Disorder 11

Autism Spectrum Disorder 12

Bipolar Disorder 14

Major Depressive Disorder 19

Obsessive Compulsive Disorder 20

Schizophrenia 22

References 25

Collaborating Authors and Affiliations 30

**Supplement Table 1. ICD codes for all eight disorders as used for h^2^-national estimates**

| **Definition of psychiatric disorders** | | | |
| --- | --- | --- | --- |
| Psychiatric disorder | ICD 8 (1969-1986) | ICD 9 (1987-1996) | ICD 10 (1997- ) |
| Alcohol Dependence | 291, 2910, 2911, 2912, 2913, 2919, 303, 3030, 3031, 3032, 3039, 5710 | 291, 291A, 291B, 291C, 291D, 291E, 291F, 291W, 291X, 303, 303A, 303X, 357F, 357G, 425F, 535D, 571A, 571B, 571C, 571D, 980, 980A, V11D | F10, F100, F101, F102, F103, F104, F105, F106, F107, F108, G621, G721, I426, K292, K70, K700, K701, K702, K703, K704, K709, K860 |
| Anorexia Nervosa | 3065, 7840 | 307B | F500, F501 |
| ADHD | --- | 314, 314A, 314B, 314C, 314J, 314W, 314X | F90, F900, F901, F908 |
| Autism Spectrum Disorder | --- | 299A | F840, F841, F845 |
| Bipolar Disorder | 2961, 2962, 2963 | 296A, 296C, 296D, 296E, 296F, 296G, 296H, 296W, 296X | F30, F301, F302, F308, F309, F31, F310, F311, F312, F313, F314, F315, F316, F317, F318, F319 |
| Major Depressive Disorder | 2960, 300E | 296B, 311 | F32, F320, F321, F322, F323, F328, F329, F33, F330, F331, F332, F333, F334, F338, F339, F34, F348, F349, F38, F380, F381, F388, F39 |
| Obsessive Compulsive Disorder | 3003 | 300D | F42, F420, F421, F422, F428, F429 |
| Schizophrenia | 295, 2950, 2951, 2952, 2953, 2954, 2956, 2957, 2958, 2959 | 295, 295A, 295B, 295C, 295D, 295E, 295G, 295H, 295W, 295X | F20, F200, F201, F202, F203, F204, F205, F206, F208, F209, F25, F250, F251, F252, F258, F259 |

**Supplement Table 2. Descriptives national sibling study: Per disorder, the number of included full and half siblings, prevalences, tetrachoric sibling correlations and estimated heritability (h^2^), including SE**

|  | **Full siblings** | | **Maternal half siblings** | | | **Tetrachoric correlation (SE)** | | | **h^2^ (SE)** | |  |
| --- | --- | --- | --- | --- | --- | --- | --- | --- | --- | --- | --- |
|  | **N affected** | **N unaffected** | **Prevalence**  **in sample** | **N affected** | **N unaffected** | **Prevalence**  **in sample** | **Full sibs** | **Maternal half sibs** | |  | |
| Alcohol Dependence | 89,526 | 3,567,472 | 2.4% | 11,094 | 187,766 | 5.58% | 0.26 (0.004) | 0.16 (0.012) | | 0.41  (0.05) | |
| Anorexia Nervosa | 9,104 | 3,647,894 | 0.25% | 723 | 198,137 | 0.36% | 0.24 (0.018) | 0.14 (0.074) | | 0.41  (0.30) | |
| ADHD | 18,698 | 1,047,880 | 1.75% | 4,274 | 51,762 | 7.63% | 0.48 (0.007) | 0.28 (0.018) | | 0.80  (0.08) | |
| Autism Spectrum Disorder | 9,347 | 1,057,231 | 0.88% | 1,114 | 54,922 | 1.99% | 0.43 (0.011) | 0.27 (0.036) | | 0.64 (0.15) | |
| Bipolar Disorder | 18,860 | 3,365,174 | 0.56% | 1,676 | 183,040 | 0.91% | 0.31 (0.009) | 0.18 (0.037) | | 0.51  (0.15) | |
| Major Depressive Disorder | 125,627 | 3,531,371 | 3.44% | 13,345 | 185,515 | 6.71% | 0.21 (0.003) | 0.14 (0.011) | | 0.30  (0.05) | |
| Obsessive Compulsive Disorder | 13,044 | 3,643,954 | 0.36% | 1,157 | 197,703 | 0.58% | 0.25 (0.013) | 0.15 (0.046) | | 0.38  (0.19) | |
| Schizophrenia | 15,203 | 3,368,831 | 0.45% | 1,111 | 183,605 | 0.60% | 0.33 (0.010) | 0.05 (0.058) | | 0.58  (0.05) | |

| **Disorder** | **Study** | **N case/N control** | **Prevalence** | **h^2^** |
| --- | --- | --- | --- | --- |
| Alcohol Dependence | PGC AD 1 | 3,772/6,158 | 13,1% | 0.10 (0.05) |
| Anorexia Nervosa | PGC AN 1 | 3,495/10,982 | 0.9% | 0.20 (0.03) |
| ADHD | PGC + iPSYCH ADHD | 19,099/34,194 | 5% | 0.22 (0.01) |
| Autism Spectrum Disorder | PGC + iPSYCH ASD | 18,381/27,969 | 1% | 0.12 (0.01) |
| Bipolar Disorder | PGC BIP 2 | 20,352/31,358 | 1% | 0.21 (0.01) |
| Major Depressive Disorder | PGC MDD 2 | 16,823/25,632 | 10% | 0.12 (0.02) |
| Obsessive Compulsive Disorder | PGC OCD/TS | 2,936/7,279 | 3% | 0.28 (0.04) |
| Schizophrenia | PGC2 + CLOZUK1 | 40,675/64,643 | 1% | 0.24 (0.01) |

**Supplement Table 3. Descriptives SNP-based studies: Samples, prevalences and estimated heritability (h^2^) including SE**

Note: PGC AD1= Substance Use Disorder Working Group of the PGC; PGC AN1= Eating Disorder Working Group of the PGC; PGC + iPSYCH ADHD= Attention Deficit/Hyperactivity Disorder Working Group of the iPSYCH-Broad-PGC Consortium; PGC + iPSYCH ASD= Autism Spectrum Disorder Working Group of the iPSYCH-Broad-PGC Consortium; PGC BIP2= Bipolar Disorder Working Group of the PGC; PGC MDD 2= Major Depressive Disorder Working Group of the PGC; PGC OCD/TS= Obsessive Compulsive Disorders and Tourette Syndrome Working Group of the PGC; CLOZUK= Schizophrenia CLOZUK

**STUDY COHORT DETAILS**

**ALCOHOL DEPENDENCE (AD)**

**SAMPLES**

All samples are part of the PGC Substance Use Disorder (PGC-SUD) Consortium.

Funding: PGC is funded by MH094421. We thank the National Institute of Drug Abuse (NIDA) for supporting us via an administrative supplement and the National Institute of Alcohol Abuse and Alcoholism (NIAAA) for support via the Collaborative Study on the Genetics of Alcoholism (AA008401). AA also acknowledges support from NIDA via DA032573.

**Gene-Environment-Development Initiative -GEDI – Duke University (GSMS)**

**Sample description**: The Duke arm of the NIDA-funded Gene-Environment-Development Initiative (GEDI) combined existing phenotypic and environmental data from two large prospective studies, the Great Smoky Mountains Study (GSMS) and the Caring for Children in the Community (CCC) study. For each of the two population-based contributing studies, genomewide genotyping was conducted using a common platform (Illumina Human660W-Quad v1), generating a total genotyped sample of ~1300 subjects. Further details of the GEDI-Duke sample are available in ^1^ and ^2^.

**Alcohol dependence measure**: Participants of both studies were assessed via structured interviewing using the Young Adult Psychiatric Assessment and its early life extension (i.e., YAPA and CAPA), yielding diagnoses and symptom scales for a wide range of substance use disorders (SUDs). Alcohol dependence was defined using DSM-IV criteria. For the purposes of these analyses, controls were defined as those who had a lifetime history of alcohol drinking but did not meet criteria for alcohol abuse or dependence. No other comorbid diagnoses were excluded.

**Acknowledgements:** This research was supported by the National Institute on Drug Abuse (U01DA024413, R01DA11301), the National Institute of Mental Health (R01MH063970, R01MH063671, R01MH048085, K01MH093731 and K23MH080230), NARSAD, and the William T. Grant Foundation. We are grateful to all the GSMS and CCC study participants who contributed to this work.

**German Study on the Genetics of Alcoholism (GESGA):**

**Sample description:** Patients were recruited from consecutive admissions to the psychiatry and addiction medicine departments of several German psychiatric hospitals participating in the German Addiction Research Network (for detailed description see ^3^ and ^4^). All patients were male and of self-reported German ancestry and fulfilled DSM-IV criteria for AD. Control subjects had been drawn from three population based cohort studies (KORA: <https://www.helmholtz-muenchen.de/kora>; popgen: <https://www.epidemiologie.uni-kiel.de/biobanking/biobank-popgen>; HNR: <https://www.uni-due.de/recall-studie>) in Germany and a Munich community sample. Control samples are mainly population based and can thus comprise alcohol dependent individuals.

**Alcohol dependence measure:** Alcohol dependence was assessed using DSM-IV criteria. Patients received a consensus diagnosis of two clinical psychiatrists and were assessed using one (dependent on recruiting center) of the following (semi-) structured interviews conducted by trained clinical staff members: Semi-Structured Assessment for the Genetics of Alcoholism (SSAGA), Composite International Diagnostic Interview (CIDI) or Structured Clinical Interview for DSM-IV (SCID).

**Acknowledgements:** MR and MMN were supported by the German Federal Ministry of Education and Research (BMBF) through grants BMBF 01ZX1311A (to MR and MMN), and through grants 01ZX1314A (to MMN) and 01ZX1314G (to MR) within the e:Med research program.

**Disclosures:** Dr Wodarz has received funding from the German Research Foundation (DFG) and Federal Ministry of Education and Research Germany (BMBF); he has received speaker’s honoraria and travel funds from Janssen-Cilag and essex pharma. He took part in industry sponsored multi-center randomized trials by D&A pharma and Lundbeck. Monika Ridinger received compensation from Lundbeck Switzerland and Lundbeck institute for advisory boards and expert meeting, and from Lundbeck and Lilly Suisse for workshops and presentations. Karl Mann received honoraria from Lundbeck, Pfizer, Novartis and AbbVie.

**Phenomics and Genomics Sample (PAGES)**

**Sample description:** Individuals in this study were recruited as part of a large schizophrenia case control sample from the Munich greater area and consisted of stable schizophrenia inpatients or outpatients and healthy volunteers. All participants were genetically unrelated, schizophrenia patients were of Caucasian, psychiatrically healthy volunteers of German descent. Candidates with a history of head injury or neurological diseases were excluded.

**Alcohol dependence measure**: Detailed medical and psychiatric histories were collected, including a clinical interview using the Structured Clinical Interview for DSM-IV (SCID), to evaluate lifetime Axis I and II diagnoses. Alcohol dependence was assessed using DSM-IV criteria. Controls were defined as never-having had any alcohol or having a lifetime history of alcohol without meeting the criteria for alcohol abuse or dependence. No other comorbid diagnoses were excluded.

**Acknowledgements**: None

**Christchurch Health and Development study (CHDS)**

**Sample description**: The Christchurch Health and Development study (CHDS)^5^ is a longitudinal study of a birth cohort of 1265 children collected in mid-1977 from urban Christchurch, New Zealand. Data  on social circumstances, health, development and wellbeing of the participants was obtained from the cohort at birth, 4 months, 1 year, annually to age 16 years, and at 18, 21, 25, 30, and 35 years. All study information was collected on the basis of signed consent from study participants and all information is fully confidential. All aspects of the study have been approved by the Canterbury (NZ) Ethics Committee.

**Alcohol dependence measure**: At ages 18, 21, 25, 30 and 35 years cohort members were questioned about their substance use behaviours and problems associated with substance use since the previous assessment (alcohol, tobacco, cannabis, other illicit drugs), using the relevant sections of the Composite International Diagnostic Interview (CIDI) to assess DSM-IV symptom criteria for substance use disorders. Using this information, lifetime alcohol dependence was classified on the basis of whether the participant met DSM criteria for alcohol dependence at any assessment up to age 35.

**Acknowledgements:** The Christchurch Health and Development Study has been supported by funding from the Health Research Council of New Zealand, the National Child Health Research Foundation (Cure Kids), the Canterbury Medical Research Foundation, the New Zealand Lottery Grants Board, the University of Otago, the Carney Centre for Pharmacogenomics, the James Hume Bequest Fund, US NIH grant MH077874, and NIDA grant ‘‘A developmental model of gene-environment interplay in SUDs’’ (R01DA024413) 2007–2012.

**Yale-Penn studies**

**Sample description**: Yale-Penn subjects were recruited in the eastern US, predominantly in Connecticut and Pennsylvania. They were administered the Semi-Structured Assessment for Drug Dependence and Alcoholism (SSADDA)^6^,^7^ to derive DSM-IV diagnoses of lifetime alcohol dependence (and other major psychiatric traits). The study received IRB approval from all participating institutions and written informed consent was obtained from all study participants. Additional information is available in the relevant GWAS publications, e.g.^8^

**Alcohol dependence measure**: DSM-IV diagnoses from the SSADDA.

**Acknowledgements**: Yale-Penn was supported by National Institutes of Health Grants RC2 DA028909, R01 DA12690, R01 DA12849, R01 DA18432, R01 AA11330, and R01 AA017535 and the Veterans Affairs Connecticut and Philadelphia Veterans Affairs Mental Illness Research, Educational, and Clinical Centers.

**Disclosures:** Dr. Kranzler has been an advisory board member, consultant, or continuing medical education speaker for Indivior, Lundbeck, and Otsuka. He is a member of the American Society of Clinical Psychopharmacology’s Alcohol Clinical Trials Initiative, which is sponsored by AbbVie, Alkermes, Ethypharm, Indivior, Lilly, Lundbeck, Pfizer, and Xenoport.

**Collaborative Study on the Genetics of Alcoholism (COGA case control)**

**Sample description**: COGA is a multi-site study of alcohol dependent probands and their family members. Alcohol dependent probands were recruited from inpatient and outpatient facilities. Community probands and their family members were also recruited from a variety of sources. A subset of alcohol dependent cases and genetically unrelated controls were genotyped using the Illumina HumanMap 1M BeadChip. The sample used here included 847 alcohol dependent cases and 552 controls of European-American descent. Additional details are available in^9^.

**Alcohol dependence measure**: All participants were assessed using the Semi-Structured Assessment for the Genetics of Alcoholism^10^. Cases met criteria for a lifetime history of DSM-IV alcohol dependence. Controls reported a history of alcohol drinking, but did not meet criteria for alcohol dependence, abuse or harmful use, nor did they meet criteria for abuse/dependence on illicit drugs.

**Acknowledgements**: We continue to be inspired by our memories of Henri Begleiter and Theodore Reich, founding PI and Co-PI of COGA, and also owe a debt of gratitude to other past organizers of COGA, including Ting-Kai Li, currently a consultant with COGA, P. Michael Conneally, Raymond Crowe, and Wendy Reich, for their critical contributions. This national collaborative study is supported by NIH Grant U10AA008401 from the National Institute on Alcohol Abuse and Alcoholism (NIAAA) and the National Institute on Drug Abuse (NIDA). Funding support for GWAS genotyping, which was performed at the Johns Hopkins University Center for Inherited Disease Research, was provided by the National Institute on Alcohol Abuse and Alcoholism, the NIH GEI (U01HG004438), and the NIH contract "High throughput genotyping for studying the genetic contributions to human disease" (HHSN268200782096C). The authors thank Kim Doheny and Elizabeth Pugh from CIDR and Justin Paschall from the NCBI dbGaP staff for valuable assistance with genotyping and quality control in developing the dataset available at dbGaP.

**Study of Addiction: Genetics and Environment (SAGE) &**

**Collaborative Genetic Study of Nicotine Dependence (COGEND)**

**Sample description:** Subjects were selected from three large, complementary studies: COGA^11^, Family Study of Cocaine Dependence (FSCD^12^), and Collaborative Genetic Study of Nicotine Dependence (COGEND^13^). COGA participants were assessed using the Semi-Structured Assessment for the Genetics of Alcoholism (SSAGA). FSCD and COGEND participants were assessed using polydiagnostic instruments closely based on the SSAGA. Genotyping was conducted using the Illumina Human1Mv1_C BeadChips. Further details of the SAGE sample are available in^14^.

**Alcohol dependence measure**: Cases reported a lifetime history of DSM-IV alcohol dependence. Genetically unrelated control subjects reported alcohol drinking but had no significant alcohol-dependence symptoms and did not meet criteria for a diagnosis of illicit drug dependence.

**Acknowledgements**: Support for the Study of Addiction: Genetics and Environment (SAGE) was provided through the NIH Genes, Environment and Health Initiative [GEI; U01 HG004422; dbGaP study accession phs000092.v1.p1]. SAGE is one of the genome-wide association studies funded as part of the Gene Environment Association Studies (GENEVA) under GEI. Assistance with phenotype harmonization and genotype cleaning, as well as with general study coordination, was provided by the GENEVA Coordinating Center [U01 HG004446]. Assistance with data cleaning was provided by the National Center for Biotechnology Information. Support for collection of datasets and samples was provided by the Collaborative Study on the Genetics of Alcoholism [COGA; U10 AA008401], the Collaborative Genetic Study of Nicotine Dependence [COGEND; P01 CA089392], and the Family Study of Cocaine Dependence [FSCD; R01 DA013423, R01 DA019963]. Funding support for genotyping, which was performed at the Johns Hopkins University Center for Inherited Disease Research (CIDR), was provided by the NIH GEI [U01HG004438], the National Institute on Alcohol Abuse and Alcoholism, the National Institute on Drug Abuse, and the NIH contract "High throughput genotyping for studying the genetic contributions to human disease" [HHSN268200782096C].

**Disclosures**: Laura J. Bierut is listed as an inventor on Issued U.S. Patent 8,080,371,“Markers for Addiction” covering the use of certain SNPs in determining the diagnosis, prognosis, and treatment of addiction.

**Comorbidity and Trauma Study (CATS)**

**Sample description**: This study consisted of opioid dependent individuals aged 18 and older recruited from opioid substitution therapy clinics in the greater Sydney area and genetically unrelated individuals with little or no lifetime opioid misuse from neighborhoods in geographic proximity to these clinics. All subjects were of European-Australian descent. Additional details are available in^15^.

**Alcohol dependence measure**: All participants were assessed using the Semi-Structured Assessment for the Genetics of Alcoholism (SSAGA). Alcohol dependence was defined using DSM-IV criteria. For the purposes of these analyses, controls were defined as those who had a lifetime history of alcohol drinking but did not meet criteria for alcohol abuse or dependence. No other comorbid diagnoses were excluded.

**Acknowledgements**: Funding support for the CATS (dbGAP accession number: phs000277.v1.p1) was provided by the National Institute on Drug Abuse (R01 DA17305); GWAS genotyping services at the CIDR at The Johns Hopkins University were supported by the National Institutes of Health (contract N01-HG-65403).

**Center on Antisocial Drug Dependence (CADD)**

**Sample description**: GWAS participants were drawn from several primary studies described elsewhere^16,17,18,19^. The current sample of 1,901 unrelated adolescents was over-selected for adolescent BD, with half of the participants ascertained specifically from high-risk populations (i.e. recruited through substance abuse treatment, special schools, or involvement with the criminal justice system; see Supplement for additional criteria for clinical probands). CADD GWAS participants had an average age of 16.5 (SD = 1.4, range = 13.0–19.9), 28.9 % were female, and 37.3 % of participants reported non-Caucasian ancestry.

**Alcohol dependence measure**: Lifetime Alcohol Dependence was assessed with the CIDI-SAM and defined as meeting alcohol dependence at any wave for this longitudinal study.

**Acknowledgements**: The following grants supported data collection and analysis: DA011015, DA012845, DA021913, DA021905, DA032555, and DA035804.

**METHODS**

Quality Control (QC), imputation, and analysis were performed for each of the 11 studies in the alcohol dependence meta-analysis using ricopili (<https://github.com/Nealelab/ricopili>).

**QC:** Initial sample QC criteria included filters for call rate (< 0.98) and excess or depleted heterozygosity (|*F*| > 0.20). Samples were also checked for concordance between reported and genetically-inferred sex, except where insufficient chromosome X markers were available (GSMS, CHDS, and CADD). Variant QC criteria included filters for call rate (< 0.98), differential missingness between cases and controls (> 0.02), and departure from Hardy-Weinberg equilibrium (HWE) in cases (*p* < 1e-10) or controls (*p* < 1e-6). Additional filtering on minor allele frequency (MAF) was performed after imputation. Next, population outliers and cryptic relatedness were identified for exclusion. Relatedness coefficients ($\hat{\pi}$) and principal components analysis (PCA) were computed using a strictly defined subset of approximately independent autosomal SNPs (i.e. MAF > 0.05, HWE *p* > 1e-3, exclude MHC and chromosome 8 inversion regions, exclude strand ambiguous SNPs, and LD prune to *r^2^* < 0.20). Samples were filtered for cryptic relatedness $\hat{\pi}$ > 0.20 and PCA outliers and individuals with substantial non-European ancestry were removed. PCA with 1000 Genomes reference samples were compared to verify the selection of samples with European ancestry. Where necessary, additional study-specific QC criteria were also applied. To protect against potential effects of genotyping batch in Yale-Penn, variants were excluded if substantial differences in allele frequency in cases were observed between the two genotyping locations (*p* < 1e-4). Strand ambiguous SNPs, and SNPs that were invariant in data from either genotyping location were also excluded from the Yale-Penn data. For GESGA, variants were similarly excluded if allele frequency differences were observed across the 7 sample collection sites, conditional on phenotype (*p* < 1e-4).

**Imputation:** Prior to imputation, SNP locations were converted to genome build hg19 if required, and ricopili was used to verify alignment of each SNP’s chromosomal position to the 1000 Genomes phase 3 reference panel. SNPs were excluded from imputation if the reported alleles did not match the reference panel (without or without a strand flip), if the variant was strand ambiguous without a clear strand assignment from allele frequency, or if observed MAF differed substantially from the European allele frequency in 1000 Genomes. After alignment to the reference, samples were pre-phased using SHAPEIT. Imputation was then performed for each study using IMPUTE2 with the 1000 Genomes phase 3 reference panel.

**Association Analysis:** For each of the 11 studies of alcohol dependence, a GWAS for alcohol dependence status was performed using logistic regression with imputed dosages in PLINK2. The first five principal components were included as covariates within each study to control for population structure. Principal component covariates were computed using imputed best-guess genotypes filtered and LD pruned using the same criteria described in QC. Sex was also included as a covariate for all studies except GESGA (due to lack of female cases of alcohol dependence). Duplicated samples across studies were removed prior to analysis (*N*=16). The GWAS results from the 11 studies were then combined in an inverse-variance weighted meta-analysis using METAL. GWAS results were filtered for imputation INFO score (> 0.80), MAF (> 0.01), and MAF in cases (> 0.0025). Variants present in fewer than 7 of the 11 studies were also excluded. After filtering, the meta-analysis contains results for 8,524,330 variants with a sample size of *N*=11,424 (4,901 cases, 6,523 controls).

**Heritability Analysis:** LD score regression analysis was performed using HapMap3 SNPs and pre-computed LD scores from 1000 Genomes European reference samples (available from <https://data.broadinstitute.org/alkesgroup/LDSCORE/>)^20^. After filtering for MAF (> 0.05) and average imputation INFO score (> 0.90), and exclusion of structural variants and strand-ambiguous markers, 1,141,006 SNPs remained for LD score analysis. Conversion of $h_{g}^{2}$estimates from observed to liability scale was performed assuming a population prevalence of 0.13 for alcohol dependence.

------------------------------------------------------------------------------------------

**ANOREXIA NERVOSA (AN)**

**SAMPLES**

PGC-AN is a collaboration representing researchers and clinicians from around the world, founded with the goal of identifying the genetic risk factors involved in the etiology of anorexia nervosa (AN). The Freeze 1 sample (2016) comprises 3,495 individuals with AN and 10,982 controls from 12 separate cohorts.* Cases met DSM-IV criteria for either lifetime AN (restricting or binge-purge subtype) or lifetime eating disorders ‘not otherwise specified’ AN-subtype (i.e., exhibiting the core features of AN)^21^. Detailed information on recruitment and case ascertainment can be found elsewhere^22,23^. Out of the 12 cohorts, the largest single contributor (1,031 cases and 3,627 controls post-QC) was the Children’s Hospital of Philadelphia/Price Foundation collection, and these samples were included in a previous AN GWAS publication^22^. The remaining 11 cohorts were wholly or in part from the Welcome Trust Case Control Consortium 3 (WTCCC3). The Welcome Trust Sanger Institute genotyped cases for all WTCCC3 AN samples as well as controls for two of the samples, and these cases and controls were included in a previous AN GWAS meta-analysis^23^. Two of the 11 included cohorts included matched controls. As the WTCCC3 did not fund genotyping of controls, ancestrally matched controls for 9 out of 11 remaining WTCCC3 AN samples were sourced from multiple independent and overlapping research groups, consortia (including PGC), and funding bodies.

**METHODS**

**QC:** Genotyping of cases and controls was performed using Illumina arrays (Illumina, Inc., San Diego, CA). QC was performed on each of the 12 individual datasets using the updated version of PLINK^24^. Exclusion criteria for SNP-level QC comprised: (1) missingness > 0.02; (2) minor allele frequency < 0.05; (3) differential missingness between cases and controls > 0.02; and (4) HWE cutoff of p < 1x10^-6^ for controls and p < 1x10^-10^ for cases. Exclusion criteria for sample-level QC comprised: (1) missingness > 0.02; (2) FHET > |0.2|; (3) failed sex check; and (4) $\pî$ > 0.2 for relatedness. Principal components analysis (PCA) was first performed within each dataset and then across all datasets using FastPCA^25^, as implemented in the PGC pipeline^26^.

**Imputation:** Imputation to the 1000 Genomes phase 1^27^ reference was performed within the PGC pipeline using SHAPEIT^28^ for phasing and IMPUTE2 ^29^ for imputation. Imputation was performed with a chunk size of 3Mb and using default parameters on the full set of 2,186 phased haplotypes (August 2012, 30,069,288 variants, release “v3.macGT1”).

**Analysis:** Analysis within datasets was performed using an additive model in PLINK^24^, with the first ten principal components as covariates. Fixed-effects meta-analysis across the 12 datasets was accomplished using METAL^30^, with inverse variance weighting. QC, imputation, and primary GWAS were performed following the Ricopili pipeline^26^ at the Broad Institute.

*Note that the number of cohorts differs from the 15 cohorts presented in the original WTCCC3 publication by Boraska et al., 2014^23^. The reasons for this difference are as follows. First, cases from the United States and Canada were combined and analysed with United States controls. QC indicated that this was appropriate. Second, Sweden had contributed 39 cases to the initial GWAS. This sample size was below the threshold for inclusion so that cohort was not included. Third, samples from Italy (North) were not included due to the inability to identify appropriate controls.

**Acknowledgements**

Funding for anorexia nervosa has been from:

Children’s Hospital of Philadelphia/Price Foundation: We thank all patients and families enrolled in this study, as well as all healthy control who donated blood samples to Children’s Hospital of Philadelphia (CHOP) for genetic research purposes. We thank the Price Foundation for their support of recruiting patients, collecting clinical information and providing DNA samples used in this study. We also thank the Klarman Family Foundation for supporting the study. We thank the technical staff at the Center for Applied Genomics (CAG) at CHOP for generating genotypes used for analyses and the nursing, medical assistant and medical staff for their invaluable assistance with sample collection. Yiran Guo was funded by the 2011-2014 Davis Foundation Postdoctoral Fellowship Program in Eating Disorders Research Award. Dong Li was funded by the 2012-2015 Davis Foundation Postdoctoral Fellowship Program in Eating Disorders Research Award. Genome-wide genotyping for CHOP samples was funded by an Institutional Development Fund to CAG from CHOP. The study was additionally funded through the Electronic Medical Records and Genomics (eMERGE) Network (U01 HG006830) by National Human Genome Research Institute of National Institutes of Health, and also funded by donation from the Kurbert Family; The Wellcome Trust Wellcome Trust WT088827/Z/09; WT098051;

National Institutes of Health: K01MH093750; K01MH106675; K01MH109782; K02AA018755-06, R01AA015416-08, 5U01MH094432-04, 3U01MH094432-03S1; R01 MH109528; D0886501; R01 MH092793; This work received grants from EC Framework V Factors in Healthy Eating, from INRA/INSERM (4M406D), and from PHRC ENDANO (2008-A01636-49); European Commission (2008-2011) as an Early Stage Researcher from the Research Training Network INTACT (Individually Tailored Stepped Care for Women with Eating Disorders) in the Marie Curie Program (MRTN-CT-2006-035988); Veneto Region Grant BIOVEDA; Contract grant number: DGR 3984/08; Academy of Finland (28327); (Dr. Raevuori, grant number 259764);

The German Ministry for Education and Research (National Genome Research Net-Plus 01GS0820 and 01KU0903), the German Research Foundation (DFG; HI865/2-1), the European Community's Seventh Framework Programme (FP7/2007-2013) under grant agreement no. 245009 and no.262055." We thank the German Ministry for Education and Research for funding the ANTOP-study (project number 01GV0624)

AS was supported by the Federal Ministry of Education and Research (BMBF), Germany, FKZ 01EO1502; German Federal Ministry for Education and Research (BMBF) 01GV0601 and 01GV0624; German Ministry for Education and Research (National Genome Research Net-Plus 01GS0820) and the German Research Foundation (DFG; HI865/2-1); MHT received grant support from the Alexander von Humboldt Foundation, the Helmholtz Alliance ICEMED-Imaging and Curing Environmental Metabolic Diseases, through the Initiative and Networking Fund of the Helmholtz Association, the Helmholtz cross-program topic “Metabolic Dysfunction,” and the Deutsche Forschungsgemeinschaft (DFG-TS226/1-1 and TS226/3-1) and the European Research Council Consolidator Grant (HepatpMetaboPath)"

Ministry for Research and Education, Germany; The Helmholtz Alliance ICEMED-Imaging and Curing Environmental Metabolic Diseases, through the Initiative and Networking Fund of the Helmholtz Association, the Helmholtz cross-program topic “Metabolic Dysfunction,” and the Deutsche Forschungsgemeinschaft (DFG-TS226/1-1 and TS226/3-1); This work was supported by the Deutsche Forschungsgemeinschaft (EH 367/5-1 and SFB 940) and the Swiss Anorexia Nervosa Foundation;

AWB is supported by a Professional Services Agreement with the Regents of the University of California; Resnick Family Chair in Eating Disorders; Klarman Family Foundation; Research Council of Norway (RCN), and South-East Norway Regional Health Authority (SEN); Bergen Research Foundation, NFR (NORMENT-SFF), NCNG: This sample collection was supported by grants from the Bergen Research Foundation and the University of Bergen, the Dr Einar Martens Fund, the K.G. Jebsen Foundation, the Research Council of Norway, to SLH, VMS and TE; Supported by the Research Council of Norway (#248778, # 223273); The twin program of research at the Norwegian Institute of Public Health was supported by grants from The Norwegian Research Council and The Norwegian Foundation for Health and Rehabilitation; China Scholarship Council; Genome Canada, the government of Ontario, the Canadian Institutes of Health Research, University of Toronto McLaughlin Centre; Ontario Mental Health Foundation for funding the recruitment and collection of the DNA samples. Ministry of Health of Ontario AFP Innovation Fund; Grants 324715 and 480420 from the National Health and Medical Research Council (NHMRC) to TDW supported this work. Administrative support for data collection was received from the Australian Twin Registry, which is supported by an Enabling Grant (ID 310667) from the NHMRC administered by the University of Melbourne; Matthew Flinders Fellowship, Flinders University, South Australia, Australia Internal Grant Agency of the Ministry of Health of the Czech Republic IGA MZ _R NT 14094-3/2013;

Research of Korea Centers for Disease Control and Prevention Fund (code# HD16A1351); Nicole Soranzo's research is supported by the Wellcome Trust (Grant Codes WT098051 and WT091310), the EU FP7 (EPIGENESYS Grant Code 257082 and BLUEPRINT Grant Code†HEALTH-F5-2011-282510) and the National Institute for Health Research Blood and Transplant Research Unit (NIHR BTRU) in Donor Health and Genomics at the University of Cambridge in partnership with NHS Blood and Transplant (NHSBT). The views expressed are those of the author(s) and not necessarily those of the NHS, the NIHR, the Department of Health or NHSBT.

Psychiatry Research Trust (registered charity no. 284286); Swedish Research Council; Spanish Ministry of Economy and Competitiveness (MINECO) no. SAF2013-49108-R, the Generalitat de Catalunya AGAUR 2014 SGR-1138, the European Commission 7th Framework Program (FP7/2007-2013) 262055 (ESGI); Instituto de Salud Carlos III (FIS PI14/290 and CIBERobn Supported by MH CZ - DRO (MMCI, 00209805); An unrestricted grant from the Lundbeck Foundation, iPSYCH (Initiative for Integrative Psychiatric Research); and by Aarhus University for CIRRAU (Centre of Integrated Register-Based Research); This research was supported by a ZonMW VIDI Grant (91786327) from The Netherlands Organization for Scientific Research (NWO) to Prof. dr. Martien Kas; This study was supported by EU H2020 grants 692145, 676550, 654248, Estonian Research Council Grant IUT20-60, NIASC, EIT ñ Health and NIH-BMI Grant No: 2R01DK075787-06A1 and EU through the European Regional Development Fund (Project No. 2014-2020.4.01.15-0012 GENTRANSMED;

Ulrike Schmidt receives salary report from the National Institute of Health Research Mental Health Biomedical Research Centre at South London and Maudsley National Health Service Foundation Trust and King's College London; University of Otago Research Grant, New Zealand

------------------------------------------------------------------------------------------

**ATTENTION DEFICIT/HYPERACTIVITY DISORDER (ADHD)**

**SAMPLES**

The GWAS meta-analysis of ADHD results used in the SNP heritability analysis, include samples from the Danish iPSYCH initiative, and 10 samples from the Psychiatric Genomics Consortium (PGC). The iPSYCH ADHD sample is a nationwide population based case-cohort sample. Cases and controls were identified based on information in the Danish Psychiatric Central Research Register^31^ and controls were randomly selected from the same nationwide birth cohort and not diagnosed with ADHD. In order to obtain DNA for genotyping the identified cases and controls were linked to samples in the Danish Newborn Screening Biobank through the unique personal identification number, which is assigned to all live-born babies in Denmark.

The samples from the PGC were all of European Ancestry and consists of four trio samples and six case control samples. The four trio samples include: 1) The CHOP (Children’s Hospital of Philadelphia) ADHD trio sample (262 trios), which were recruited from pediatric and behavioral health clinics in the Philadelphia area^32^; 2) The IMAGE-I (International Multisite ADHD Genetics Project) trio samples^33,34^ (700 trios), collected from countries in and around Europe including Belgium, Germany, Ireland, the Netherlands, Spain, Switzerland, and the United Kingdom, and Israel; 3) the PUWMa (Pfizer-funded study from the University of California, Los Angeles (UCLA), Washington University, and Massachusetts General Hospital (MGH)) trio samples^35^ (563 trios), which were collected independently at those three sites using similar but slightly different methods; 4) The Toronto, Canadian ADHD trio sample^36^ (109 trios), which was drawn from an outpatient clinic in an urban pediatric hospital and included children who were referred for attention, learning and/or behavioral problems.

The six case control samples include:

1) The Barcelona sample^37^ (572 cases and 425 controls), which comprised ADHD cases recruited and evaluated at the Hospital Universitari Vall d’Hebron located in Barcelona (Spain). The control sample consisted of unrelated blood donors frequency-matched for gender with the ADHD cases and screened to exclude those with lifetime ADHD symptoms or diagnosis; 2) The Bergen, Norway sample^38^ (295 cases and 202 controls) consisted of patients recruited through a Norwegian national medical registry, as well as by psychologists and psychiatrists working at out-patient clinics. The controls were recruited through the Medical Birth Registry of Norway; 3) The Cardiff sample^39^ (721 cases, 5081 controls) consisted of cases recruited from community clinics in Cardiff, Wales, St. Andrews, Scotland and Dublin, Ireland. Controls were obtained from the Wellcome Trust Case Control Consortium–Phase 2; 4) The German (Würzburg) sample^40^ (487 cases and 1290 controls) comprised patients with ADHD recruited and phenotypically characterized in six psychiatric outpatient units for children and adolescents. The controls were drawn from three population based epidemiological studies: (a) the Heinz Nixdorf Recall (Risk Factors, Evaluation of Coronary Calcification, and Lifestyle) study 3, (b) PopGen, (c) KORA (Cooperative Health Research in the Region of Augsburg); 5) The IMAGE-II ADHD sample^41^ (624 cases, 1755 controls) included cases collected using similar but not identical methods as the IMAGE-I study. The controls were collected for an IRB approved GWAS of schizophrenia which have been described elsewhere ^42^; 6) The Yale-Penn sample (182 cases, 1315 controls) consists of small nuclear families and unrelated individuals, collected to study the genetics of substance dependence ^84344^. The case-control subjects were recruited from substance abuse treatment centers and through advertisements at the University of Connecticut Health Center, Yale University School of Medicine, the Medical University of South Carolina, the University of Pennsylvania, and McLean Hospital.

**METHODS**

Quality control, imputation and primary association analyses were done using the bioinformatic pipeline “Ricopili”^26^. The 10 PGC samples were processed separately and the iPSYCH sample was processed in 23 separate waves.

Stringent quality control was applied to each sample before imputation, following standard procedures for GWAS^26^. Imputing was done using SHAPEIT^28^ to estimate haplotypes and subsequently IMPUTE2^45^ for imputing genotypes. Haplotypes the 1000 Genomes Project, phase 3^46,20^ was used as reference data. Trio imputation was done with a case-pseudocontrol setup.

Related individuals and genetic outliers were identified based on a set of high quality markers (~30,000) pruned for linkage disequilibrium (LD). Related individuals were identified by an “identity by state analysis” using PLINK v1.9^24,47^ and one individual was excluded in pairs of subjects with $\hat{\pi}$ > 0.2. Genetic outliers were identified and subsequently removed based on principal component analyses performed using smartPCA^48^. This was done separately for each of the PGC samples and on a merged set of genotypes for the iPSYCH sample.

Association analyses using dosage data were performed for the 10 PGC samples and the 23 waves in iPSYCH by an additive logistic regression model using PLINK v1.9^24,47^ and relevant principal components as covariates. The results were meta-analysed using an inverse-weighted fixed effects mode implemented in the software METAL (version 2011-03-25)^30^. Only SNPs with imputation quality (INFO score) > 0.8 and maf > 0.01 were included. The meta-analysis included, in total 19,099 cases and 34,194 controls and 8,094,095 markers (only markers supported by an effective sample size (Neff = 2/(1/Ncases + 1/Ncontrols)^49^ greater than 70% were included).

**Heritability analysis**

SNP heritability was estimated using LD score regression^50^. Summary statistics from the ADHD GWAS meta-analysis and pre-computed LD scores for HapMap3 SNPs calculated based on 378-phased European-ancestry individuals from the 1000 Genomes Project were used in the analysis (LD scores available on <https://github.com/bulik/ldsc>). Only results for markers with an imputation INFO score > 0.90 were included in the analysis. After filtering and merging with the panel of high confidence HapMap3 SNPs 1,065,687 SNPs were included in the analysis. The SNP heritability for ADHD was calculated on the liability scale using a prevalence of ADHD of 5% in the population^51^.

**Acknowledgements**

The iPSYCH ADHD study was supported by The Lundbeck Foundation (grant no R102-A9118 and R155-2014-1724), Denmark; the Stanley Medical Research Institute; an Advanced Grant from the European Research Council (project no: 294838); the Stanley Center for Psychiatric Research at Broad Institute and Centre for Integrated Register-based Research at Aarhus University. This research has been conducted using the Danish National Biobank resource, supported by the Novo Nordisk Foundation.

------------------------------------------------------------------------------------------

**AUTISM SPECTRUM DISORDER (ASD)**

**SAMPLES**

The ASD summary statistics are based on a meta-analysis of the results based on 5305 trios of European ancestry from the Autism Spectrum Disorder Working Group of the PGC Consortium and 13076 cases and 22664 controls of European ancestry from the iPSYCH autism sample.

A detailed description of the PGC sample is available on the PGC web site: <https://www.med.unc.edu/pgc/files/resultfiles/PGCASDEuro_Mar2015.readme.pdf>. Briefly, five cohorts provided genotypes (n denote the number of trios for which genotypes were available): The Geschwind Autism Center of Excellence (ACE; n = 391), the Autism Genome Project (AGP; n = 2272)^52^, the Autism Genetic Resource Exchange (AGRE; n = 974)^53^, the NIMH Repository (<https://www.nimhgenetics.org/available_data/autism/>), the Montreal/Boston Collection (MONBOS; n = 1396)^54^, and the Simons Simplex Collection (SSC; n = 2231)^55^. The trios were analyzed as cases and pseudo controls.

The iPSYCH ASD sample is a population based case-cohort sample extracted from the birth cohorts consisting of all children born in Denmark between May 1^st^ 1981 and December 31^st^ 2005. Eligible were singletons born to a known mother and resident in Denmark on their one-year birthday. Cases were defined from the Danish Psychiatric Central Research Register^31^ as those having an ASD diagnosis (ICD codes F84.0, F84.1, F84.5, F84.8 or F84.9) given no later than 2013. The controls constitute a random sample from the set of eligible children that did not have an ASD diagnosis by 2013. The samples were linked using the unique personal identification number to the Danish Newborn Screening Biobank. Genotypes are available on 14970 cases and 26125 controls.

**METHODS**

Data processing and QC was conducted according to the standards employed by the PGC Statistical Analysis Group and carried out using their pipeline Ricopili^26^. To minimize potential batch effects the data was processed separately in the 23 genotyping batches in the case of iPSYCH and for each cohort in the PGC sample. Phasing was achieved using SHAPEIT^28^ and imputation done by IMPUTE2^56^ with haplotypes from the 1000 Genomes Project, phase 3 (1kGP3) as reference^57^. Trio samples were imputed as a case-pseudo-controls design.

Prior to principal component analysis (PCA), regions of high LD^58^ were excluded and genotypes were pruned down to a set of roughly 30k markers by pruning in a sliding window fashion using plink 1.9^47^. Using Plink’s identity by state analysis, pairs of subjects were identified with $\hat{\pi}>0.2$ and one subject of each such pair excluded at random keeping cases when possible. PCA was carried out using smartPCA^48^. In iPSYCH a subsample of European ancestry was selected using a ellipsoid in the space of the first 3 principal components (PCs) centred and scaled using the mean and 8 standard deviation of the PCs restricted to the subsample whose parents and grandparents were all known to have been born in Denmark (n=31500). In the PGC sample a Euclidian distance measure weighted by the variance explain for each of the first 3 PCs. Individuals more distant than 10 standard deviations from the combined CEU and TSI HapMap reference populations were excluded. For both iPSYCH and PGC samples we conducted a secondary PCA to provide covariates for the association analyses.

Association analyses were done applying plink 1.9 to the imputed dosage data for each iPSYCH batch at a time and each PGC subcohort at a time. The results were subsequently meta analyzed using METAL^30^(July 2010 version) employing an inverse variance weighted fixed effect model^59^. Prior to release we filtered the summary statistics allowing only markers with an imputation info score $\geq0.7$, maf $\geq0.01$ and an effective sample size of at least 70% of the maximum. The effective sample size was estimated from the number of cases, Nca, and controls, Nco, contributing to the individual regression as 2*Nca*Nco/(Nca+Nco).

SNP heritability was estimated using LD score regression (LDSC)^50^. For LDSC we used precomputed LD scores based on the European ancestry samples of the 1000 Genomes Project^57^ restricted to HapMap3 SNPs^60^(downloaded from the <https://github.com/bulik/ldsc>). The summary stats with standard LDSC filtering were regressed onto these scores. For liability scale estimates, we used a population prevalence of 1.22%^61^.

**Acknowledgements:**

The iPSYCH ASD study was supported by The Lundbeck Foundation (grant no R102-A9118 and R155-2014-1724), Denmark; the Stanley Medical Research Institute; an Advanced Grant from the European Research Council (project no: 294838); the Stanley Center for Psychiatric Research at Broad Institute and Centre for Integrated Register-based Research at Aarhus University. This research has been conducted using the Danish National Biobank resource, supported by the Novo Nordisk Foundation.

------------------------------------------------------------------------------------------

**BIPOLAR DISORDER (BIP)**

**SAMPLES**

This paper reports the results of genomic analyses on 20,352 bipolar disorder cases and 31,358 controls (51,710 subjects). All individual genotypes in the discovery GWAS were directly processed and analysed by the PGC-BIP2.

Case definitions

The sections below describe the bipolar disorder samples that were part of this report. We also describe the ascertainment procedures and diagnosis of the subjects comprising this report. As in our previous mega-analysis (PGC1)^62^, individuals with schizoaffective disorder bipolar type were included as cases since family history studies have shown coaggregation of these two disorders, diagnostic criteria separating them are subjective, and the inter-rater reliability is often low across research groups^63,64^.

The lead-PI of each sample warranted that their protocol was approved by their local Ethical Committee and that all subjects provided written informed consent.

All of these subjects are independent as confirmed using SNPs directly genotyped in all samples.

Most studies have been described in detail in the citations provided. The boldfaced first line for each sample is study PI, PubMed ID, country (study name), and the PGC internal tag or study identifier.

**Adolfsson, R | Not published | Umeå, Sweden | bip_ume4_eur**

Clinical characterization of the patients included the Mini-International Neuropsychiatric Interview (MINI^65^), the Diagnostic Interview for Genetic Studies (DIGS^66^), the Family Interview for Genetic Studies (FIGS^67^) and the Schedules for Clinical Assessment in Neuropsychiatry (SCAN^68^). The final diagnoses were made according to the DSM-IV-TR^21^ and determined by consensus of 2 research psychiatrists. The unrelated Swedish control individuals, consisting of a large population-based sample representative of the general population of the region, were randomly selected from the ‘Betula study’^69^.

**Alda, M; Smoller, J | Not published | Nova Scotia, Canada; I2B2 controls | bip_hal2_eur**

The case samples were recruited from patients longitudinally followed at specialty mood disorders clinics in Halifax and Ottawa (Canada). Cases were interviewed in a blind fashion with the Schedule of Affective Disorders and Schizophrenia-Lifetime version (SADS-L)^70^ and consensus diagnoses were made according to DSM-IV^21^ and Research Diagnostic Criteria (RDC)^71^. Protocols and procedures were approved by the local Ethics Committees and written informed consent was obtained from all patients before participation in the study. Control subjects were drawn from the I2B2 (Informatics for Integrating Biology and the Bedside) project ^72^. The study consists of de-identified healthy individuals recruited from a healthcare system in the Boston, MA, US area. The de-identification process meant that the Massachusetts General Hospital Institutional Review Board elected to waive the requirement of seeking informed consent as detailed by Code of Federal Regulations, Title 45, Part 46, Section 116 (46.116).

**Andreassen, O | 21926972 [PGC1] | Norway (TOP) | bip_top7_eur**

In the TOP study (Tematisk omrade psykoser), cases of European ancestry, born in Norway, were recruited from psychiatric hospitals in the Oslo region. Patients were diagnosed according to the SCID^73^ and further ascertainment details have been reported. Healthy control subjects were randomly selected from statistical records of persons from the same catchment area as the patient groups. The control subjects were screened by interview and with the Primary Care Evaluation of Mental Disorders (PRIME-MD)^74^. None of the control subjects had a history of moderate/severe head injury, neurological disorder, mental retardation or an age outside the age range of 18-60 years. Healthy subjects were excluded if they or any of their close relatives had a lifetime history of a severe psychiatric disorder. All participants provided written informed consent and the human subjects protocol was approved by the Norwegian Scientific-Ethical Committee and the Norwegian Data Protection Agency.

**Andreassen, O | Not published | Norway (TOP) | bip_top8_eur**

The TOP8 bipolar disorder cases and controls were ascertained in the same way as the bip_top7_eur (TOP7) samples described above.

**Biernacka, J | 27769005 | Mayo Clinic, USA | bip_may1_eur**

Bipolar cases were drawn from the Mayo Clinic Bipolar Biobank^75^. Enrolment sites included Mayo Clinic, Rochester, Minnesota; Lindner Center of HOPE/University of Cincinnati College of Medicine, Cincinnati, Ohio; and the University of Minnesota, Minneapolis, Minnesota. Enrolment at each site was approved by the local Institutional Review Board approval, and all participants consented to use of their data for future genetic studies. Participants were identified through routine clinical appointments, from in-patients admitted in mood disorder units, and recruitment advertising. Participants were required to be between 18 and 80 years old and be able to speak English, provide informed consent, and have DSM-IV-TR^21^ diagnostic confirmation of type I or II bipolar disorder or schizoaffective bipolar disorder as determined using the SCID^73^. Controls were selected from the Mayo Clinic Biobank^76^. Potential controls with ICD9 codes for bipolar disorder, schizophrenia or related diagnoses in their electronic medical record were excluded.

**Blackwood, D | 18711365 [PGC1] | Edinburgh, UK | bip_edi1_eur**

This sample comprised Caucasian individuals contacted through the inpatient and outpatient services of hospitals in South East Scotland. A BD-I diagnosis was based on an interview with the patient using the SADS-L^70^ supplemented by case note review and frequently by information from medical staff, relatives and care givers. Final diagnoses, based on DSM-IV^21^ criteria were reached by consensus between two trained psychiatrists. Ethnically-matched controls from the same region were recruited through the South of Scotland Blood Transfusion Service. Controls were not directly screened to exclude those with a personal or family history of psychiatric illness. The study was approved by the Multi-Centre Research Ethics Committee for Scotland and patients gave written informed consent for the collection of DNA samples for use in genetic studies.

**Corvin, A | 18711365 [PGC1] | Ireland | bip_dubl_eur**

Samples were collected as part of a larger study of the genetics of psychotic disorders in the Republic of Ireland, under protocols approved by the relevant IRBs and with written informed consent that permitted repository use. Cases were recruited from Hospitals and Community psychiatric facilities in Ireland by a psychiatrist or psychiatric nurse trained to use the SCID^73^. Diagnosis was based on the structured interview supplemented by case note review and collateral history where available. All diagnoses were reviewed by an independent reviewer. Controls were ascertained with informed consent from the Irish GeneBank and represented blood donors who met the same ethnicity criteria as cases. Controls were not specifically screened for psychiatric illness.

**Craddock, N | 17554300 | WTCCC | bip_wtcc_eur_sr-qc**

Cases were all over the age of 16 years old, living in mainland UK and of European descent. Recruitment was undertaken throughout the UK and included individuals who had been in contact with mental health services and had a lifetime history of high mood. After providing written informed consent, participants were interviewed by a trained psychologist or psychiatrist using a semi-structured lifetime diagnostic psychiatric interview and available psychiatric medical records were reviewed. Using all available data, best-estimate life-time diagnoses were made according to the RDC^71^.   In the current study we included cases with a lifetime diagnosis of RDC bipolar I disorder, bipolar II disorder or schizo-affective disorder, bipolar type.

Controls were recruited from two sources: the 1958 Birth Cohort study and the UK Blood Service (blood donors) and were not screened for history of mental illness.

All cases and controls were recruited under protocols approved by the appropriate IRBs. All subjects gave written informed consent.

**Craddock, N; Jones, I; Jones, L | [ICCBD] | Cardiff, UK (ICCBD-BDRN) | bip_icuk_eur**

Cases were recruited via systematic and not systematic methods as part of the Bipolar Disorder Research Network project ([www.bdrn.org](http://www.bdrn.org)), provided written informed consent and were interviewed using a semi-structured diagnostic interview, the Schedules for Clinical Assessment in Neuropsychiatry, a life chart and detailed information about family history of psychiatric disorders. Based on the information gathered from the interview, case notes review and questionnaires, best-lifetime diagnosis was made according to DSM-IV^21^. Inter-rater reliability was formally assessed using 20 randomly selected cases (mean ĸ Statistic = 0.85). In the current study we included cases with a lifetime diagnosis of DSM-IV bipolar disorder or schizo-affective disorder, bipolar type. The BDRN study received approval from the Multi-Region and Local Research Ethics Committee in the United Kingdom.

Controls were part of the Wellcome Trust Case Control Consortium common control set, which comprised healthy blood donors recruited from the UK Blood Service and samples from the 1958 British Birth Cohort. Controls were not screened for a history of mental illness.

All cases and controls were recruited under protocols approved by the appropriate IRBs. All subjects gave written informed consent.

**Hauser, J; Lissowska, J; Forstner, AJ | 24618891 | BOMA-Poland | bip_bmpo_eur**

Cases were recruited at the Department of Psychiatry, Poznan University of Medical Sciences, Poznan, Poland. All cases received a lifetime diagnosis of BD according to the DSM-IV^21^ criteria on the basis of a consensus best-estimate procedure^77^ and structured diagnostic interviews using the SCID^73^. Controls were drawn from a population-based case-control sample recruited by the Cancer-Center and Institute of Oncology, Warsaw, Poland and a hospital-based case-control sample recruited by the Nofer Institute of Occupational Medicine, Lodz, Poland. The Polish controls were produced by the International Agency for Research on Cancer (IARC) and the Centre National de Génotypage (CNG) GWAS Initiative for a study of upper aerodigestive tract cancers ^78^. The controls were not screened for a history of mental illness. Study protocols were reviewed and approved in advance by Institutional Review Boards of the participating institutions. All subjects provided written informed consent.

**Fullerton, J.M.; Mitchell, P.B.; Schofield, P.R.; Martin N.G. | 24618891 | BOMA-Australia | bip_bmau_eur**

Cases were recruited at the Mood Disorder Unit, Prince of Wales Hospital in Sydney. All cases received a lifetime diagnosis of BD according to the DSM-IV^21^ criteria on the basis of a consensus best-estimate procedure^77^ and structured diagnostic interviews using the DIGS^66^, FIGS^67^, and the SCID^73^. Controls were parents of unselected adolescent twins from the Brisbane Longitudinal Twin Study^79^. The controls were not screened for a history of mental illness. Study protocols were reviewed and approved in advance by Institutional Review Boards of the participating institutions. All subjects provided written informed consent.

**Grigoroiu-Serbanescu, M; Nöthen, MM | 21353194 | BOMA-Romania | bip_rom3_eur**

Cases were recruited from consecutive admissions to the Obregia Clinical Psychiatric Hospital, Bucharest. Patients were administered the DIGS^66^ and FIGS^67^ interviews. Information was also obtained from medical records and close relatives. The diagnosis of BP-I was assigned according to DSM-IV^21^ criteria using the best estimate procedure^77^. All patients had at least two hospitalized illness episodes. Population-based controls were evaluated using the DIGS^66^ to exclude a lifetime history of major affective disorders, schizophrenia, schizoaffective disorders, and other psychoses, obsessive-compulsive disorder, eating disorders, and alcohol or drug addiction.

**Kelsoe, J | 21926972 [PGC1] | USA (GAIN) | bip_gain_eur**

*Genetic Association Information Network (GAIN)/ The Bipolar Genome Study (BiGS)* The BD sample was collected under the auspices of the NIMH Genetics Initiative for BD (<http://zork.wustl.edu/nimh/>), genotyped as part of GAIN and analyzed as part of a larger GWAS conducted by the BiGS consortium. Approximately half of the GAIN sample was collected as multiplex families or sib pair families (waves 1-4), the remainder were collected as individual cases (wave 5). Subjects were ascertained at 11 sites: Indiana University, John Hopkins University, the NIMH Intramural Research Program, Washington University at St. Louis, University of Pennsylvania, University of Chicago, Rush Medical School, University of Iowa, University of California, San Diego, University of California, San Francisco, and University of Michigan. All investigations were carried out after the review of protocols by the IRB at each participating institution. At all sites, potential cases were identified from screening admissions to local treatment facilities and through publicity programs or advocacy groups. Potential cases were evaluated using the DIGS^66^, FIGS^67^, and information from relatives and medical records. All information was reviewed through a best estimate diagnostic procedure by two independent and non-interviewing clinicians and a consensus best-estimate diagnosis was reached. In the event of a disagreement, a third review was done to break the tie. Controls were from the NIMH Genetic Repository sample obtained by Dr. P. Gejman through a contract to Knowledge Networks, Inc. Only individuals with complete or near-complete psychiatric questionnaire data who did not fulfill diagnostic criteria for major depression and denied a history of psychosis or BD were included as controls for BiGS analyses. Controls were matched for gender and ethnicity to the cases.

**Kelsoe, J; Sklar, P; Smoller, J | [PGC1 Replication] | USA (FAT2; FaST,** **BiGS, TGEN) | bip_fat2_eur**

Cases were collected from individuals at the 11 U.S. sites described for the GAIN sample. Eligible participants were age 18 or older meeting DSM-IV criteria for BD-I or BD-II by consensus diagnosis based on interviews with the Affective Disorders Evaluation (ADE)^80^ and MINI^65^. All participants provided written informed consent and the study protocol was approved by IRBs at each site. Collection of phenotypic data and DNA samples were supported by NIMH grants MH063445 (JW Smoller); MH067288 (PI: P Sklar), and MH63420 (PI: V Nimgaonkar). The control samples were NIMH controls that were using the methods described in that section. The case and control samples were independent of those included in the GAIN sample.

**Kirov, G | 25055870 | Bulgarian trios | bip_butr_eur**

All cases were recruited in Bulgaria from psychiatric inpatient and outpatient services. Each proband had a history of hospitalisation and was interviewed with an abbreviated version of the SCAN^68^. Consensus best-estimate diagnoses were made according to DSM-IV^21^ criteria by two researchers. All participants gave written informed consent and the study was approved by local ethics committees at the participating centers.

**Kirov, G | 25055870 | UK trios | bip_uktr_eur**

The BD subjects were recruited and interviewed in person by a senior psychiatrist, using abbreviated version of the SCAN^68^. Consensus best-estimate diagnoses were made based on the interview and hospital notes. Ethics committee approval for the study was obtained from the relevant research ethics committees and all individuals provided written informed consent for participation.

**Landen, M | [ICCBD] | Sweden (ICCBD) | bip_swa2_eur**

The BD subjects were identified using the Swedish Hospital Discharge Register

including subjects with at least two hospitalizations with a BD diagnosis and confirmatory diagnostic review in a subset of subjects. Additional subjects were recruited from the Affective Center at St Goran Hospital in Stockholm, Sweden, following physician's referral for BD. The diagnostic instrument used was a Swedish adaptation of the ADE^80^ which includes the affective module of the SCID^73^. Further BD cases were recruited from the Stockholm County catchment area. Diagnoses were made according to the DSM-IV criteria, and cases were not reported previously. The control subjects used were the same as for the SCZ analyses described above. All ascertainment procedures were approved by the Regional Ethical Committees in Sweden.

**Landen, M | [ICCBD] | Sweden (ICCBD) | bip_swei_eur**

The cases and controls in the bip_swei_eur sample were recruited using the same ascertainment methods described for the bip_swa2_eur sample.

**Leboyer, M | [PGC1 replication] | France | bip_fran_eur**

Cases with BD-I or BD-II and control samples were recruited as part of a large study of genetics of BD in France (Paris-Creteil, Bordeaux, Nancy) with a protocol approved by relevant IRBs and with written informed consent. Cases were of French descent for more than 3 generations were assessed by a trained psychiatrist or psychologist with the DIGS^66^ and the FIGS^67^. Diagnoses were based on structured interviews supplemented by medical case notes, mood scales and self-rating questionnaire assessing dimensions.

**Li, Q | 24166486; 27769005** **| USA (Janssen), SAGE controls | bip_jst5_eur**

The study included unrelated patients with bipolar I disorder from 6 clinical trials (IDs: NCT00253162, NCT00257075, NCT00076115, NCT00299715, NCT00309699, and NCT00309686)^81,82,83,84,85,86^. Participant recruitment was conducted by Janssen Research & Development, LLC (formerly known as Johnson & Johnson Pharmaceutical Research & Development, LLC) to assess the efficacy and safety of risperidone. Bipolar cases were diagnosed according to DSM-IV-TR ^21^ criteria. The diagnosis of bipolar disorder was confirmed by the Schedule for Affective Disorders and Schizophrenia for School-Age Children-Present and Lifetime Version (K-SADS-PL)^87^ in NCT00076115, by the SCID^73^ in NCT00257075 and NCT00253162, or by the MINI^65^ in NCT00299715 and NCT00309699, and NCT00309686, respectively. Additional detailed descriptions of these clinical trials can be found at ClinicalTrials.gov. Only patients of European ancestry with matching controls were included in the current analysis. Controls subjects were drawn from the Study of Addiction: Genetics and Environment (SAGE, dbGaP Study Accession: phs000092.v1.p1). Control subjects did not have alcohol dependence or drug dependence diagnoses; however, mood disorders were not an exclusion criterion^14^.

**McQuillin, A; Gurling, H | 18317468 [PGC1] | UCL (University College London), London, UK | bip_uclo_eur**

The UCL sample comprised Caucasian individuals who were ascertained and received clinical diagnoses of bipolar disorder according to UK National Health Service (NHS) psychiatrists at interview using the categories of the International Classification of Disease version 10. In addition bipolar subjects were included only if both parents were of English, Irish, Welsh or Scottish descent and if three out of four grandparents were of the same descent. All volunteers read an information sheet approved by the Metropolitan Medical Research Ethics Committee who also approved the project for all NHS hospitals. Written informed consent was obtained from each volunteer. The UCL control subjects were recruited from London branches of the National Blood Service, from local NHS family doctor clinics and from university student volunteers. All control subjects were interviewed with the SADS-L^70^ to exclude all psychiatric disorders.

**Ophoff, RA | Not Published | Netherlands | bip_ucla_eur**

The case sample consisted of inpatients and outpatients recruited through psychiatric hospitals and institutions throughout the Netherlands^88^. Cases with DSM-IV^21^ bipolar disorder were included in the analysis. Controls were collected in parallel at different sites in the Netherlands and were volunteers with no psychiatric history. Ethical approval was provided by UCLA and local ethics committees and all participants gave written informed consent.

**Paciga, S | [PGC1] | USA (Pfizer) | bip_pf1e_eur**

This sample comprised Caucasian individuals recruited into one of three Geodon (ziprasidone) clinical trials (NCT00141271, NCT00282464, NCT00483548). Subjects were diagnosed by a clinician with a primary diagnosis of Bipolar I Disorder, most recent episode depressed, with or without rapid cycling, without psychotic features, as defined in the DSM-IV-TR^21^ (296.5x) and confirmed by the MINI^65^ (version 5.0.0).  Subjects also were assessed as having a HAM-D-17 total score of >20 at the screening visit.  The trials were conducted in accordance with the protocols, International Conference on Harmonization of Good Clinical Practice Guidelines, and applicable local regulatory requirements and laws.  Patients gave written informed consent for the collection of blood samples for DNA for use in genetic studies.

**Pato, C | [ICCBD] | Los Angeles, USA (ICCBD-GPC)| bip_usc2_eur**

Genomic Psychiatry Consortium (GPC) cases and controls were collected via the University of Southern California healthcare system, as previously described^89^. Using a combination of focused, direct interviews and data extraction from medical records, diagnoses were established using the OPCRIT and were based on DSM-IV-TR criteria^21^. Age and gender-matched controls were ascertained from the University of Southern California health system and assessed using a validated screening instrument and medical records.

**Reif, A; Nöthen, MM; Forstner, AJ | 24618891 | BOMA-Germany II | bip_bmg2_eur**

Cases were recruited from consecutive admissions to psychiatric in-patient units at the University Hospital Würzburg. All cases received a lifetime diagnosis of BD according to the DSM-IV^21^ criteria using a consensus best-estimate procedure^77^ based on all available information, including semi-structured diagnostic interviews using the Association for Methodology and Documentation in Psychiatry (AMDP)^90^, medical records and the family history method. In addition, the OPCRIT^91^ system was used for the detailed polydiagnostic documentation of symptoms.

Control subjects were unaffected subjects were drawn from the population-based Heinz Nixdorf Recall (HNR) Study^92^. The controls were not screened for a history of mental illness. Study protocols were reviewed and approved in advance by Institutional Review Boards of the participating institutions. All subjects provided written informed consent.

**Rietschel, M; Nöthen, MM, Cichon, S | 21926972 [PGC1] | BOMA-Germany I | bip_bonn_eur**

Cases for the BOMA-Bipolar Study were ascertained from consecutive admissions to the inpatient units of the Department of Psychiatry and Psychotherapy at the University of Bonn and at the Central Institute for Mental Health in Mannheim, University of Heidelberg, Germany. DSM-IV lifetime diagnoses of bipolar I disorder were assigned using a consensus best-estimate procedure, based on all available information, including a structured interview with the SCID^73^ and SADS-L^70^, medical records, and the family history method. In addition, the OPCRIT^91^ checklist was used for the detailed polydiagnostic documentation of symptoms. Controls were ascertained from three population-based studies in Germany (PopGen, KORA, and Heinz-Nixdorf-Recall Study). Study protocols were reviewed and approved in advance by Institutional Review Boards of the participating institutions. All subjects provided written informed consent.

**Rietschel, M; Nöthen, MM; Rivas, F; Mayoral, F; Kogevinas, M; others | 24618891 | BOMA-Spain | bip_bmsp_eur**

Cases were recruited at the mental health departments of the following five centers in Andalusia, Spain: University Hospital Reina Sofia of Córdoba, Provincial Hospital of Jaen; Hospital of Jerez de la Frontera (Cádiz); Hospital of Puerto Real (Cádiz); Hospital Punta Europa of Algeciras (Cádiz); and Hospital Universitario San Cecilio (Granada). Diagnostic assessment was performed using the SADS-L^70^; the OPCRIT^91^; a review of medical records; and interviews with first and/or second degree family members using the Family Informant Schedule and Criteria (FISC) . Consensus best estimate BD diagnoses were assigned by two or more independent senior psychiatrists and/or psychologists, and according to the RDC^71^, and the DSM-IV^21^. Controls were Spanish subjects drawn from a cohort of individuals recruited in the framework of the European Community Respiratory Health Survey (ECRHS, http://www.ecrhs.org/). The controls were not screened for a history of mental illness. Study protocols were reviewed and approved in advance by Institutional Review Boards of the participating institutions. All subjects provided written informed consent.

**Rietschel, M; Nöthen, MM; Schulze, TG; Bauer, M; Forstner, AJ; Müller-Myhsok, B | 24618891 | BOMA-Germany III | bip_bmg3_eur**

Cases were recruited at the Central Institute of Mental Health in Mannheim, University of Heidelberg, and other collaborating psychiatric hospitals in Germany. All cases received a lifetime diagnosis of BD according to the DSM-IV^21^ criteria using a consensus best-estimate procedure^77^ based on all available information including structured diagnostic interviews using the AMDP^90^, Composite International Diagnostic Screener (CID-S)^93^, SADS-L^70^ and/or SCID^73^, medical records, and the family history method. In addition, the OPCRIT^91^ system was used for the detailed polydiagnostic documentation of symptoms.

Controls were selected randomly from a Munich-based community sample and recruited at the Max-Planck Institute of Psychiatry. They were screened for the presence of anxiety and mood disorders using the CID-S^93^. Only individuals without mood and anxiety disorders were collected as controls. Study protocols were reviewed and approved in advance by Institutional Review Boards of the participating institutions. All subjects provided written informed consent.

**Scott, L | [PGC1] | Michigan, USA (Pritzker and NIMH) | bip_mich_eur**

The Pritzker Neuropsychiatric Disorders Research Consortium (NIMH/Pritzker) case and controls samples were from the NIMH Genetics Initiative Genetics Initiative Repository. Cases were diagnosed according to DMS-III or DSM-IV criteria using the DIGS^66^ or FIGS^67^ and/or medical record review. Cases with low confidence diagnoses were excluded. From each wave 1-5 available non-Ashkenazi European-origin family, two BD-I siblings were included when possible and the proband was preferentially included if available (n=946 individuals in 473 sibling pairs); otherwise a single BD-I case was included (n=184). The bipolar sibling pairs were retained within the NIMH/Pritzker sample when individuals in more than one study were uniquely assigned to a study set. Controls had non-Ashkenazi European-origin, were aged 20-70 years and reported no diagnosis with or treatment for BD or schizophrenia, and that they had not heard voices that others could not hear. Individuals with suspected major depression were excluded based on answers to questions related to depressive mood. NIMH controls were further selected as the best match(es) to NIMH cases based on self-reported ancestry in the DIGS^66^.

**Sklar, P; Smoller, J | 18317468 [PGC1] | USA (STEP1) | bip_stp1_eur**

The Systematic Treatment Enhancement Program for Bipolar Disorder (STEP-BD) was a seven-site, national U.S., longitudinal cohort study designed to examine the effectiveness of treatments and their impact on the course of BD that enrolled 4,361 participants who met DSM-IV criteria for BD-I, BD-II, bipolar not otherwise specified (NOS), schizoaffective manic or bipolar type, or cyclothymic disorder based on diagnostic interviews. From the parent study, 2,089 individuals who were over 18 years of age with BD-I and BD-II diagnoses consented to the collection of blood samples for DNA. BD samples with a consensus diagnosis of BD-I on both the ADE^80^ and MINI^65^ were selected for inclusion in STEP1. Two groups of controls samples from the NIMH repository were used. One comprised DNA samples derived from US Caucasian anonymous cord blood donors. The second were controls who completed the online self-administered psychiatric screen and were ascertained as described above, by Knowledge Networks Inc. For the second sample of controls only those without history of schizophrenia, psychosis, BD or major depression with functional impairment were used.

**Sklar, P; Smoller, J | 18711365 [PGC1] | USA (STEP2) | bip_stp2_eur**

The STEP2 sample included BD-1 and BD-2 samples from the STEP-BD study described above along with BD-2 subjects from UCL study also described above. The controls samples for this study were from the NIMH repository as described above for the STEP1 study.

**METHODS**

Individual genotype data for all samples were processed using the PGC “ricopili” pipeline (URLs) for standardized quality control, imputation, and analysis^26^.

------------------------------------------------------------------------------------------

**MAJOR DEPRESSIVE DISORDER (MDD)**

**SAMPLES**

Reference^94^ and PGC MDD (submitted) have full details for the MDD samples. Briefly, this analysis is based on a GWAS mega-analysis of 29 samples of European-ancestry totaling 16,823 MDD cases and 25,632 controls including all samples from the prior PGC MDD report^94^. The majority of these samples passed a structured methodological review by MDD assessment experts (DL and KSK). Cases were required to meet international consensus criteria^95,96,21^ for a lifetime diagnosis of MDD established using structured diagnostic instruments from assessments by trained interviewers, clinician-administered checklists, or medical record review. Most cases were clinically ascertained, and most controls were randomly selected from the population and screened for the absence of lifetime MDD.

**METHODS**

Individual genotype data for all samples were processed using the PGC “ricopili” pipeline (URLs) for standardized quality control, imputation, and analysis^26^.

**Acknowledgements**

The work of Dr Sullivan and the PGC MDD group was supported by NIMH U01 MH109528.

------------------------------------------------------------------------------------------

**OBESSIVE COMPULSIVE DISORDER (OCD)**

**SAMPLES**

All samples are part of the International OCD Foundation Genomics Consortium (IOCDF-GC) or the Obsessive Compulsive Disorder Collaborative Genetics Association Study (OCGAS).

**IOCDF-GC:** Cases included 1,817 individuals and 663 trio probands diagnosed with OCD according to DSM-IV^21^ criteria and recruited predominantly from OCD specialty clinics. Screened controls were recruited from Bonn, Germany and unscreened controls from Capetown, South Africa. For study inclusion, all cases and trio probands were required to have a DSM-IV diagnosis of OCD. The controls from Bonn had an absent lifetime history of all axis I disorders and the South African controls were diagnostically unscreened. This work was approved by the relevant IRBs at all participating sites, and all participants provided written informed consent. Additionally, 5,654 unscreened controls, genotyped on two different Illumina SNP arrays, came from: 1) the Study of Addiction: Genes and Environment (SAGE) cohort (1,288 individuals)^13,97^; 2) the HYPERGENES Consortium Milan, Italy (501 individuals); 3) the Illumina ‘iControl’ Genotype Control Database (3,212 individuals,); and 4) a cohort of Dutch ancestry (653 individuals)^12^.

**OCGAS:** A total of 1 065 families were included in the OCGAS study (comprising 1,406 patients with OCD and 2,895 individuals in total). The sample comprised of 460 complete trios (including an affected proband and both parents); 155 pedigrees with a proband and an unaffected sibling and 450 families with another structure (complex family structure). An additional 192 probands without an additional family member present in the study (singletons) were included. For study inclusion, probands were required to meet DSM-IV criteria for OCD^21^ with onset of obsessions and/or compulsions before the age of 18 years (mean = 9.4 years; SD=6.35). Each case was evaluated by a PhD-level clinical psychologist, using the Structured Clinical Interview for DSM-IV (SCID), modified and extended to include additional symptom and diagnostic information. Final diagnostic status was assigned based on the consensus of two psychiatrists or psychologists reviewing the case independently. To increase the power of the study to detect significant association, we also included 1 1194 screened controls from the Genomic Psychiatry Cohort (GPC). Individuals with a self-reported or diagnosed neuropsychiatric disorder at the time of enrollment were excluded from the present study.

**METHODS**

**Genotyping and QC (IOCDFGC):** Subjects were genotyped on a combination of platforms including the Illumina Human610-Quadv1_B SNP array, the Illumina Hap1M, and the Illumina Hap550k_v1. Platform-specific QC includes removing SNPs and samples with low call rate (<98%), samples with ambiguous genomic sex or discordance between genomic and phenotypic sex, monomorphic SNPs and copy number variation targeted SNPs, SNPs with MAF<0.01, and strand-ambiguous SNPs or SNPs with allele frequency significantly different from HapMap CEU reference data. The batch effect was investigated on the samples genotyped at CGA, and no evidence for batch effect was found. Three SNPs with p<10^-5^ in the batch effect regression analysis were flagged, and none of these appeared in the top 580 SNPs in the case-control meta-analysis or in the top 584 SNPs in the final case-control and trio meta-analysis. Any SNPs detected with low concordance rate among different platforms were removed from OCD GWAS dataset. At this stage in the QC process, all samples were merged into a single dataset using PLINK. Platforms were merged after platform-specific cleaning was performed and 23 SNPs were either mismatched or tri-allelic and were removed. SNP allele frequencies in controls were compared among each platform and any SNP with an absolute allele frequency difference >0.15 between two platforms were flagged. SNPs with difference of missing rate in cases versus controls >0.02 were excluded. SNPs with Mendelian error rate >1% among 400 complete trios (IOTR) were removed. Multidimensional scaling (MDS) analyses were performed on cases, controls, and trio probands, and non-European descent samples and trios and population outliers were removed. Remaining European descent cases and controls were separated into three strata (IOEU for European, IOAJ for Ashkenazi Jewish, and IOSA for South African) based on observed genetic ancestry and source population. Within each of the subpopulations, samples with extreme inbreeding coefficient |F|>0.05 were removed. SNPs with Hardy Weinberg Equilibrium (HWE) p<10^-10^ in controls or p<10^-6^ in cases were removed, and those with HWE p<10^-5^ in controls were flagged. Lastly, any SNP not in common between the cleaned Hap1M, Hap610 and Hap550 platforms were removed, leaving 463,924, 456,734, 548,732, and 557,624 cleaned SNPs in IOEU, IOAJ, IOSA, and IOTR subpopulation, respectively, for subsequent analyses.

**Genotyping and QC (OCGAS):** Genotyping of recruited samples was performed at the Johns Hopkins SNP Center using Illumina’s HumanOmniExpress bead chips (Illumina Inc., San Diego, CA, USA). GPC controls were genotyped at USC using Illumina HumanOmniExpress 12 v 1.0 bead chips. Pedigree samples were converted to independent trios and unrelated cases. The GPC controls were used to match the cases based on MDS analysis. Quality control was applied in trios (OCTR) and case/control cohort (OCCC) separately, including removing SNPs and samples with call rate <98%, samples with ambiguous genomic sex or discordance between genomic and phenotypic sex, samples with discordance between genetic relationship and relationship on the clinical record, monomorphic SNPs and copy number variation targeted SNPs, strand-ambiguous SNPs, and SNPs with MAF<0.01. SNPs with HWE p<10^-10^ in controls or p<10^-6^ in cases were removed. MDS was carried out on case/control cohort and trio probands, and non-European ancestry samples and population outliers were removed. In case/control cohort, SNPs with difference of missing rate in cases versus controls >0.02 were excluded, leaving 545,630 cleaned SNPs for subsequent analyses. In trios, samples with Mendelian errors >10,000 and SNPs with Mendelian errors >4 were removed, leaving 581,862 cleaned SNPs for subsequent analyses.

Genotyped data of IOCDF-GC, OCGAS, and GPC samples were merged into one data set, SNPs with different strand orientation were flipped using 1000 Genomes Project (Phase I integrated variant set release; NCBI build 37 (hg19) as the reference. Pair-wise identity-by-descent (IBD) matrix was generated on all samples using linkage disequilibrium pruned (r2<0.15) SNPs, and overlapped or related samples (pi_hat>0.2) between any two data sets were identified, and one of each duplicate/relative pairs were removed so that only independent samples and trios were kept for GWAS and meta-analysis. The final analysis dataset comprise of 1,429 cases, 5089 controls, and 285 trios from IOCDF-GC, 344 cases and 630 trios from OCGAS, and 1033 GPC samples matching for OCGAS cases.

**GWAS and meta-analysis:** GWAS genotype data from the IOCDF-GC (except the Dutch samples, which were imputed separately, see below), OCGAS and GPC samples using IMPUTE2^56^ and reference haplotypes from the 1000 Genomes Project (Phase I integrated variant set release; NCBI build 37 (hg19) were constructed with SHAPEIT2^28^. SNPs were excluded if IMPUTE2 info was <0.6, IMPUTE2 certainty was <0.9, or MAF<0.01. Separate genome-wide association analyses were conducted for each case-control subpopulation (IOCDF-GC European (IOEU), IOCDF-GC Ashkenazi Jewish (IOAJ), IOCDF-GC South African (IOSA), OCGAS case-control (OCCC)) and trio sample (IOCDF-GC trios (IOTR) and OCGAS trios (OCTR); as probands versus pseudo-controls) using PLINK to perform logistic association correcting for the top 10 principal components. Due to more stringent data sharing restrictions for Dutch cases, imputation and GWAS for the Dutch cases and population-matched controls (IODU) were calculated separately by the site investigators, following the same imputation and quality control procedures. An inverse variance meta-analysis was then performed using the summary statistics of all case-control subpopulations (including IODU) and trio samples using the software package METAL^30^.

**Heritability Analysis:** The LD score regression (LDSC) method^50^ was applied to 1,159,580 imputed and directly genotyped SNPs included in the OCD meta-analysis that also overlapped with a panel of high confidence HapMap3 SNPs. Regression weights were calculated using the HapMap European reference sample provided by Bulik-Sullivan and colleagues. Heritability was then calculated and the datasetchecked for residual population stratification (based on the LDSC intercept), followed by calculation of the genetic correlation between the two consortium sample collections. All 10,215 (2,936 cases and 7,279 controls) individuals in the GWAS meta-analysis comprised the sample for heritability estimation and the test for population stratification. To transform from the observed heritability scale to the liability scale, a population prevalence of 2.5% was used.

**Acknowledgements**

We acknowledge the patients and their families who participated in these studies, and the clinicians and investigators who collaborated on the data collection and analysis of the parent studies. We also acknowledge the International OCD Foundation, the Tourette Association of America, and the Judah Foundation for helping to fund the initial genome-wide association studies. The OCD Collaborative Genetics Association Study (OCGAS) is a collaborative research study including investigators at six sites in the United States and was funded by NIMH Grants R01MH071507, R01 MH079494, R01 MH079487, R01 MH079489, and R01 MH079494. The study includes the following collaborative centers: Johns Hopkins University [Gerald Nestadt]; Brown University/Butler Hospital [Benjamin D. Greenberg]; Columbia University, [Abby Fyer]; Harvard/Massachusetts General Hospital [Dan Geller]; National Institute of Mental Health (NIMH) [Dennis Murphy]; University of California at Los Angeles (UCLA) [James McCracken]; and University of Southern California [James Knowles]. We are indebted to the participating families for their contribution of time and effort in support of this study.

------------------------------------------------------------------------------------------

**SCHIZOPHRENIA (SZC)**

**SAMPLES**

Case sample:

Samples from treatment-resistant schizophrenia (TRS) patients were collected in the UK through the blood-monitoring system used by consumers of the antipsychotic clozapine. The CLOZUK1 sample was initially assembled in collaboration with Novartis (Basel, Switzerland). The company, through their proprietary Clozaril® Patient Monitoring Service (CPMS), provided whole-blood samples and anonymised phenotypic information for 6,882 clozapine-takers. In parallel, the CLOZUK2 sample was assembled in collaboration with Leyden Delta (Nijmegen, Netherlands). The company, through their proprietary Zaponex® Treatment Access System (ZTAS), provided whole-blood samples and anonymised phenotypic information for 7,417 clozapine-takers. Both Clozaril® and Zaponex® are bioequivalent brands of clozapine licensed for marketing in the UK^98^. As both samples are intrinsically related, the term “CLOZUK” will be used throughout this manuscript to describe them together. As stated in guidelines by the UK National Institute for Clinical Excellence (NICE), prescription of clozapine is only initiated in schizophrenia cases after therapeutic failure of two trials of common antipsychotics. This strict criteria ensures that patients sampled for CLOZUK conform to a standardised description of TRS^99^, consistent to that used in other initiatives to elucidate its genetic architecture ^100101102^.

Additionally, a cohort of patients with schizophrenia recruited in the UK (CardiffCOGS) was also included as part of the study. This cohort it’s a conventional sample of those with schizophrenia recruited via secondary care, mainly outpatient, mental health services in Wales and England. Thus, it includes patients that were not taking clozapine at the moment of their recruitment, and do not conform to a TRS classification. However, the recruitment procedure assured that all of its members have been specifically screened for neuropsychiatric disorders using standardized approaches and case-note reviews, as previously reported^103^.

Genotyping for the cases was performed by the Broad Institute (Massachusetts, USA) for the CLOZUK1 sample and 512 CardiffCOGS cases, using OmniExpress-12 and OmniExpressExome-8 chips as described elsewhere^103^. The remaining 247 CardiffCOGS cases and the CLOZUK2 sample were genotyped by deCODE Genetics (Reykjavík, Iceland), using OmniExpress-12 chips.

Control sample:

Control samples were collected from publicly available sources (EGA) or through collaboration with the dataset holders. Individual datasets were curated with the same specifications as the case-only datasets. In order to maximize the numbers of individuals that could be effectively included in the GWAS without introducing confounders, these datasets were chosen in the basis of having recruited individuals with self-reported British ancestry (either exclusively or primarily) and having been genotyped on Illumina chips.

**METHODS**

**QC:** Given the many data sources used and the variety of genotyping chips available, a stringent quality control (allowing only 2% of missing SNP and individual data) was performed separately in each individual dataset, using PLINK v1.9^47^ and following standard procedures^104^. To facilitate merging and to avoid common sources of batch effects^105^, all SNPs in each dataset were also aligned to the plus strand of the human genome (build 37p13), removing strand-ambiguous markers in the process. As most control datasets lacked any markers in the X and Y-chromosomes, or in the mitochondrial DNA, every SNP from these regions was discarded. The final merge of all case and control datasets left 203,436 overlapping autosomal SNPs.

All individuals were imputed simultaneously in the Cardiff University high-performance computing cluster RAVEN^106^, using the SHAPEIT/IMPUTE2 algorithms ^5628^. As reference panels, a combination of the 1000 Genomes phase 3 (1KGPp3) and UK10K datasets was used, as this has previously been shown to increase the accuracy of imputation for individuals of British ancestry, particularly for rare variants^107^. After imputation, a principal component analysis (PCA) of variants with minor allele frequency (MAF) higher than 5% was carried out to obtain a general summary of the population structure of the sample, using the EIGENSOFT v6 toolset^108^. In order to ameliorate population stratification in the association analysis^109^, any individuals not falling into an area delimited by the mean and 3 standard deviations of the two first principal components of the control samples were excluded from further analyses. The sample was further pruned by removing all individuals with inbreeding coefficients (F) higher than 0.2, and leaving only a random member of each pair with a relatedness coefficient ($\hat{\pi}$) higher than 0.2. To ensure the independence of our analyses with previous GWASes conducted by the Schizophrenia Working Group of the Psychiatric Genomics Consortium (SCZ-PGC), relatedness coefficients of CLOZUK individuals were also calculated with all the individual datasets included in the latest PGC study (PGC2)^26^. Detected genetic relatives were excluded as aforementioned. After this imputation and curation process, 35802 samples (11260 cases + 24542 controls) with 9.65 million imputed markers (INFO > 0.3 and MAF > 0.001) remained in the dataset.

**PGC2 meta-analysis:** The CLOZUK GWAS results were combined with a version of PGC2 in which the CLOZUK1 sample had been previously removed. Meta-analysis was performed by using the fixed-effects procedure in METAL^30^ and weights derived from standard errors. For consistency with the PGC2 mega-analysis, additional filters (INFO > 0.6 and MAF > 0.01) were applied to the CLOZUK and PGC2noCLOZUK summary statistics, leaving 8 million markers in the final meta-analysis results. The same procedure as above was used in order to report independent loci from this analysis. As raw SCZ-PGC2 genotypes were not available for using in the LD-clumping procedure, 1KGPp3 was used as a reference.

**Heritability Analysis:** We used the software LD-Score v1.0^50^ to analyse the aforementioned summary statistics, and obtain an estimate of SNP-based heritability (h^2^), in the CLOZUK+PGC2 meta-analysis, the largest combined sample we had available (40,675 cases and 64,643 controls). Heritability estimates were transformed to the liability scale using a prevalence of 0.1%, as estimated from population registry studies^110^. As population structure and sample overlap were explicitly accounted during the curation process of both the CLOZUK and PGC2 studies, the intercept of the LD-score regression was constrained to 1^111^.

**Acknowledgements**:

Case sample:

We thank the participants and clinicians who took part in the CardiffCOGS study. We acknowledge Sophie Bishop and Amy Lynham, from Cardiff University, for their work in recruitment, interviewing and rating of participants. For the CLOZUK2 sample we thank Leyden Delta, for supporting the sample collection, anonymisation and data preparation (particularly Marinka Helthius, John Jansen and Karel Jollie), Magna Laboratories, UK (Andy Walker) and, for CLOZUK1, Novartis and The Doctor’s Laboratory staff for their guidance and cooperation. We acknowledge Kiran Mantripragada, Lesley Bates, Catherine Bresner and Lucinda Hopkins, at Cardiff University, for laboratory sample management.

Control sample:

A full list of the investigators who contributed to the generation of the Wellcome Trust Case Control Consortium (WTCCC) data is available from www.wtccc.org.uk. Funding for the project was provided by the Wellcome Trust (WT) under award 076113. Venous blood collection for the 1958 Birth Cohort (NCDS) was funded by the UK’s Medical Research Council (MRC) grant G0000934, peripheral blood lymphocyte preparation by Juvenile Diabetes Research Foundation (JDRF) and WT and the cell-line production, DNA extraction and processing by WT grant 06854/Z/02/Z. Genotyping was supported by WT (083270) and the European Union (EU; ENGAGE: HEALTH-F4-2007- 201413). The UK Blood Services Common Controls (UKBS-CC collection) was funded by WT (076113/C/04/Z) and by the National Institute for Health Research (NIHR) programme grant to NHS Blood and Transplant authority (NHSBT; RP-PG-0310-1002). NHSBT also made possible the recruitment of the Cardiff Controls, from participants who provided informed consent. Generation Scotland received core funding from the Chief Scientist Office of the Scottish Government Health Directorates CZD/16/6 and the Scottish Funding Council HR03006. Genotyping of the GS:SFHS samples was carried out by the Genetics Core Laboratory at the WT Clinical Research Facility, Edinburgh, Scotland and was funded by the MRC. The Type 1 Diabetes Genetics Consortium (T1DGC) is a collaborative clinical study sponsored by the National Institute of Diabetes and Digestive and Kidney Diseases (NIDDK), National Institute of Allergy and Infectious Diseases (NIAID), National Human Genome Research Institute (NHGRI), National Institute of Child Health and Human Development (NICHD), and JDRF. The People of the British Isles (POBI) project is supported by WT (072974/Z/03/Z, 088262/Z/09/Z, 075491/Z/04/Z, 075491/Z/04/A, 075491/Z/04/B, 090532/Z/09/Z, 084818/Z/08/Z, 095552/Z/11/Z, 085475/Z/08/Z, 098387/Z/12/Z, 098386/Z/12/Z), the Academy of Finland (257654) and the Australian National Health and Medical Research Council (APP1053756). TwinsUK is funded by WT, MRC, EU, NIHR-funded BioResource, Clinical Research Facility and Biomedical Research Centre based at Guy’s and St Thomas’ NHS Foundation Trust in partnership with King’s College London. Funding for the QIMR samples was provided by the Australian National Health and Medical Research Council (241944, 339462, 389875, 389891, 389892, 389927, 389938, 442915, 442981, 496675, 496739, 552485, 552498, 613602, 613608, 613674, 619667), the Australian Research Council (FT0991360, FT0991022), the FP-5 GenomEUtwin Project (QLG2-CT- 2002-01254) and the US National Institutes of Health (NIH; AA07535, AA10248, AA13320, AA13321, AA13326, AA14041, MH66206, DA12854, DA019951), and the Center for Inherited Disease Research (Baltimore, MD, USA). TEDS is supported by a program grant from the MRC [G0901245-G0500079], with additional support from the NIH [HD044454; HD059215]. In the GERAD Consortium, Cardiff University was supported by WT, MRC, Alzheimer’s Research UK (ARUK) and the Welsh Government. Kings College London acknowledges support from the MRC. The University of Belfast acknowledges support from ARUK, Alzheimer's Society, Ulster Garden Villages, N.Ireland R&D Office and the Royal College of Physicians/Dunhill Medical Trust. Washington University was funded by NIH grants, Barnes Jewish Foundation and the Charles and Joanne Knight Alzheimer's Research Initiative. The Bonn group was supported by the German Federal Ministry of Education and Research (BMBF), Competence Network Dementia and Competence Network Degenerative Dementia, and by the Alfried Krupp von Bohlen und Halbach-Stiftung.

General:

This project has received funding from the European Union’s Seventh Framework Programme for research, technological development and demonstration under grant agreement n° 279227. The work at Cardiff University was funded by Medical Research Council (MRC) Centre (MR/L010305/1) and Program Grants (G0800509) and the European Community’s Seventh Framework Programme (HEALTH-F2-2010-241909 (Project EU-GEI). M.C.O’D., M.J.O. and G.K. have received funding from the MRC and the Wellcome Trust, UK. This work was supported by a clinical research fellowship to J.T.R.W. from the MRC/Welsh Assembly Government and the Margaret Temple Award from the British Medical Association.

**REFERENCES**

1 Adkins DE, Clark SL, Copeland WE, Kennedy M, Conway K, Angold A *et al.* Genome-Wide Meta-Analysis of Longitudinal Alcohol Consumption Across Youth and Early Adulthood. *Twin Res Hum Genet Off J Int Soc Twin Stud* 2015; **18**: 335–347.

2 Costello EJ, Eaves L, Sullivan P, Kennedy M, Conway K, Adkins DE *et al.* Genes, environments, and developmental research: methods for a multi-site study of early substance abuse. *Twin Res Hum Genet Off J Int Soc Twin Stud* 2013; **16**: 505–515.

3 Treutlein J, Cichon S, Ridinger M, Wodarz N, Soyka M, Zill P *et al.* Genome-wide association study of alcohol dependence. *Arch Gen Psychiatry* 2009; **66**: 773–784.

4 Frank J, Cichon S, Treutlein J, Ridinger M, Mattheisen M, Hoffmann P *et al.* Genome-wide significant association between alcohol dependence and a variant in the ADH gene cluster. *Addict Biol* 2012; **17**: 171–180.

5 Fergusson DM, Horwood LJ. The Christchurch Health and Development Study. In: Joyce P, Nicholls G, Thomas K, Wilkinson T (eds). *The Christchurch Experience: 40 Years of Research and Teaching*. University of Otago: Christchurch, 2013, pp 79–87.

6 Pierucci-Lagha A, Gelernter J, Chan G, Arias A, Cubells JF, Farrer L *et al.* Reliability of DSM-IV diagnostic criteria using the semi-structured assessment for drug dependence and alcoholism (SSADDA). *Drug Alcohol Depend* 2007; **91**: 85–90.

7 Pierucci-Lagha A, Gelernter J, Feinn R, Cubells JF, Pearson D, Pollastri A *et al.* Diagnostic reliability of the Semi-structured Assessment for Drug Dependence and Alcoholism (SSADDA). *Drug Alcohol Depend* 2005; **80**: 303–312.

8 Gelernter J, Kranzler HR, Sherva R, Almasy L, Koesterer R, Smith AH *et al.* Genome-wide association study of alcohol dependence:significant findings in African- and European-Americans including novel risk loci. *Mol Psychiatry* 2014; **19**: 41–49.

9 Edenberg HJ, Koller DL, Xuei X, Wetherill L, McClintick JN, Almasy L *et al.* Genome-wide association study of alcohol dependence implicates a region on chromosome 11. *Alcohol Clin Exp Res* 2010; **34**: 840–852.

10 Bucholz KK, Hesselbrock VM, Shayka JJ, Nurnberger JI, Schuckit MA, Schmidt I *et al.* Reliability of individual diagnostic criterion items for psychoactive substance dependence and the impact on diagnosis. *J Stud Alcohol* 1995; **56**: 500–505.

11 Reich T, Edenberg HJ, Goate A, Williams JT, Rice JP, Van Eerdewegh P *et al.* Genome-wide search for genes affecting the risk for alcohol dependence. *Am J Med Genet* 1998; **81**: 207–215.

12 Bierut LJ, Strickland JR, Thompson JR, Afful SE, Cottler LB. Drug use and dependence in cocaine dependent subjects, community-based individuals, and their siblings. *Drug Alcohol Depend* 2008; **95**: 14–22.

13 Bierut LJ, Madden PAF, Breslau N, Johnson EO, Hatsukami D, Pomerleau OF *et al.* Novel genes identified in a high-density genome wide association study for nicotine dependence. *Hum Mol Genet* 2007; **16**: 24–35.

14 Bierut LJ, Agrawal A, Bucholz KK, Doheny KF, Laurie C, Pugh E *et al.* A genome-wide association study of alcohol dependence. *Proc Natl Acad Sci U S A* 2010; **107**: 5082–5087.

15 Nelson EC, Agrawal A, Heath AC, Bogdan R, Sherva R, Zhang B *et al.* Evidence of CNIH3 involvement in opioid dependence. *Mol Psychiatry* 2016; **21**: 608–614.

16 Hartman CA, Gelhorn H, Crowley TJ, Sakai JT, Stallings M, Young SE *et al.* Item response theory analysis of DSM-IV cannabis abuse and dependence criteria in adolescents. *J Am Acad Child Adolesc Psychiatry* 2008; **47**: 165–173.

17 Petrill S, Plomin R, DeFries JC, Hewitt JK (eds.). *Nature, nurture, and the transition to adolescence*. Oxford University Press: New York, 2003.

18 Rhea S-A, Gross AA, Haberstick BC, Corley RP. Colorado Twin Registry. *Twin Res Hum Genet Off J Int Soc Twin Stud* 2006; **9**: 941–949.

19 Stallings MC, Corley RP, Dennehey B, Hewitt JK, Krauter KS, Lessem JM *et al.* A genome-wide search for quantitative trait Loci that influence antisocial drug dependence in adolescence. *Arch Gen Psychiatry* 2005; **62**: 1042–1051.

20 1000 Genomes Project Consortium, Auton A, Brooks LD, Durbin RM, Garrison EP, Kang HM *et al.* A global reference for human genetic variation. *Nature* 2015; **526**: 68–74.

21 American Psychiatric Association. *Diagnostic and Statistical Manual of mental disorders, 4th*. 4th ed. APA: Washington DC, 1994.

22 Wang K, Zhang H, Bloss CS, Duvvuri V, Kaye W, Schork NJ *et al.* A genome-wide association study on common SNPs and rare CNVs in anorexia nervosa. *Mol Psychiatry* 2011; **16**: 949–959.

23 Boraska V, Franklin CS, Floyd J a. B, Thornton LM, Huckins LM, Southam L *et al.* A genome-wide association study of anorexia nervosa. *Mol Psychiatry* 2014; **19**: 1085–1094.

24 Purcell S, Neale B, Todd-Brown K, Thomas L, Ferreira MAR, Bender D *et al.* PLINK: a tool set for whole-genome association and population-based linkage analyses. *Am J Hum Genet* 2007; **81**: 559–575.

25 Galinsky KJ, Bhatia G, Loh P-R, Georgiev S, Mukherjee S, Patterson NJ *et al.* Fast Principal-Component Analysis Reveals Convergent Evolution of ADH1B in Europe and East Asia. *Am J Hum Genet* 2016; **98**: 456–472.

26 Schizophrenia Working Group of the Psychiatric Genomics Consortium. Biological insights from 108 schizophrenia-associated genetic loci. *Nature* 2014; **511**: 421–427.

27 1000 Genomes Project Consortium, Abecasis GR, Altshuler D, Auton A, Brooks LD, Durbin RM *et al.* A map of human genome variation from population-scale sequencing. *Nature* 2010; **467**: 1061–1073.

28 Delaneau O, Marchini J, Zagury J-F. A linear complexity phasing method for thousands of genomes. *Nat Methods* 2011; **9**: 179–181.

29 Howie BN, Donnelly P, Marchini J. A flexible and accurate genotype imputation method for the next generation of genome-wide association studies. *PLoS Genet* 2009; **5**: e1000529.

30 Willer CJ, Li Y, Abecasis GR. METAL: fast and efficient meta-analysis of genomewide association scans. *Bioinforma Oxf Engl* 2010; **26**: 2190–2191.

31 Mors O, Perto GP, Mortensen PB. The Danish Psychiatric Central Research Register. *Scand J Public Health* 2011; **39**: 54–57.

32 Elia J, Gai X, Xie HM, Perin JC, Geiger E, Glessner JT *et al.* Rare structural variants found in attention-deficit hyperactivity disorder are preferentially associated with neurodevelopmental genes. *Mol Psychiatry* 2010; **15**: 637–646.

33 Neale BM, Lasky-Su J, Anney R, Franke B, Zhou K, Maller JB *et al.* Genome-wide association scan of attention deficit hyperactivity disorder. *Am J Med Genet Part B Neuropsychiatr Genet Off Publ Int Soc Psychiatr Genet* 2008; **147B**: 1337–1344.

34 Neale BM, Medland SE, Ripke S, Asherson P, Franke B, Lesch K-P *et al.* Meta-analysis of genome-wide association studies of attention-deficit/hyperactivity disorder. *J Am Acad Child Adolesc Psychiatry* 2010; **49**: 884–897.

35 Mick E, Todorov A, Smalley S, Hu X, Loo S, Todd RD *et al.* Family-based genome-wide association scan of attention-deficit/hyperactivity disorder. *J Am Acad Child Adolesc Psychiatry* 2010; **49**: 898–905.e3.

36 Lionel AC, Crosbie J, Barbosa N, Goodale T, Thiruvahindrapuram B, Rickaby J *et al.* Rare copy number variation discovery and cross-disorder comparisons identify risk genes for ADHD. *Sci Transl Med* 2011; **3**: 95ra75.

37 Sánchez-Mora C, Ramos-Quiroga JA, Bosch R, Corrales M, Garcia-Martínez I, Nogueira M *et al.* Case-control genome-wide association study of persistent attention-deficit hyperactivity disorder identifies FBXO33 as a novel susceptibility gene for the disorder. *Neuropsychopharmacol Off Publ Am Coll Neuropsychopharmacol* 2015; **40**: 915–926.

38 Zayats T, Athanasiu L, Sonderby I, Djurovic S, Westlye LT, Tamnes CK *et al.* Genome-wide analysis of attention deficit hyperactivity disorder in Norway. *PloS One* 2015; **10**: e0122501.

39 Stergiakouli E, Hamshere M, Holmans P, Langley K, Zaharieva I, deCODE Genetics *et al.* Investigating the contribution of common genetic variants to the risk and pathogenesis of ADHD. *Am J Psychiatry* 2012; **169**: 186–194.

40 Hinney A, Scherag A, Jarick I, Albayrak Ö, Pütter C, Pechlivanis S *et al.* Genome-wide association study in German patients with attention deficit/hyperactivity disorder. *Am J Med Genet Part B Neuropsychiatr Genet Off Publ Int Soc Psychiatr Genet* 2011; **156B**: 888–897.

41 Neale BM, Medland S, Ripke S, Anney RJL, Asherson P, Buitelaar J *et al.* Case-control genome-wide association study of attention-deficit/hyperactivity disorder. *J Am Acad Child Adolesc Psychiatry* 2010; **49**: 906–920.

42 O’Donovan MC, Craddock N, Norton N, Williams H, Peirce T, Moskvina V *et al.* Identification of loci associated with schizophrenia by genome-wide association and follow-up. *Nat Genet* 2008; **40**: 1053–1055.

43 Gelernter J, Sherva R, Koesterer R, Almasy L, Zhao H, Kranzler HR *et al.* Genome-wide association study of cocaine dependence and related traits: FAM53B identified as a risk gene. *Mol Psychiatry* 2014; **19**: 717–723.

44 Gelernter J, Kranzler HR, Sherva R, Koesterer R, Almasy L, Zhao H *et al.* Genome-wide association study of opioid dependence: multiple associations mapped to calcium and potassium pathways. *Biol Psychiatry* 2014; **76**: 66–74.

45 Howie B, Marchini J, Stephens M. Genotype imputation with thousands of genomes. *G3 Bethesda Md* 2011; **1**: 457–470.

46 Sudmant PH, Rausch T, Gardner EJ, Handsaker RE, Abyzov A, Huddleston J *et al.* An integrated map of structural variation in 2,504 human genomes. *Nature* 2015; **526**: 75–81.

47 Chang CC, Chow CC, Tellier LC, Vattikuti S, Purcell SM, Lee JJ. Second-generation PLINK: rising to the challenge of larger and richer datasets. *GigaScience* 2015; **4**: 7.

48 Price AL, Patterson NJ, Plenge RM, Weinblatt ME, Shadick NA, Reich D. Principal components analysis corrects for stratification in genome-wide association studies. *Nat Genet* 2006; **38**: 904–909.

49 Winkler TW, Day FR, Croteau-Chonka DC, Wood AR, Locke AE, Mägi R *et al.* Quality control and conduct of genome-wide association meta-analyses. *Nat Protoc* 2014; **9**: 1192–1212.

50 Bulik-Sullivan BK, Loh P-R, Finucane HK, Ripke S, Yang J, Schizophrenia Working Group of the Psychiatric Genomics Consortium *et al.* LD Score regression distinguishes confounding from polygenicity in genome-wide association studies. *Nat Genet* 2015; **47**: 291–295.

51 Polanczyk G, de Lima MS, Horta BL, Biederman J, Rohde LA. The worldwide prevalence of ADHD: a systematic review and metaregression analysis. *Am J Psychiatry* 2007; **164**: 942–948.

52 Anney R, Klei L, Pinto D, Regan R, Conroy J, Magalhaes TR *et al.* A genome-wide scan for common alleles affecting risk for autism. *Hum Mol Genet* 2010; **19**: 4072–4082.

53 Lajonchere CM, AGRE Consortium. Changing the landscape of autism research: the autism genetic resource exchange. *Neuron* 2010; **68**: 187–191.

54 Gauthier J, Joober R, Dubé M-P, St-Onge J, Bonnel A, Gariépy D *et al.* Autism spectrum disorders associated with X chromosome markers in French-Canadian males. *Mol Psychiatry* 2006; **11**: 206–213.

55 Fischbach GD, Lord C. The Simons Simplex Collection: a resource for identification of autism genetic risk factors. *Neuron* 2010; **68**: 192–195.

56 Howie B, Fuchsberger C, Stephens M, Marchini J, Abecasis GR. Fast and accurate genotype imputation in genome-wide association studies through pre-phasing. *Nat Genet* 2012; **44**: 955–959.

57 1000 Genomes Project Consortium, Abecasis GR, Auton A, Brooks LD, DePristo MA, Durbin RM *et al.* An integrated map of genetic variation from 1,092 human genomes. *Nature* 2012; **491**: 56–65.

58 Price AL, Weale ME, Patterson N, Myers SR, Need AC, Shianna KV *et al.* Long-range LD can confound genome scans in admixed populations. *Am J Hum Genet* 2008; **83**: 132-135-139.

59 Begum F, Ghosh D, Tseng GC, Feingold E. Comprehensive literature review and statistical considerations for GWAS meta-analysis. *Nucleic Acids Res* 2012; **40**: 3777–3784.

60 International HapMap 3 Consortium, Altshuler DM, Gibbs RA, Peltonen L, Altshuler DM, Gibbs RA *et al.* Integrating common and rare genetic variation in diverse human populations. *Nature* 2010; **467**: 52–58.

61 Hansen SN, Overgaard M, Andersen PK, Parner ET. Estimating a population cumulative incidence under calendar time trends. *BMC Med Res Methodol* 2017; **17**: 7.

62 Psychiatric GWAS Consortium Bipolar Disorder Working Group. Large-scale genome-wide association analysis of bipolar disorder identifies a new susceptibility locus near ODZ4. *Nat Genet* 2011; **43**: 977–983.

63 Kendler KS, McGuire M, Gruenberg AM, O’Hare A, Spellman M, Walsh D. The Roscommon Family Study. I. Methods, diagnosis of probands, and risk of schizophrenia in relatives. *Arch Gen Psychiatry* 1993; **50**: 527–540.

64 Faraone SV, Blehar M, Pepple J, Moldin SO, Norton J, Nurnberger JI *et al.* Diagnostic accuracy and confusability analyses: an application to the Diagnostic Interview for Genetic Studies. *Psychol Med* 1996; **26**: 401–410.

65 Sheehan DV, Lecrubier Y, Sheehan KH, Amorim P, Janavs J, Weiller E *et al.* The Mini-International Neuropsychiatric Interview (M.I.N.I.): the development and validation of a structured diagnostic psychiatric interview for DSM-IV and ICD-10. *J Clin Psychiatry* 1998; **59 Suppl 20**: 22-33-57.

66 Nurnberger JI, Blehar MC, Kaufmann CA, York-Cooler C, Simpson SG, Harkavy-Friedman J *et al.* Diagnostic interview for genetic studies. Rationale, unique features, and training. NIMH Genetics Initiative. *Arch Gen Psychiatry* 1994; **51**: 849-859-864.

67 Maxwell M. *Family interview for genetic studies.* Clinical Neurogenetics Branch, Intramural Research Program, 1992.

68 Wing JK, Babor T, Brugha T, Burke J, Cooper JE, Giel R *et al.* SCAN. Schedules for Clinical Assessment in Neuropsychiatry. *Arch Gen Psychiatry* 1990; **47**: 589–593.

69 Nilsson L-G, Backman L, Erngrund K, Nyberg L, Adolfsson R, Bucht G *et al.* The betula prospective cohort study: Memory, health, and aging. *Aging Neuropsychol Cogn* 1997; **4**: 1–32.

70 Spitzer RL, Endicott J. *The Schedule for Affective Disorder and Schizophrenia, Lifetime Version*. New York State Psychiatric Institute: New York.

71 Spitzer RL, Endicott J, Robins E. Research diagnostic criteria: rationale and reliability. *Arch Gen Psychiatry* 1978; **35**: 773–782.

72 O’Dushlaine C, Ripke S, Ruderfer DM, Hamilton SP, Fava M, Iosifescu DV *et al.* Rare copy number variation in treatment-resistant major depressive disorder. *Biol Psychiatry* 2014; **76**: 536–541.

73 Spitzer RL, Williams JB, Gibbon M, First MB. The Structured Clinical Interview for DSM-III-R (SCID). I: History, rationale, and description. *Arch Gen Psychiatry* 1992; **49**: 624–629.

74 Spitzer RL, Williams JB, Kroenke K, Linzer M, deGruy FV, Hahn SR *et al.* Utility of a new procedure for diagnosing mental disorders in primary care. The PRIME-MD 1000 study. *JAMA* 1994; **272**: 1749–1756.

75 Frye MA, McElroy SL, Fuentes M, Sutor B, Schak KM, Galardy CW *et al.* Development of a bipolar disorder biobank: differential phenotyping for subsequent biomarker analyses. *Int J Bipolar Disord* 2015; **3**: 30.

76 Olson JE, Ryu E, Johnson KJ, Koenig BA, Maschke KJ, Morrisette JA *et al.* The Mayo Clinic Biobank: a building block for individualized medicine. *Mayo Clin Proc* 2013; **88**: 952–962.

77 Leckman JF, Sholomskas D, Thompson WD, Belanger A, Weissman MM. Best estimate of lifetime psychiatric diagnosis: a methodological study. *Arch Gen Psychiatry* 1982; **39**: 879–883.

78 McKay JD, Truong T, Gaborieau V, Chabrier A, Chuang S-C, Byrnes G *et al.* A genome-wide association study of upper aerodigestive tract cancers conducted within the INHANCE consortium. *PLoS Genet* 2011; **7**: e1001333.

79 Medland SE, Nyholt DR, Painter JN, McEvoy BP, McRae AF, Zhu G *et al.* Common variants in the trichohyalin gene are associated with straight hair in Europeans. *Am J Hum Genet* 2009; **85**: 750–755.

80 Sachs GS. Use of clonazepam for bipolar affective disorder. *J Clin Psychiatry* 1990; **51 Suppl**: 31-34-53.

81 Haas M, Delbello MP, Pandina G, Kushner S, Van Hove I, Augustyns I *et al.* Risperidone for the treatment of acute mania in children and adolescents with bipolar disorder: a randomized, double-blind, placebo-controlled study. *Bipolar Disord* 2009; **11**: 687–700.

82 Hirschfeld RMA, Keck PE, Kramer M, Karcher K, Canuso C, Eerdekens M *et al.* Rapid antimanic effect of risperidone monotherapy: a 3-week multicenter, double-blind, placebo-controlled trial. *Am J Psychiatry* 2004; **161**: 1057–1065.

83 Smulevich AB, Khanna S, Eerdekens M, Karcher K, Kramer M, Grossman F. Acute and continuation risperidone monotherapy in bipolar mania: a 3-week placebo-controlled trial followed by a 9-week double-blind trial of risperidone and haloperidol. *Eur Neuropsychopharmacol J Eur Coll Neuropsychopharmacol* 2005; **15**: 75–84.

84 Berwaerts J, Lane R, Nuamah IF, Lim P, Remmerie B, Hough DW. Paliperidone extended-release as adjunctive therapy to lithium or valproate in the treatment of acute mania: a randomized, placebo-controlled study. *J Affect Disord* 2011; **129**: 252–260.

85 Berwaerts J, Xu H, Nuamah I, Lim P, Hough D. Evaluation of the efficacy and safety of paliperidone extended-release in the treatment of acute mania: a randomized, double-blind, dose-response study. *J Affect Disord* 2012; **136**: e51-60.

86 Vieta E, Nuamah IF, Lim P, Yuen EC, Palumbo JM, Hough DW *et al.* A randomized, placebo- and active-controlled study of paliperidone extended release for the treatment of acute manic and mixed episodes of bipolar I disorder. *Bipolar Disord* 2010; **12**: 230–243.

87 Kaufman J, Birmaher B, Brent D, Rao U, Flynn C, Moreci P *et al.* Schedule for Affective Disorders and Schizophrenia for School-Age Children-Present and Lifetime Version (K-SADS-PL): initial reliability and validity data. *J Am Acad Child Adolesc Psychiatry* 1997; **36**: 980–988.

88 Vreeker A, Boks MPM, Abramovic L, Verkooijen S, van Bergen AH, Hillegers MHJ *et al.* High educational performance is a distinctive feature of bipolar disorder: a study on cognition in bipolar disorder, schizophrenia patients, relatives and controls. *Psychol Med* 2016; **46**: 807–818.

89 Pato MT, Sobell JL, Medeiros H, Abbott C, Sklar BM, Buckley PF *et al.* The genomic psychiatry cohort: partners in discovery. *Am J Med Genet Part B Neuropsychiatr Genet Off Publ Int Soc Psychiatr Genet* 2013; **162B**: 306–312.

90 The AMDP System. *The AMDP-System Association of Methodology and Documentation in Psychiatry. Manual For the Assessment and Documentation of Psychopathology*. Springer: Berlin, 1992.

91 McGuffin P, Farmer A, Harvey I. A polydiagnostic application of operational criteria in studies of psychotic illness. Development and reliability of the OPCRIT system. *Arch Gen Psychiatry* 1991; **48**: 764–770.

92 Schmermund A, Möhlenkamp S, Stang A, Grönemeyer D, Seibel R, Hirche H *et al.* Assessment of clinically silent atherosclerotic disease and established and novel risk factors for predicting myocardial infarction and cardiac death in healthy middle-aged subjects: rationale and design of the Heinz Nixdorf RECALL Study. Risk Factors, Evaluation of Coronary Calcium and Lifestyle. *Am Heart J* 2002; **144**: 212–218.

93 Wittchen HU, Hofler M, Gander F, Pfister H, Storz S, Bedirhan U *et al.* Screening for mental disorders: performance of the Composite International Diagnostic - Screener (CID-S). *Int J Methods Psychiatr Res* 1999; **8**: 59–70.

94 Major Depressive Disorder Working Group of the Psychiatric GWAS Consortium, Ripke S, Wray NR, Lewis CM, Hamilton SP, Weissman MM *et al.* A mega-analysis of genome-wide association studies for major depressive disorder. *Mol Psychiatry* 2013; **18**: 497–511.

95 World Health Organization. *International Classification of Diseases*. Geneva, 1978.

96 World Health Organization. *International Classification of Diseases*. Geneva, 1992.

97 Bierut LJ, Saccone NL, Rice JP, Goate A, Foroud T, Edenberg H *et al.* Defining alcohol-related phenotypes in humans. The Collaborative Study on the Genetics of Alcoholism. *Alcohol Res Health J Natl Inst Alcohol Abuse Alcohol* 2002; **26**: 208–213.

98 Couchman L, Morgan PE, Spencer EP, Johnston A, Flanagan RJ. Plasma clozapine and norclozapine in patients prescribed different brands of clozapine (Clozaril, Denzapine, and Zaponex). *Ther Drug Monit* 2010; **32**: 624–627.

99 Gee S, Vergunst F, Howes O, Taylor D. Practitioner attitudes to clozapine initiation. *Acta Psychiatr Scand* 2014; **130**: 16–24.

100 Goldstein JI, Jarskog LF, Hilliard C, Alfirevic A, Duncan L, Fourches D *et al.* Clozapine-induced agranulocytosis is associated with rare HLA-DQB1 and HLA-B alleles. *Nat Commun* 2014; **5**: 4757.

101 Bilic P, Jukic V, Vilibic M, Savic A, Bozina N. Treatment-resistant schizophrenia and DAT and SERT polymorphisms. *Gene* 2014; **543**: 125–132.

102 Frank J, Lang M, Witt SH, Strohmaier J, Rujescu D, Cichon S *et al.* Identification of increased genetic risk scores for schizophrenia in treatment-resistant patients. *Mol Psychiatry* 2015; **20**: 913.

103 Rees E, Kirov G, Sanders A, Walters JTR, Chambert KD, Shi J *et al.* Evidence that duplications of 22q11.2 protect against schizophrenia. *Mol Psychiatry* 2014; **19**: 37–40.

104 Anderson CA, Pettersson FH, Clarke GM, Cardon LR, Morris AP, Zondervan KT. Data quality control in genetic case-control association studies. *Nat Protoc* 2010; **5**: 1564–1573.

105 Zuvich RL, Armstrong LL, Bielinski SJ, Bradford Y, Carlson CS, Crawford DC *et al.* Pitfalls of merging GWAS data: lessons learned in the eMERGE network and quality control procedures to maintain high data quality. *Genet Epidemiol* 2011; **35**: 887–898.

106 *Advanced Research Computing @ Cardiff (ARCCA). Introduction to RAVEN [Online]*. Cardiff University, 2016http://www.cardiff.ac.uk/arcca/services/equipment/ravenintroduction.html.

107 Huang J, Howie B, McCarthy S, Memari Y, Walter K, Min JL *et al.* Improved imputation of low-frequency and rare variants using the UK10K haplotype reference panel. *Nat Commun* 2015; **6**: 8111.

108 Patterson N, Price AL, Reich D. Population structure and eigenanalysis. *PLoS Genet* 2006; **2**: e190.

109 Tian C, Gregersen PK, Seldin MF. Accounting for ancestry: population substructure and genome-wide association studies. *Hum Mol Genet* 2008; **17**: R143-150.

110 Perälä J, Suvisaari J, Saarni SI, Kuoppasalmi K, Isometsä E, Pirkola S *et al.* Lifetime prevalence of psychotic and bipolar I disorders in a general population. *Arch Gen Psychiatry* 2007; **64**: 19–28.

111 Bulik-Sullivan B, Finucane HK, Anttila V, Gusev A, Day FR, Loh P-R *et al.* An atlas of genetic correlations across human diseases and traits. *Nat Genet* 2015; **47**: 1236–1241.

**COLLABORATING AUTHOR NAMES AND AFFILIATIONS**

**ALCOHOL DEPENDENCE (AD)**

Daniel E Adkins^a^, Arpana Agrawal^b^, Laura J Bierut^b^, Sandra Brown^q,r^, Kathleen Bucholz^b^, Li-Shiun Chen^b^, William E Copeland^g^, Elizabeth Costello^g^, Louisa Degenhardt, Howard J. Edenberg^t,v^, Lindsay Farrer, Tatiana Foroud^v^, Josef Frank^c^, Joel Gelernter^w,x,y^, Ina Giegling^d^, Alison Goate^p^, Annette M Hartmann^d^, Sarah Hartz^b^, Victor Hesselbrock^z^, John Hewitt^j^, Christian Hopfer^a/a^, Horwood L. John, Eric O Johnson^h^, Martin A Kennedy^i^, Bettina Konte^d^, Henry Kranzler^a/b^, Karl Mann, Nicholas G Martin^a/c,a/d^, Matthew B McQueen^j^, Grant W Montgomery^a/e^, Benjamin Neale^a/f,a/g,a/h^, Elliot C Nelson^b^, Markus M Nöthen^a/i,a/j^, John F Pearson^k^, Roseann E. Peterson^e,f^, Bernice Porjesz, Monika Ridinger^l,m^, Marcella Rietschel^c^, Dan Rujescu^d^, Nancy L Saccone^b^, Michael Soyka^o^, Michael Stallings^j,a/k^, Tamara L Wall^a/l,a/m,a/n,a/o,a/p,a/q^, Norbert Wodarz, Hongyu Zhao, Raymond Walters^a/r, a/s^

^a^University of Utah, Sociology, Psychiatry, Salt Lake City, UT, USA

^b^Washington University in Saint Louis School of Medicine, Department of Psychiatry, Saint Louis, MO, USA

^c^Central Institute of Mental Health, Medical Faculty Mannheim, Heidelberg University, Department of Genetic Epidemiology in Psychiatry, Mannheim, Germany

^d^Martin-Luther-University Halle-Wittenberg, Dept. of Psychiatry, Psychotherapy and Psychosomatics, Halle, Germany

^e^Virginia Commonwealth University, Psychiatry, Richmond, VA, USA

^f^Virginia Commonwealth University, Virginia Institute for Psychiatric and Behavioral Genetics, Richmond, VA, USA

^g^Duke University Medical Center, Psychiatry and Behavioral Sciences, Durham, NC, USA

^h^RTI International, Behavioral Health and Criminal Justice Division, Research Triangle Park, NC, USA

^i^University of Otago Christchurch, Pathology, Christchurch, New Zealand

^j^University of Colorado Boulder, Boulder, CO, USA

^k^University of Otago Christchurch, Biostatistics and Computational Biology Unit, Christchurch, New Zealand

^l^Psychiatric Hospital Aargau, Center of addiction and Psychotherapy, Brugg, Switzerland

^m^University of Regensburg, Psychiatric hospital , Department of Psychiatry, Regensburg, Germany

^n^Washington University in Saint Louis School of Medicine, Genetics, Saint Louis, MO, USA

^o^Private Hospital Meiringen, Psychiatry, Meiringen, Switzerland

^p^Icahn School of Medicine at Mount Sinai, Neuroscience, New York, NY, USA

^q^University of California San Diego, Office of Research Affairs, La Jolla, CA, USA

^r^University of California San Diego, Psychology/Psychiatry, La Jolla, CA, USA

^s^Washington University, Psychiatry, St. Louis, MO, USA

^t^Indiana University School of Medicine, Biochemistry and Molecular Biology, Indianapolis, IN, USA

^v^Indiana University School of Medicine, Medical and Molecular Genetics, Indianapolis, IN, USA

^w^Yale University School of Medicine, Genetics and Neurobiology, New Haven, CT, USA

^x^US Department of Veterans Affairs Psychiatry, West Haven, CT, USA

^y^Yale University School of Medicine Psychiatry, New Haven, CT, USA

^z^University of Connecticut School of Medicine, Psychiatry, Farmington, CT, USA

^a/a^University of Colorado Denver, Psychiatry, Aurora, CO, USA

^a/b^University of Pennsylvania Perelman School of Medicine, Psychiatry, Philadelphia, PA, USA

^a/c^The University of Queensland, School of Psychology, Brisbane, Australia

^a/d^QIMR Berghofer Medical Research Institute, Genetics and Computational Biology, Brisbane, Australia

^a/e^University of Queensland, Institute for Molecular Biology, Brisbane, Australia

^a/f^Broad Institute, Program in Medical and Population Genetics, Cambridge, MA, USA

^a/g^Broad Institute, Stanley Center for Psychiatric Research, Cambridge, MA, USA

^a/h^Massachusetts General Hospital, Analytic and Translational Genetics Unit, Boston, MA, USA

^a/i^University of Bonn, Institute of Human Genetics, Bonn, Germany

^a/j^University of Bonn, Life&Brain Center, Department of Genomics, Bonn, Germany

^a/k^University of Colorado Boulder, Dept. of Psychology and Inst. for Behavioral Genetics, Boulder, CO, USA

^a/l^University of California San Diego, La Jolla, CA, USA

^a/m^San Diego State University & University of California, San Diego Joint Doctoral Program in Clinical Psychology , Department of Psychology, San Diego, CA, USA

^a/n^University of California San Diego, School of Medicine, Department of Psychiatry, La Jolla, CA, USA

^a/o^International Aids Vaccine Initiative Neutralizing Antibody Center at The Scripps Research Institute, Department of Molecular and Cellular Neuroscience, La Jolla, CA, USA

^a/p^Veterans Medical Research Foundation, San Diego, CA, USA

^a/q^Veterans Affairs San Diego Healthcare System, Alcohol and Drug Treatment Program, La jolla, CA, USA

^a/r^Analytic and Translational Genetics Unit (ATGU), Department of Medicine, Massachusetts General Hospital and Harvard Medical School, Boston, Massachusetts, USA

^a/s^ Stanley Center for Psychiatric Research, Broad Institute of Harvard and MIT, Cambridge, Massachusetts, USA

**ANOREXIA NERVOSA (AN)**

Cynthia M. Bulik ^a,b,c^, Gerome Breen^d,e^, Roger A Adan^f,g^, Lars Alfredsson, Tetsuya Ando, Ole A. Andreassen^i,j,^, Harald Aschauer^k^, Jessica H. Baker^l^, Vkadimir Bencko, Andrew W Bergen^m^, Wade Berrettini, Andreas Birgegård, Harry Brandt, Roland Burghardt^n^, Laura Carlberg^o^, Matteo Cassina^p^, Carolyn Cesta, Sven Cichon^q,r,s,t^, Maurizio Clementi^p^, Sarah Cohen-Woods, Jonathan R I Coleman^e,u^, Roger Cone, Philippe Courtet^a/a/h^, Steven Crawford, Scott Crow^v^, James J Crowley^w,x^, Unna Danner^y,z^, Oliver S P Davis^a/a^, George V Dedoussis^a/b^, Daniela Degortes^a/c^, Janiece E DeSocio^a/d,a/e^, Danielle M Dick^a/f^, Dimitris Dikeos^a/g^, Christian Dina, Bo Ding^a/h^, Monika Dmitrzak-Weglarz, Elisa Docampo, Laramie E Duncan^a/I,a/j,a/k^, Karin Maria Egberts^a/l^, Stefan Ehrlich^a/m^, Georgia Escaramis^a/n^, Tõnu Esko^a/o,a/p,a/q,a/r^, Thomas Espeseth, Xavier Estivill^a/s,a/t^, Angela Favaro^a/u^, Fernando Fernandez-Aranda^a/v,a/w,a/x^, Manfred Fichter^a/y^, Krista Fischer, James Floyd, Manuel Föcker^a/z^, Lenka Foretova, Monica Forzan^a/a/a^, Christopher S Franklin, Giovanni Gambaro, Héléna A Gaspar^u^, Ina Giegling^a/a/b^, Fragiskos Gonidakis, Philip Gorwood^a/a/c,a/a/d,a/a/e^, Mònica Gratacòs^a/a/f^, Sébastien Guillaume^a/a/g,a/a/h,a/a/i^, Yiran Guo, Hakon Hakonarson, Katherine A Halmi^a/a/j^, Konstantinos Hatzikotoulas^a/a/k^, Joanna Hauser^a/a/l^, Johannes Hebebrand^a/z^, Sietske G Helder^a/a/m^, Judith Hendriks, Stefan Herms^t,a/a/n,a/a/o^, Beate Herpertz-Dahlmann^a/a/p^, Wolfgang Herzog^a/a/q^, Christopher E Hilliard^a/a/r^, Anke Hinney^a/z^, Laura Huckins^a/a/s^, James Hudson, Julia Huemer, Hartmut Imgart^a/a/t^, Hidetoshi Inoko, Vladimir Janout, Susana Jimenez-Murcia^a/a/u^, Craig L Johnson^a/a/v^, Jennifer Jordan, Antonio Julià Cano^a/a/w^, Anders Juréus^a^, Gursharan Kalsi, Deborah Kaminská^a/a/x^, Allan Kaplan^a/a/y^, Jaakko Kaprioa^a/a/z,a/b/a,a/b/b,a/b/c^, Leila J Karhunen^a/b/d^, Andreas Karwautz^a/b/e^, Martien J Kas^g,a/b/f^, Walter Kaye, James L Kennedy^a/b/g,a/b/h,a/b/i,a/b/j^, Martin A Kennedy^a/b/k^, Anna Keski-Rahkonen^a/b/c^, Kirsty Kiezebrink^a/b/l^, Youl-RiKim, Lars Klareskog^a/b/m,a/b/n,a/b/o,a/b/p^, Kelly Klump^a/b/q^, Gun Peggy S Knudsen^a/b/r^, Bobby Koeleman, Doris Koubek, Mikael Landén^a,a/b/s^, Robert Levitan^a/b/I,a/b/t^, Dong Li^a/b/u^, Paul Lichtenstein^a,a/b/v^, Lisa Lilenfeld^a/b/w^, Jolanta Lissowska^a/b/x^, Astri J. Lundervold^a/b/y,a/b/z^, Pierre Julius Magistretti^a/c/a^, Mario Maj, Sara Marsal, Nicholas G Martin^a/c/b,a/c/c^, Morten Mattingsdal^a/c/d^, Sara McDevitt, Andres Metspalu^a/o,a/c/e^, Ingrid Meulenbelt, Nadia Micali^a/c/f^, James Mitchell, Karen Mitchell^a/c/g,a/c/h^, Palmiero Monteleone^a/c/i^, Alessio Maria Monteleone^a/c/j^, Grant W Montgomery^a/c/k^, Preben Bo Mortensen^a/c/l^, Melissa A Munn-Chernoff^a/c/m^, Benedetta Nacmias, Ida AK Nilsson^a/c/n,a/c/o^, Claes Norring^a/c/p,a/c/q^, Ioanna Ntalla, Julie O'Toole^a/c/r^, Roel Ophoff, Aarno Palotie, Jacques Pantel^a/c/s^, HanaPapeÅ¾ovÃ¡^a/c/t^, Richard K Parker^a/c/u^, Dalila Pinto^a/c/v^, Raquel Rabionet^a/c/w,a/c/x^, Anu Raevuori^a/c/y,a/c/z^, Nicolas Ramoz^a/d/a^, William Rayner^a/d/b^, Ted Reichborn-Kjennerud^a/d/c,a/d/d^, Valdo Ricca, Samuli Ripatti, Franziska Ritschel^a/m,a/d/e^, Marion E Roberts^a/d/f^, Dan Rujescu^a/a/b^, Filip Rybakowski^a/d/g^, Paolo Santonastaso^a/u^, André Scherag, Stephen Scherer^a/d/h,a/d/I,a/d/j^, Ulrike Schmidt^a/d/k^, Nicholas J. Schork, Alexandra Schosser^a/d/l,a/d/m^, Jochen Seitz^a/d/n^, Pieternella Slagboom^a/d/o^, Agnieszka Slopien, Tosha W Smith^c^, Nicole Soranzo^a/d/p,a/d/q,a/d/r,a/d/s^, Sandro Sorbi, Loz Southam^a/a/k,a/d/s^, Vidar M Steen^a/d/t,a/d/u^, Eric Strengman, Michael Strober^a/d/v^, Patrick F Sullivan^a,c,a/d/w^, Jin P Szatkiewicz^a/d/w^, Neonila Szeszenia-Dabrowska, Ioanna Tachmazidou, Elena Tenconi^a/d/x^, Laura M. Thornton^c^, Alfonso Tortorella, Federica Tozzi^a/d/y,a/d/z,a/e/a^, Janet Treasure^a/d/k^, Konstantinos M Tziouvas^a/e/b^, Tracey Wade, Gudrun Wagner^a/e/c^, Esther Walton, Hunna J Watson^a/c/m,a/e/d,a/e/e^, H.-Erich Wichmann, Elisabeth Widén^a/e/f^, Jack Yanovski^a/e/g^, Shuyang Yao^a^, Zeynep Yilmaz^c^, Eleftheria Zeggini^a/e/h^, Stephanie Zerwas^c^, Stephan Zipfel

^a^ Karolinska Institutet, Department of Medical Epidemiology and Biostatistics, Stockholm, Sweden

^b^ University of North Carolina at Chapel Hill, Nutrition, Chapel Hill, NC, USA

^c^ University of North Carolina at Chapel Hill, Psychiatry, Chapel Hill, NC, USA

^d^ King's College London, MRC SGDP Centre, London, UK

^e^ King's College London, NIHR BRC for Mental Health, London, UK

^f^ Altrecht Eating Disorders Rintveld, Zeist, The Netherlands

^g^ University Medical Center Utrecht, Department of Translational Neuroscience, Utrecht, The Netherlands

^h^ Karolinska Institutet, Institute of Environmental Medicine, Stockholm, Sweden

^i^Oslo University Hospital, Div Mental Health and Addiction, Oslo, Norway

^j^ University of Oslo, NORMENT, Oslo, Norway

^k^ BioPsyC - Biopsychosocial Corporation, Vienna, Austria

^l^ University of North Carolina Psychiatry, Chapel Hill, NC, USA

^m^ Biorealm, Culver City, CA, USA

^n^ Klinikum Frankfurt Oder GmbH, Child and Adolescent Psychiatry, Frankfurt Oder, Brandenburg, Germany

^o^ Medical University of Vienna, Psychiatry and Psychotherapy, Vienna, Austria

^p^ University of Padova, Clinical Genetics Unit; Department of Womenâ€™s and Childrenâ€™s Health, Padova, Italy

^q^ University of Basel, Department of Biomedicine, Basel, Switzerland

^r^ University of Basel, Division of Medical Genetics, Basel, Switzerland

^s^ Research Center Juelich, Institute of Neuroscience and Medicine (INM-1, Juelich, Germany

^t^ University of Bonn, Institute of Human Genetics, Bonn, Germany

^u^ King's College London, MRC Social, Genetic and Developmental Psychiatry Centre, London, UK

^v^ University of Minnesota, psychiatry, Minneapolis, MN, USA

^w^ Karolinska Institutet, Department of Clinical Neuroscience, Stockholm, Sweden

^x^ University of North Carolina at Chapel Hill, Departments of Genetics and Psychiatry, Chapel Hill, NC, USA

^y^ Universiteit Utrecht, Clinical Psychology, Utrecht, The Netherlands

^z^ Altrecht, Altrecht Eating Disorders Rintveld, Zeist, The Netherlands

^a/a^ University of Bristol, MRC Integrative Epidemiology Unit, Bristol, UK

^a/b^ Harokopio University Nutrition and Dietetics, Athens, Greece

^a/c^ Azienda Ospedaliera di Padova, Department of Neurosciences, Padova, Italy

^a/d^ Seattle University, College of Nursing, Seattle, WA, USA

^a/e^ Kartini Clinic, Portland, Oregon, USA

^a/f^ Virginia Commonwealth University, Department of Psychology, Department of African American Studies, Department of Human and Molecular Genetics, College Behavioral and Emotional Health Institute (director), Richmond, VA, USA

^a/g^ National and Kapodistrian University of Athens - Faculty of Medicine, 1st Department of Psychiatry, Athens, Greece

^a/h^ University of California San Diego, Chemistry and Biochemistry, La Jolla, CA, USA

^a/I^ Stanford University, Psychiatry, Stanford, CA, USA

^a/j^ Broad Institute, Medical and Population Genetics, Cambridge, MA, USA

^a/k^ Broad Institute, Stanley Center, Cambridge, MA, USA

^a/l^ Julius-Maximilians-Universität Würzburg Medizinische Fakultät, Child and Adolescent Psychiatry, Psychosomatics and Psychotherapy, Würzburg, Germany

^a/m^ TU Dresden, Faculty of Medicine, University Hospital C.G. Carus, Division of Psychological and Social Medicine and Developmental Neurosciences, Dresden, Germany

^a/n^ Centre for Genomic Regulation, CRG, Bioinformatics & Genomics, Barcelona, Spain

^a/o^ University of Tartu, Estonian Genome Center, Tartu, Estonia

^a/p^ Broad Institute, Program in Medical and Population Genetics, Cambridge, MA, USA

^a/q^ Children's Hospital Boston, Division of Endocrinology, Boston, MA, USA

^a/r^ Harvard Medical School, Department of Genetics, Boston, MA, USA

^a/s^ Sidra Medical and Research Center, Research Department, Experimental Genetics Division, Doha, Qatar

^a/t^ University Hospital Quirón Dexeus, Women's Health Department, Barcelona, Spain

^a/u^ Università degli Studi di Padova, Department of Neurosciences, Padova, Italy

^a/v^ University of Barcelona, School of Medicine, Barcelona, Spain

^a/w^ CIBEROBN, Barcelona, Spain

^a/x^ University Hospital of Bellvitge, Psychiatry, Barcelona, Spain

^a/y^ Schön Klinik Center, Prien, Germany

^a/z^ University Hospital Essen, Department of Child and Adolescent Psychiatry, Psychosomatics and Psychotherapy, Essen, Germany

^a/a/a^ University Hospital of Padova, Clinical Genetics Unit, Padova, Italy

^a/a/b^ Martin-Luther-University Halle-Wittenberg, Dept. of Psychiatry, Psychotherapy and Psychosomatics, Halle, Germany

^a/a/c^ Sainte-Anne hospital,75014 Paris, France

^a/a/d^ INSERM U894, 75014 Paris, France

^a/a/e^ Université Paris Descartes, Medical university, Paris, France

^a/a/f^ Mònica Gratacòs, Barcelona, Spain

^a/a/g^ Université de Montpellier, Faculté de Médecine, Montpellier, France

^a/a/h^ Centre Hospitalier Régional Universitaire de Montpellier,Department of Emergency Psychiatry and Post Acute Care, Montpellier, France

^a/a/I^ Inserm U1061 , Montpellier, France

^a/a/j^ Joan and Sanford I Weill Medical College of Cornell University, New York, NY, USA

^a/a/k^ Wellcome Trust Sanger Institute, Human Genetics, Cambridge, UK

^a/a/l^ Poznan University of Medical Sciences, Psychiatric Genetics Unit, Department of Psychiatry, Poznan, Poland

^a/a/m^ Zorg op Orde, Leidschendam, The Netherlands

^a/a/n^ University of Basel, Human Genomics Research Group, Department of Biomedicine, Basel, Switzerland

^a/a/o^ University of Bonn, Life&Brain Center, Department of Genomics, Bonn, Germany

^a/a/p^ University Clinics RWTH Aachen, Department of Child & Adolescent Psychiatry, D-52074 Aachen, Germany

^a/a/q^ UniversitätsKlinikum Heidelberg, Center of Psychosocial Medicine, Heidelberg, Germany

^a/a/r^ University of North Carolina, Lineberger Comprehensive Cancer Center, NC, USA

^a/a/s^ Icahn School of Medicine at Mount Sinai, Psychiatric Genomics, New York, NY, USA

^a/a/t^ Parkland-Klinik, Bad Wildungen, Germany

^a/a/u^ University Hospital of Bellvitge, Psychiatry, Barcelona, Spain

^a/a/v^ Eating Recovery Center, Psychiatry, Denver, Colorado, USA

^a/a/w^ Vall d'Hebron Institut de Recerca, Malalties sistèmiques, Barcelona, Spain

^a/a/x^ University hospital Motol, Departmen of Spiritual Care, Prague, Czech Republic

^a/a/y^ University of Toronto Faculty of Medicine, Psychiatry; Center for Addiction and Mental Health Toronto, Canada

^a/a/z^ University of Helsinki, Institute for Molecular Medicine FIMM, Helsinki, Finland

^a/b/a^ National Institute for Health and Welfare,Department of Health, Helsinki, Finland

^a/b/b^ University of Helsinki, Department of Public Health, Helsinki, Finland

^a/b/c^ University of Helsinki, Finland

^a/b/d^ University of Eastern Finland, Institute of Public Health and Clinical Nutrition, Kuopio, Finland

^a/b/e^ medical university of Vienna, c & a psychiatry, Vienna, Austria

^a/b/f^ Rijksuniversiteit Groningen, Groningen Institute for Evolutionary Life Sciences, Groningen, The Netherlands

^a/b/g^ Centre for Addiction and Mental Health, Campbell Family Mental Health Research Institute, Toronto, Canada

^a/b/h^ Centre for Addiction and Mental Health, Neurogenetics Section, Toronto, Canada

^a/b/I^ University of Toronto, Department of Psychiatry, Toronto , Canada

^a/b/j^ University of Toronto, Institute of Medical Sciences, Toronto, Canada

^a/b/k^ University of Otago Christchurch, Pathology, Christchurch, New Zealand

^a/b/l^ University of Aberdeen, Institue of Applied Health Sciences, Aberdeen, UK

^a/b/m^ Karolinska Institutet, Rheumatology, Stockholm, Sweden

^a/b/n^ Karolinska Institutet, Center for Molecular Medicine, Stockholm, Sweden

^a/b/o^ Karolinska Institutet, Department of Medicine, Solna, Stockholm, Sweden

^a/b/p^ Karolinska University Hospital, Rheumatology, Stockholm, Sweden

^a/b/q^ Michigan State University, Psychology, East Lansing, Michigan, USA

^a/b/r^ Norwegian Institute of Public Health, Health Data and Digitalisation, Oslo, Norway

^a/b/s^ University of Gothenburg, Institute of Neuroscience and Physiology, Gothenburg, Sweden

^a/b/t^ Centre for Addiction and Mental Health, Toronto, Canada

^a/b/u^ Children's Hospital of Philadelphia, Center for Applied Genomics, PA, PA, USA

^a/b/v^ Karolinska Institutet, Stockholm, Sweden

^a/b/w^ American School of Professional Psychology at Argosy University Washington DC, Clinical Psychology, Arlington, VA, USA

^a/b/x^ M. Sklodowska-Curie Cancer Center and Institute of Oncology, Cancer Epidemiology and Prevention, Warsaw, Poland

^a/b/y^ K. G. Jebsen Center for Neuropsychiatric Disorders, University of Bergen, Bergen, Norway

^a/b/z^ University of Bergen, Department of Biological and Medical Psychology, Bergen, Norway

^a/c/a^ King Abdullah University of Science and Technology (KAUST), Bioscience, Thuwal, Saudi Arabia

^a/c/b^ The University of Queensland, School of Psychology, Brisbane, Australia

^a/c/c^ QIMR Berghofer Medical Research Institute, Genetics and Computational Biology, Brisbane, Australia

^a/c/d^ University of Agder, Department of Natural Sciences, Kristiansand, Norway

^a/c/e^ University of Tartu, Institute of Molecular and Cell Biology, Tartu, Estonia

^a/c/f^ University College London, institute of child health, Population policy and practice, London, UK

^a/c/g^ Boston University, Psychiatry, Boston, MA, USA

^a/c/h^ VA Boston Health Care System, Women's Health Sciences Division, National Center for PTSD, Boston, MA, USA

^a/c/I^ University of Salerno, Department of Medicine and Surgery, Salerno, Italy

^a/c/j^ Second University of Naples , Department of Psychiatry, Naples, Italy

^a/c/k^ University of Queensland, Institute for Molecular Biology, Brisbane, Australia

^a/c/l^ Aarhus Universitet, National Centre for Register-based Research, Aarhus, Denmark

^a/c/m^ University of North Carolina at Chapel Hill, Department of Psychiatry, Chapel Hill, NC, USA

^a/c/n^ Karolinska Institutet, Department of Molecular Medicine & Surgery, Stockholm, Sweden

^a/c/o^ Center for Molecular Medicine, Stockholm, Sweden

^a/c/p^ Karolinska Institutet, Department of Clinical Neuroscience (Psychiatry), Stockholm, Sweden

^a/c/q^ Stockholms läns sjukvårdsområde, Stockholm Center for Eating Disorders, Stockholm, Sweden

^a/c/r^ Kartini Clinic, Pediatric Eating Disorders, Portland, Oregon, USA

^a/c/s^ INSERM, Toxicology, Pharmacology and Cell signaling (INSERM UMRS 1124), Paris, France

^a/c/t^ First Faculty of Medicine Charles University, Department of Psychiatry, Prague, Czech Republic

^a/c/u^ QIMR Berghofer Medical Research Institute, Genetic Epidemiology Laboratory, Herston, Australia

^a/c/v^ Icahn School of Medicine at Mount Sinai, Psychiatry, and Genetics and Genomic Sciences, New York, NY, USA

^a/c/w^ Universitat de Barcelona, Departament de Genètica, Barcelona, Spain

^a/c/x^ Centre de Regulació Genòmica, Genomics and Disease, Bioinformatics and Genomics, Barcelona, Spain

^a/c/y^ Helsinki University Central Hospital, Department of Adolescent Psychiatry, Helsinki, Finland

^a/c/z^ University of Helsinki, Clinicum, Public Health, Helsinki, Finland

^a/d/a^ INSERM, INSERM U894 Center of Psychiatry and Neuroscience, Paris, France

^a/d/b^ University of Oxford, Diabetes Research Laboratories; RDM OCDEM, Oxford, UK

^a/d/c^ University of Oslo, Institute of Clinical Medicine, Oslo, Norway

^a/d/d^ Norwegian Institute of Public Health, Department of Adult Mental Health, Oslo, Norway

^a/d/e^ Technische Universität Dresden, Eating Disorders Research and Treatment Center, Department of Child and Adolescent Psychiatry, Faculty of Medicine, Dresden, Germany

^a/d/f^ Institute of Psychiatry Psychology and Neuroscience, Department of Psychological Medicine, Section of Eating Disorders, London, UK

^a/d/g^ Poznan University of Medical Sciences, Adult Psychiatry, Poznan, Poland

^a/d/h^ The Hospital for Sick Children, Genetics and Genome Biology, Toronto, Canada

^a/d/I^ University of Toronto, McLaughlin Centre, Toronto, Canada

^a/d/j^ University of Toronto, Molecular Genetics and Institute of Medical Science, Toronto, Canada

^a/d/k^ King's College London, Psychological Medicine, London, UK

^a/d/l^ BBRZ Med, Zentrum für Seelische Gesundheit Leopoldau, Vienna, Austria

^a/d/m^ University Hospital of Psychiatry and Psychotherapeutic Medicine, Department of Biological Psychiatry, Vienna, Austria

^a/d/n^ University Hospital RWTH Aachen, Department of Child and Adolescent Psychiatry, Psychotherapy and Psychosomatics, Aachen, Germany

^a/d/o^ Leiden University Medical Center, Medical Statistics and Bioinformatics, Section Molecular Epidemiology, Leiden, The Netherlands

^a/d/p^ National Institute for Health Research Blood and Transplant Unit, Donor Health and Genomics , Cambridge, UK

^a/d/q^ British Heart Foundation Centre of Excellence, Division of Cardiovascular Medicine, Cambridge, UK

^a/d/r^ University of Cambridge, Department of Haematology, Cambridge, UK

^a/d/s^ Wellcome Trust Centre for Human Genetics, Oxford, UK

^a/d/t^ Haukeland University Hospital, Center for Medical Genetics and Molecular Medicine, Bergen, Norway

^a/d/u^ University of Bergen, Department of Clinical Science, Bergen, Norway

^a/d/v^ University of California Los Angeles, David Geffen School of Medicine, Los Angeles, CA, USA

^a/d/w^ University of North Carolina at Chapel Hill, Genetics, Chapel Hill, NC, USA

^a/d/x^ University of Padova, Neurosciences, Padova, Italy

^a/d/y^ Stremble, Brain Research, Germasogeia, Cyprus

^a/d/z^ University of Cyprus, eHealth Lab - Computer Science Department, Nicosia, Cyprus

^a/e/a^ Aptuit Srl, Verona, Italy

^a/e/b^ Aglaia Kiriakou Children's Hospital, PICU, Athens, Greece

^a/e/c^ Medical University Vienna, Dep. of Child and Adolescent Psychiatry, Vienna, Austria

^a/e/d^ Curtin University, School of Psychology and Speech Pathology, Perth, Australia

^a/e/e^ The University of Western Australia, School of Paediatrics and Child Health, Perth, Australia

^a/e/f^ Institute for Molecular Medicine Finland, Helsinki, Finland

^a/e/g^ National Institute of Child Health and Human Development, Program in Developmental Endocrinology and Genetics, Rockville, MD, USA

^a/e/h^ Wellcome Trust Sanger Institute, Human Genetics, Hinxton, UK

**ATTENTION DEFICIT/HYPERACTIVITY DISORDER (ADHD)**

Anders D. Børglum^a,b,c^, Ditte Demontis^a,b,c^, Manuel Mattheisen^a,b,c^, Jakob Grove^a,b,c,d^, Thomas D. Als^a,b,c^, Mads Engel Hauberg^a,b,c^, Jonatan Pallesen^a,b,c^, Thomas Werge^a,g,h*^, Preben Bo Mortensen^a,b,i,j^, Carsten Bøcker Pedersen^a,i,j^, Esben Agerbo^a,i,j^, Marianne Giørtz Pedersen^a,i,j^, Ole Mors^a,f^, Merete Nordentoft^a,k^, David M. Hougaard^a,e^, Mads V. Hollegaard^a,e^, Marie Bækved-Hansen^a,e^, Christine S. Hansen^a,e,g^, Jonas Bybjerg-Grauholm^a,e^, Jesper Buchave Poulsen^a,e^

^a^The Lundbeck Foundation Initiative for Integrative Psychiatric Research, iPSYCH, Denmark

^b^Centre for Integrative Sequencing, iSEQ, Aarhus University, Aarhus, Denmark

^c^Department of Biomedicine - Human Genetics, Aarhus University, Aarhus, Denmark

^d^Bioinformatics Research Centre, Aarhus University, Aarhus, Denmark

^e^Center for Neonatal Screening, Department for Congenital Disorders, Statens Serum Institut, Copenhagen, Denmark

^f^Psychosis Research Unit, Aarhus University Hospital, Risskov, Denmark

^g^Institute of Biological Psychiatry, MHC Sct. Hans, Mental Health Services Copenhagen, Roskilde, Denmark

^h^Department of Clinical Medicine, University of Copenhagen, Copenhagen, Denmark

^i^National Centre for Register-Based Research, Aarhus University, Aarhus, Denmark

^j^Centre for Integrated Register-based Research, Aarhus University, Aarhus, Denmark

^k^Mental Health Services in the Capital Region of Denmark, Mental Health Center Copenhagen, University of Copenhagen, Copenhagen, Denmark

*TW has acted as advisor and lecturer to H. Lundbeck A/S

**AUTISM SPECTRUM DISORDER (ASD)**

AD Børglum^a,b,c^, J Grove^a,b,c,d^, M Mattheisen^a,b,c^, TD Als^a,b,c^, D Demontis^a,b,c^, J Pallesen^a,b,c^, ME Hauberg^a,b,c^, M Nyegaard^a,b,c^, T Werge^a,g,h*^, PB Mortensen^a,b,i,j^, CB Pedersen^a,i,j^, E Agerbo^a,i,j^, M Giørtz-Pedersen^a,i,j^, O Mors^a,f^, M Nordentoft^a,k^, DM Hougaard^a,e^, MV Hollegaard^a,e^, M Bækved-Hansen^a,e^, CS Hansen^a,e,g^, J Bybjerg-Grauholm^a,e^, J Buchhave-Poulsen^a,e^ J Martin^l,q,r^, AR Martin^l,m,n^, J Maller^l^, J Moran^l^, S Ripke^l,m,n,p^, A Dumont^l^, JI Goldstein^l,m,n^, FK Satterstrom^l,m,n^, DS Palmer^l,m,n^, F Cerrato^l^, DP Howrigan^l,m,n^, E Robinson^l,o^, C Stevens^l^, T Poterba^l,m,n^, R Walters^l,m,n^, M Daly^l,m,n^, B Neale^l,m,n*^

^*^TW has acted as advisor and lecturer to H. Lundbeck A/S

^*^BN is on the Scientific Advisory Board for Deep Genomics

^a^The Lundbeck Foundation Initiative for Integrative Psychiatric Research, iPSYCH, Denmark

^b^Centre for Integrative Sequencing, iSEQ, Aarhus University, Aarhus, Denmark

^c^Department of Biomedicine - Human Genetics, Aarhus University, Aarhus, Denmark

^d^Bioinformatics Research Centre, Aarhus University, Aarhus, Denmark

^e^Center for Neonatal Screening, Department for Congenital Disorders, Statens Serum Institut, Copenhagen, Denmark

^f^Psychosis Research Unit, Aarhus University Hospital, Risskov, Denmark

^g^Institute of Biological Psychiatry, MHC Sct. Hans, Mental Health Services Copenhagen, Roskilde, Denmark

^h^Department of Clinical Medicine, University of Copenhagen, Copenhagen, Denmark

^i^National Centre for Register-Based Research, Aarhus University, Aarhus, Denmark

^j^Centre for Integrated Register-based Research, Aarhus University, Aarhus, Denmark

^k^Mental Health Services in the Capital Region of Denmark, Mental Health Center Copenhagen, University of Copenhagen, Copenhagen, Denmark

^l^Analytic and Translational Genetics Unit (ATGU), Department of Medicine, Massachusetts General Hospital and Harvard Medical School, Boston, Massachusetts, USA

^m^ Program in Medical and Population Genetics, Broad Institute of Harvard and MIT, Cambridge, Massachusetts, USA

^n^ Stanley Center for Psychiatric Research, Broad Institute of Harvard and MIT, Cambridge, Massachusetts, USA

^o^Department of Epidemiology, Harvard Chan School of Public Health, Boston, Massachusetts, USA.

^p^Department of Psychiatry, Charite Universitatsmedizin Berlin Campus Benjamin Franklin, Berlin, Germany.

^q^MRC Centre for Neuropsychiatric Genetics and Genomics: Cardiff, Cardiff, United Kingdom.

^r^Department of Medical Epidemiology and Biostatistics, Karolinska Institutet, Stockholm, Sweden.

**BIPOLAR DISORDER (BIP)**

Annelie Nordin Adolfsson^a/a/x^, Ingrid Agartz^a,b,c^, Huda Akil^d^, Martin Alda^e,f^, Ney Alliey-Rodriguez^g^, Ole A. Andreassen^b,h^, Adebayo Anjorin^i^, Jack D Barchas^j^, Nicholas James Bass, Michael Bauer^k^, Bernhard T Baune^l^, Sarah E. Bergen^m^, Andrew Bethell, Joanna M Biernacka^n^, Michael Boehnke^o^, Marco Boks, E Bruno, William Bunney^q^, Margit Burmeister^r^, Pablo Cervantes, Sven Cichon^s,t,u,v^, Nick Craddock^w^, Cristiana Cruceanu^x^, David Curtis^y,z^, Piotr M. Czerski^a/a^, Anders Dale^a/b^, Franziska Degenhardt^v, a/c^, A Devin, Jurgen Del Favero, Arianna Di Florio, Srdjan Djurovic^a/d, a/b/u^, Torbjørn Elvsåshagen^a/e,a/f^, Chun Chieh Fan, A Farmer, M Ferreira, IN Ferrier, Sascha B Fischer^a/g^, Matthew Flickinger^o^, Andreas J Forstner^v,a/c,a/g,a/h^, Liz Forty, Josef Frank^a/j^, Christine Fraser, Nelson Freimer, Mark Andrew Frye,^a/k^ Janice M Fullerton, Elliot Gershon^g^, Scott D Gordon^a/l^, Katherine Gordon-Smith^a/m^, Elaine K Green^a/n^, D Grozeva, H Gurling, M Hipolito, LM Huckins, S Jamain, TA Greenwood, Maria Grigoroiu-Serbanescu^a/o^, Weihua Guan^a/p^, Jose Guzman-Parra^a/q^, Marian Hamshere, J Kammerer-Ciernioch, Joanna Hauser^a/a^, Martin Hautzinger^a/r^, Stefan Herms^v, a/c, a/g^, Per Hoffmann^v,a/c,a/g,a/s^, Peter Holmans, Christina Hultman^m^, Ian Jones, Lisa A Jones^a/m^, Anders Juréus^m^, Radhika Kandaswamy, Robert Karlsson^m^, James L Kennedy^a/t,a/u,a/v,a/w^, JR Kelsoe, D Koller, George Kirov, Sarah Kittel-Schneider^a/x^, Sarah V Knott^a/m^, Manolis Kogevinas, Ralph Kupka^a/y,a/z,a/a/a^, N Langstrom, M Lathrop, WB Lawson, Mikael Landén^m,a/a/b^, Jacob Lawrence^a/a/c^, Markus Leber^a/a/d^, Shawn E. Levy^a/a/e^, Jun Z. Li^a/a/f^, P Lichtenstein, FW Lohoff, A Maasser, PB Mahon, W Maier, M Mattheisen, K Matthews, M Mattingsdal, S Meier, V Milanova, J Moran, D Morris, V Moskvina, Jolanta Lissowska^a/a/g^, Chunyu Liu^g^, Susanne Lucae^a/a/h^, Anna Maaser^v, a/c^, Ulrik Malt^a/a/j^, Nicholas G Martin^a/l,a/a/k^, Fermin Mayoral-Cleries^a/q^, Susan L McElroy^a/a/l^, K McGhee, P McGuffin, MG McInnis, A McIntosh, AW McLean, FJ, McMahon, James D McKay^a/a/m^, Andrew McQuillin^a/a/n^, Sarah Elizabeth Medland^a/a/o^, Ingrid Melle^h,a/a/p^, Fan Meng^a/a/q^, Philip Mitchell, Grant W Montgomery^a/a/r^, Gunnar Morken^a/a/s,a/a/t^, Thomas W Mühleisen^u,a/a/u^, Bertram Müller Myhsok^x,a/a/v,a/a/w^, Richard M. Myers^a/a/e^, Markus M Nöthen^v,a/c^, CM Nievergelt, I Nikolov, V Nimgaonkar, EA Nwulia, U Osby, John Nurnberger^a/b/w^, Michael C O'Donovan^a/a/y^, Ketil Joachim Oedegaard^a/a/z,a/b/a^, Loes M Olde Loohuis^a/b/b^, Roel Ophoff, Anil Ori^a/b/b^, Lilijana Oruc^a/b/c^, Michael J Owen^a/a/y^, JG Para, C Pato, R Perlis, Sara A Paciga^a/b/d^, Amy Perry^a/m^, Andrea Pfennig^k^, BS Pickard, Amy Perry, JB Potash, S Purcell, E Quinn, S Raychaudhuri, Eline J Regeer^a/b/e^, Andreas Reif^a/x^, Céline S Reinbold^a/g^, J Rice, Marcella Rietschel^a/j^, Fabio Rivas^a/q^, Guy A Rouleau^a/b/f,a/b/g^, D Ruderfer, WA Scheftner, NJ Schork, J Schumacher, M Schwarz, E Scolnick, PD Shilling, P Sklar, EN Smith, D St Clair, E Stahl, M Steffens, PF Sullivan, S Szellinger, N Szeszenia-Dabrowska, Alan F Schatzberg, Peter Schofield, Thomas G Schulze^a/j,a/b/h,a/b/I,a/b/j,a/b/k^, LJ Scott^o^, Olav B Smeland^b,h^, Jordan W Smoller^a/b/l,a/b/m,a/b/n^, Anne T. Spijker^a/b/o^, Eystein Stordal^a/b/p,a/b/q^, John Strauss, Fabian Streit^a/j^, Jana Strohmaier^a/j^, Robert C Thompson^a/b/r^, Jens Treutlein^a/j^, Gustavo Turecki^a/b/s^, Arne Vaaler^a/a/t^, H Vedder, John Vincent, Yunpeng Wang^a/b/t,a/b/u^, Stanley J Watson^a/b/r^, R Williamson, A Winslow, A Wright, Stephanie H Witt^a/j^, Hualin S Xi^a/b/v^, Wei Xu, AH Young, PP Zandi, P Zhang, S Zöllner, Howard J. Edenberg^a/b/x, a/b/y^

^a^Diakonhjemmet Hospital, Department of Psychiatric Research, Oslo, Norway

^b^University of Oslo, NORMENT, KG Jebsen Centre for Psychosis Research, Division of Mental Health and Addiction, Institute of Clinical Medicine, Oslo, Norway

^c^Karolinska Institutet , Department of Clinical Neuroscience, Centre for Psychiatric Research, Stockholm , Sweden

^d^University of Michigan, Molecular & Behavioral Neuroscience Institute, Ann Arbor, MI, USA

^e^National Institute of Mental Health , Klecany, Czech Republic

^f^Dalhousie University Department of Psychiatry, Halifax, Canada

^g^University of Chicago, Department of Psychiatry and Behavioral Neuroscience, Chicago, IL, USA

^h^Oslo University Hospital, Div Mental Health and Addiction, Oslo , Norway

^i^Berkshire Healthcare NHS Foundation Trust, Psychiatry, Bracknell, UK

^j^Weill Cornell Medical College, Department of Psychiatry, NY, NY, USA

^k^University Hospital Carl Gustav Carus, Department of Psychiatry and Psychotherapy, Dresden, Germany

^l^University of Adelaide Discipline of Psychiatry, Adelaide, Australia

^m^Karolinska Institutet, Department of Medical Epidemiology and Biostatistics, Stockholm, Sweden

^n^Mayo Clinic, Health Sciences Research, Rochester, USA

^o^University of Michigan, Center for Statistical Genetics and Department of Biostatistics, Ann Arbor, MI, USA

^p^UMC Utrecht Hersencentrum Rudolf Magnus, Psychiatry, Utrecht, The Netherlands

^q^University of California, Irvine, Department of Psychiatry and Human Behavior, Irvine, CA, USA

^r^University of Michigan, Molecular & Behavioral Neuroscience Institute and Department of Computational Medicine & Bioinformatics, Ann Arbor, MI, USA

^s^University of Basel, Department of Biomedicine, Basel, Switzerland

^t^University of Basel, Division of Medical Genetics, Basel, Switzerland

^u^Research Center Juelich, Institute of Neuroscience and Medicine (INM-1), Juelich, Germany

^v^University of Bonn, Institute of Human Genetics, Bonn, Germany

^w^Cardiff University, Psychological Medicine, Cardiff, UK

^x^Max Planck Institute of Psychiatry, Department of Translational Research in Psychiatry, Munich, Germany

^y^Queen Mary University of London, Centre for Psychiatry, London, UK

^z^University College London, UCL Genetics Institute, London, UK

^a/a^Poznan University of Medical Sciences, Department of Psychiatry, Laboratory of Psychiatric Genetics, Poznan , Poland

^a/b^University of California San Diego, Neurosciences, Radiology, Psychiatry, Cognitive Science, La Jolla, CA, USA

^a/c^University of Bonn, Life&Brain Center, Department of Genomics, Bonn, Germany

^a/d^University of Bergen, NORMENT, KG Jebsen Centre for Psychosis Research, Department of Clinical Science, Bergen, Norway

^a/e^Oslo Universitetssykehus, NORMENT, KG Jebsen Centre for Psychosis Research, Oslo, Norway

^a/f^Oslo Universitetssykehus, Department of Neurology, Oslo, Norway

^a/g^University of Basel, Human Genomics Research Group, Department of Biomedicine, Basel, Switzerland

^a/h^University of Basel, Department of Psychiatry (UPK), Basel, Switzerland

^a/i^Cardiff University, School of Medicine, Cardiff, UK

^a/j^Central Institute of Mental Health, Medical Faculty Mannheim, Heidelberg University, Department of Genetic Epidemiology in Psychiatry, Mannheim, Germany

^a/k^Mayo Clinic, Department of Psychiatry & Psychology, Rochester, MN, USA

^a/l^QIMR Berghofer Medical Research Institute, Genetics and Computational Biology, Brisbane, Australia

^a/m^University of Worcester, Department of Psychological Medicine, Worcester, UK

^a/n^Plymouth University Peninsula Schools of Medicine and Dentistry, School of Biomedical and Healthcare Sciences, Plymouth, UK

^a/o^Alexandru Obregia Clinical Psychiatric Hospital , Biometric Psychiatric Genetics Research Unit, Bucharest, Romania

^a/p^University of Minnesota, System Biostatistics, Minneapolis, MN, USA

^a/q^University Regional Hospital. Biomedicine Institute (IBIMA), Mental Health Department, Malaga, Spain

^a/r^Eberhard Karls Universitaet Tuebingen, Psychology, Tuebingen, Germany

^a/s^Universityhospital Basel, Institute for Medical Genetics and Pathology, Basel, Switzerland

^a/t^Centre for Addiction and Mental Health, Campbell Family Mental Health Research Institute, Toronto, Canada

^a/u^Centre for Addiction and Mental Health, Neurogenetics Section, Toronto, Canada

^a/v^University of Toronto, Department of Psychiatry, Toronto, Canada

^a/w^University of Toronto, Institute of Medical Sciences, Toronto, Canada

^a/x^University Hospital of Frankfurt , Department of Pychiatry, Psychosomatic Medicine and Psychotherapy, Frankfurt/Main, Germany

^a/y^GGZ ingest, Psychiatry, Amsterdam, The Netherlands

^a/z^VU medisch centrum, Psychiatry, Amsterdam, The Netherlands

^a/a/a^Altrecht, Psychiatry, Utrecht, The Netherlands

^a/a/b^University of Gothenburg,Institute of Neuroscience and Physiology, Gothenburg, Sweden

^a/a/c^North East London NHS Foundation Trust, Psychiatry, Ilford, UK

^a/a/d^University Hospital Cologne, Clinic for Psychiatry and Psychotherapy, Cologne, Germany

^a/a/e^HudsonAlpha Institute for Biotechnology, Huntsville, USA

^a/a/f^University of Michigan, Department of Human Genetics, Ann Arbor, MI, USA

^a/a/g^M. Sklodowska-Curie Cancer Center and Institute of Oncology, Cancer Epidemiology and Prevention, Warsaw, Poland

^a/a/h^Max Planck Institute of Psychiatry, Munich, Germany

^a/a/i^Life&Brain, Genomics, Bonn, Germany

^a/a/j^Oslo Universitetssykehus, Research and Education, Division of Clinical Neuroscience, Oslo, Norway

^a/a/k^The University of Queensland, School of Psychology, Brisbane, Australia

^a/a/l^Lindner Center of HOPE, Research Institute, Mason, USA

^a/a/m^International Agency for Research on Cancer, Genetic Cancer Susceptibility Group, Lyon, France

^a/a/n^University College London, Division of Psychiatry, London, UK

^a/a/o^QIMR Berghofer Medical Research Institute, Genetics and Computational Biology, Herston, Australia

^a/a/p^University of Oslo, Institute of Clinical Medicine, Division of Mental Health and Addiction, Oslo, Norway

^a/a/q^University of Michigan, Psychiatry and Molecular and Behavior Neuroscience Institute, Ann Arbor, MI, USA

^a/a/r^University of Queensland, Institute for Molecular Biology, Brisbane, Australia

^a/a/s^Norwegian University of Science and Technology - NTNU Mental Health, Faculty of Medicine and Health Sciences, Trondheim, Norway

^a/a/t^St Olavs University Hospital, Psychiatry, Trondheim, Norway

^a/a/u^University of Basel, Division of Medical Genetics, Department of Biomedicine, Basel, Switzerland

^a/a/v^Munich Cluster for Systems Neurology (SyNergy), Munich, Germany

^a/a/w^University of Liverpool, Liverpool, UK

^a/a/x^ Umeå Universitet Medicinska fakulteten, Department of Clinical Sciences, Psychiatry, Umea, Sweden

^a/a/y^Cardiff University School of Medicine, MRC Centre for Neuropsychiatric Genetics and Genomics, Cardiff, UK

^a/a/z^Haukeland Universitetssjukehus, Division of Psychiatry, Bergen, Norway

^a/b/a^University of Bergen, Faculty of Medicine and Dentistry, Bergen, Norway

^a/b/b^University of California Los Angeles, Center for Neurobehavioral Genetics, Los Angeles, CA, USA

^a/b/c^Psychiatry Clinic, Clinical Center University of Sarajevo, Sarajevo, Bosnia and Herzegovina

^a/b/d^Pfizer Global Research and Development, Human Genetics and Computational Biomedicine, Groton, CT, USA

^a/b/e^Altrecht, Outpatient Clinic for Bipolar Disoder, Utrecht, The Netherlands

^a/b/f^McGill University, Faculty of Medicine, Department of Neurology and Neurosurgery, Montreal, Canada

^a/b/g^Montreal Neurological Institute and Hospital, Montreal, Canada

^a/b/h^Medical Center of the University of Munich, Campus Innenstadt, Institute of Psychiatric Phenomics and Genomics (IPPG), Munich, Germany

^a/b/i^University Medical Center Goettingen, Department of Psychiatry and Psychotherapy, Goettingen, Germany

^a/b/j^Johns Hopkins University, Department of Psychiatry and Behavioral Sciences, Baltimore, MD, USA

^a/b/k^NIMH Division of Intramural Research Programs, Human Genetics Branch, Bethesda, MD, USA

^a/b/l^Massachusetts General Hospital, Department of Psychiatry, Boston, MA, USA

^a/b/m^Massachusetts General Hospital, Psychiatric and Neurodevelopmental Genetics Unit (PNGU), Boston, MA, USA

^a/b/n^Broad Institute, Stanley Center for Psychiatric Research, Cambridge, MA, USA

^a/b/o^PsyQ, Mood Disorders, Rotterdam, The Netherlands

^a/b/p^Norges Teknisk Naturvitenskapelige Universitet, Fakultet for naturvitenskap og teknologi, Department of neuroscience, Trondheim, Norway

^a/b/q^Hospital Namsos, Department of psychiatry, Namsos, Norway

^a/b/r^University of Michigan, Department of Psychiatry, Ann Arbor, MI, USA

^a/b/s^McGill University, Department of Psychiatry, Montreal, Canada

^a/b/t^Mental Health Centre Sct. Hans, Institute of Biological Psychiatry, Copenhagen, Denmark

^a/b/u^University of Oslo, Institute of Clinical Medicine, Oslo, Norway

^a/b/v^Pfizer Global Research and Development, Computational Sciences Center of Emphasis, Cambridge, MA, USA

^a/b/w^Indiana University School of Medicine, Psychiatry, Indianapolis, IN, USA

^a/b/x^Indiana University School of Medicine, Biochemistry and Molecular Biology, Indianapolis, IN, USA

^a/b/y^Indiana University School of Medicine, Medical and Molecular Genetics, Indianapolis, IN, USA

**MAJOR DEPRESSIVE DISORDER (MDD)**

Patrick F Sullivan^a,b,c^, Stephan Ripke^d,e,f^, Danielle Posthuma^g,h^, Henning Tiemeier^i,j,k^, André G Uitterlinden^l^, Nese Direk^j^, Saira Saeed Mirza^j^, Albert Hofman^j^, Susanne Lucae^m^, Stefan Kloiber^m^, Klaus Berger^n^, Jürgen Wellmann^n^, Bertram Müller-Myhsok^o,p,q^, Qingqin S Li^r^, Marcus Ising^m^, Till F M Andlauer^m^**^,o^**, Stefan Kloiber^m^, Marcella Rietschel^s^, Andreas J Forstner^t,u, u1,u2,u3^, Fabian Streit^s^, Jana Strohmaier^s^, Wolfgang Maier^s^, Josef Frank^s^, Stefan Herms^t,u,v^, Stephanie H Witt^s^, Jens Treutlein^s^, Markus M Nöthen^t,u^, Sven Cichon^t,w,x,y^, Franziska Degenhardt^t,u^, Per Hoffmann^t,u,z^, Thomas G Schulze^s,a/a,a/b,a/c,a/d^, Bernhard T Baune^a/e^, Udo Dannlowski^a/f,a/g^, Tracy M Air^a/e^, Grant C B Sinnamon^a/h^, Naomi R Wray^a/i^, Andrew M McIntosh^a/j^, Douglas H R Blackwood^a/j^, Toni-Kim Clarke^a/j^, Donald J MacIntyre^a/j^, David J Porteous^a/k^, Caroline Hayward^a/l^, Tõnu Esko^e,a/m,a/n,a/o^, Evelin Mihailov^a/m,a/p^, Lili Milani^a/m^, Andres Metspalu^a/m, a/q^, Hans J Grabe^a/r^, Henry Völzke^a/s^, Alexander Teumer^a/s^, Sandra Van der Auwera^a/r^, Georg Homuth^a/t^, Matthias Nauck^a/u^, Cathryn M Lewis^a/v,a/w^, Gerome Breen^a/w,a/x^, Margarita Rivera^a/w,a/y^, Michael Gill^a/z^, Nick Craddock^a/a/a^, John P Rice^a/a/b^, Michael J Owen^a/a/c^, Henriette N Buttenschøn^a/a/d^, Ole Mors^a/a/e^, Anders D Børglum^a/a/f,a/a/g,a/a/h^, Jakob Grove^a/a/f,a/a/g,a/a/h,a/a/i^, Jesper Krogh^a/a/j^, Enrico Domenici^a/a/k^, Daniel Umbricht^a/a/k^, Jorge A Quiroz^a/a/k^, Carsten Horn^a/a/l^, Enda M Byrne^a/i^, Baptiste Couvy-Duchesne^a/a/m,a/a/n,a/a/o^, Scott D Gordon^a/a/m^, Andrew C Heath^a/a/b^, Anjali K Henders^a/i^, IB Hickie^a/a/p^, Pamela AF Madden^a/a/b^, Nicholas G Martin^a/a/m,a/a/o^, Sarah E Medland^a/a/m^, Grant W Montgomery^a/a/q^, Dale R Nyholt^a/a/r^, Michele L Pergadia^a/a/s^, Divya Mehta^a/i^, Martin Preisig^a/a/t^, Enrique Castelao^a/a/t^, Zoltán Kutalik^a/a/u,a/a/v^, Steven P Hamilton^a/a/w^, Katherine E Tansey^a/a/x^, Rudolf Uher^a/a/y^, Glyn Lewis^a/a/z^, Michael C O'Donovan^a/a/c^, Brenda WJH Penninx^a/b/a^, Yuri Milaneschi^a/b/a^, Wouter J Peyrot^a/b/a^, Johannes H Smit^a/b/a^, Rick Jansen^a/b/a^, Aartjan TF Beekman^a/b/a^, Robert Schoevers^a/b/b^, Albert M van Hemert^a/b/c^, Gerard van Grootheest^a/b/a^, Dorret I Boomsma^a/b/d^, Jouke- Jan Hottenga^a/b/d^, Christel M Middeldorp^a/b/d, a/b/d1, a/b/d2^, EJC de Geus^a/b/d,a/b/e^, Abdel Abdellaoui^a/b/d^, Gonneke Willemsen^a/b/d^, Erin C Dunn^a/b/f,a/b/g,a/b/h^, Roy H Perlis^a/b/g,a/b/i^, Jordan W Smoller^a/b/f,a/b/g,a/b/h^, Patrik K Magnusson^a^, Nancy L Pedersen^a^, Alexander Viktorin^a^, Erik Pettersson^a^, Thomas Werge^a/b/j,a/b/k,a/b/l^, Thomas F Hansen^a/b/j,a/b/l,a/b/m^, Sara A Paciga^a/b/n^, Hualin S Xi^a/b/o^, Ashley R Winslow^a/b/n^, Douglas F Levinson^a/b/q^, Myrna M Weissman^a/b/r,a/b/s^, James B Potash^a/b/t^, Jianxin Shi^a/b/u^, James A Knowles^a/b/v^, Peter McGuffin^a/w^

^a^Karolinska Institutet, Medical Epidemiology and Biostatistics, Stockholm,Sweden

^b^University of North Carolina at Chapel Hill, Genetics, Chapel Hill, NC, USA

^c^University of North Carolina at Chapel Hill, Psychiatry, Chapel Hill, NC, USA

^d^Charite Universitatsmedizin Berlin Campus Benjamin Franklin, Department of Psychiatry, Berlin, Germany

^e^Broad Institute, Medical and Population Genetics, Cambridge, MA, USA

^f^Massachusetts General Hospital, Analytic and Translational Genetics Unit, Boston, MA, USA

^g^VU medical center, Clinical Genetics, Amsterdam, The Netherlands

^h^VU University Amsterdam, Complex Trait Genetics, Amsterdam, The Netherlands

^i^Erasmus MC, Psychiatry, Rotterdam, The Netherlands

^j^Erasmus MC, Epidemiology, Rotterdam, The Netherlands

^k^Erasmus MC, Child and Adolescent Psychiatry, Rotterdam, The Netherlands

^l^Erasmus MC, Internal Medicine, Rotterdam, The Netherlands

^m^Max Planck Institute of Psychiatry, Munich, Germany

^n^University of Muenster, Institute of Epidemiology and Social Medicine, Muenster, Germany

^o^Munich Cluster for Systems Neurology (SyNergy), Munich, Germany

^p^University of Liverpool, Liverpool, UK

^q^Max Planck Institute of Psychiatry, Department of Translational Research in Psychiatry, Munich, Germany

^r^Janssen Research and Development, LLC, Neuroscience Therapeutic Area, Titusville, NJ, USA

^s^Department of Psychiatry and Psychotherapy, University of Bonn, Bonn, Germany

^t^Institute of Human Genetics, University of Bonn, Bonn, Germany

^u^ Department of Genomics, Life&Brain Center, University of Bonn, Bonn, Germany

^u1^Department of Psychiatry (UPK), University of Basel, Basel, Switzerland

^u2^ Human Genomics Research Group, Department of Biomedicine, University of Basel, Basel, Switzerland

^u3^ Institute of Medical Genetics and Pathology, University Hospital Basel, Basel, Switzerland

^v^University of Basel, Division of Medical Genetics and Department of Biomedicine, Basel, Switzerland

^w^University of Basel, Department of Biomedicine, Basel, Switzerland

^x^University of Basel, Division of Medical Genetics, Basel, Switzerland

^y^Research Center Juelich, Institute of Neuroscience and Medicine (INM-1), Juelich, Germany

^z^University of Basel, Human Genomics Research Group, Department of Biomedicine, Basel, Switzerland

^a/a^Medical Center of the University of Munich, Campus Innenstadt, Institute of Psychiatric Phenomics and Genomics (IPPG), Munich, Germany

^a/b^University Medical Center Göttingen, Department of Psychiatry and Psychotherapy, Goettingen, Germany

^a/c^Johns Hopkins University, Department of Psychiatry and Behavioral Sciences, Baltimore, MD, USA

^a/d^NIMH Division of Intramural Research Programs, Human Genetics Branch, Bethesda, MD, USA

^a/e^University of Adelaide, Discipline of Psychiatry, Adelaide, Australia

^a/f^ University of Münster, Germany

^a/g^University of Münster, Department of Psychiatry, Münster, Germany

^a/h^James Cook University, School of Medicine and Dentistry, Townsville, Australia

^a/i^The University of Queensland, Queensland Brain Institute, Brisbane, Australia

^a/j^University of Edinburgh, Division of Psychiatry, Edinburgh, UK

^a/k^University of Edinburgh, Medical Genetics Section, CGEM, IGMM, Edinburgh, UK

^a/l^University of Edinburgh, Medical Research Council Human Genetics Unit, Institute of Genetics and Molecular Medicine, Edinburgh, UK

^a/m^University of Tartu, Estonian Genome Center, Tartu, Estonia

^a/n^Children's Hospital Boston, Division of Endocrinology, Boston, MA, USA

^a/o^Harvard Medical School, Department of Genetics, Boston, MA, USA

^a/p^Estonian Biocentre, Tartu, Estonia

^a/q^University of Tartu, Institute of Molecular and Cell Biology, Tartu, Estonia

^a/r^University Medicine Greifswald, Department of Psychiatry and Psychotherapy, Greifswald, Germany

^a/s^University Medicine Greifswald, Institute for Community Medicine, Greifswald, Germany

^a/t^University Medicine and Ernst Moritz Arndt University Greifswald, Interfaculty Institute for Genetics and Functional Genomics, Department of Functional Genomics, Greifswald, Germany

^a/u^University Medicine Greifswald, Institute of Clinical Chemistry and Laboratory Medicine,Greifswald, Germany

^a/v^King's College London, Department of Medical & Molecular Genetics, London, UK

^a/w^King's College London, MRC Social Genetic and Developmental Psychiatry Centre, London, UK

^a/x^King's College London, NIHR BRC for Mental Health, London, UK

^a/y^University of Granada, Instituto de Investigación Biosanitaria ibs.Granada and CIBER en Salud Mental (CIBERSAM), Granada, Spain

^a/z^Trinity College Dublin, Department of Psychiatry, Dublin, Ireland

^a/a/a^Cardiff University, Psychological Medicine, Cardiff, UK

^a/a/b^Washington University in Saint Louis School of Medicine, Department of Psychiatry, Saint Louis, MO, USA

^a/a/c^Cardiff University School of Medicine, MRC Centre for Neuropsychiatric Genetics and Genomics, Cardiff, UK

^a/a/d^Aarhus University, Department of Clinical Medicine, Translational Neuropsychiatry Unit, Aarhus, Denmark

^a/a/e^Aarhus University Hospital, Risskov, Research Department P, Aarhus, Denmark

^a/a/f^Aarhus University, iSEQ, Centre for Integrative Sequencing, Aarhus, Denmark

^a/a/g^iPSYCH, The Lundbeck Foundation Initiative for Integrative Psychiatric Research, Aarhus , Denmark

^a/a/h^Aarhus University, Department of Biomedicine, Aarhus, Denmark

^a/a/i^Aarhus University, Bioinformatics Research Centre (BiRC), Aarhus, Denmark

^a/a/j^University of Copenhagen, Deparment of Endocrinology at Herlev University Hospital, Copenhagen, Denmark

^a/a/k^F. Hoffmann-La Roche Ltd, Roche Pharmaceutical Research and Early Development, Neuroscience, Ophthalmology and Rare Diseases Discovery & Translational Medicine Area, Roche Innovation Center, Basel, Switzerland

^a/a/l^F. Hoffmann-La Roche Ltd, Roche Pharmaceutical Research and Early Development, Pharmaceutical Sciences, Roche Innovation Center, Basel, Switzerland

^a/a/m^QIMR Berghofer Medical Research Institute, Genetics and Computational Biology, Herston, Australia

^a/a/n^The University of Queensland, Centre for Advanced Imaging, Saint Lucia, Australia

^a/a/o^The University of Queensland, School of Psychology, Saint Lucia, Australia

^a/a/p^University of Sydney, Brain and Mind Research Institute, Sydney, Australia

^a/a/q^University of Queensland, Institute for Molecular Biology, Brisbane, Australia

^a/a/r^Queensland University of Technology, Institute of Health and Biomedical Innovation, Brisbane, Australia

^a/a/s^Florida Atlantic University, Charles E. Schmidt College of Medicine, Boca Raton, FL, USA

^a/a/t^University Hospital of Lausanne, Department of Psychiatry, Prilly, Switzerland

^a/a/u^University Hospital of Lausanne, Institute of Social and Preventive Medicine (IUMSP), Lausanne, Switzerland

^a/a/v^Swiss Institute of Bioinformatics, Lausanne, Switzerland

^a/a/w^Kaiser Permanente Northern California, Psychiatry, San Francisco, CA, USA

^a/a/x^University of Bristol, MRC Integrative Epidemiology Unit, Bristol, UK

^a/a/y^Dalhousie University, Psychiatry, Halifax, Canada

^a/a/z^University College London, Division of Psychiatry, London, UK

^a/b/a^VU University Medical Center and GGZ ingeest, Department of Psychiatry, Amsterdam, The Netherlands

^a/b/b^University of Groningen, University Medical Center Groningen, Department of Psychiatry, The Netherlands

^a/b/c^Leiden University Medical Center, Department of Psychiatry, Leiden, The Netherlands

^a/b/d^VU University Amsterdam, Dept of Biological Psychology, Amsterdam, The Netherlands

^a/b/d1^Child Health Research Centre, University of Queensland, Australia

^a/b/d2^Child and Youth Mental Health Service, Children’s Health Queensland Hospital and Health Service, Australia

^a/b/e^VU University Medical Center, EMGO+ Institute, Amsterdam, The Netherlands

^a/b/f^Broad Institute, Stanley Center for Psychiatric Research, Cambridge, MA, USA

^a/b/g^Massachusetts General Hospital, Department of Psychiatry, Boston, MA, USA

^a/b/h^Massachusetts General Hospital, Psychiatric and Neurodevelopmental Genetics Unit (PNGU), Boston, MA, USA

^a/b/i^Harvard Medical School, Psychiatry, Boston, MA, USA

^a/b/j^The Lundbeck Foundation Initiative for Psychiatric Research, iPSYCH, Copenhagen, Denmark

^a/b/k^University of Copenhagen, Institute of Clinical Medicine, Copenhagen, Denmark

^a/b/l^Mental Health Services Capital Region of Denmark, Institute of Biological Psychiatry, Mental Health Center Sct. Hans, Copenhagen, Denmark

^a/b/m^Department of Neurology, Copenhagen University Hospital, Headache Centre, Glostrup, Denmark

^a/b/n^Pfizer Global Research and Development, Human Genetics and Computational Biomedicine, Groton, CT, USA

^a/b/o^Pfizer Global Research and Development, Computational Sciences Center of Emphasis, Cambridge, MA, USA

^a/b/p^Pfizer Global Research and Development, Human Genetics and Computational Medicine, Cambridge, MA, USA

^a/b/q^Stanford University, Psychiatry & Behavioral Sciences, Stanford, CA, USA

^a/b/r^New York State Psychiatric Institute, Division of Epidemiology, New York, NY, USA

^a/b/s^Columbia University College of Physicians and Surgeons, Psychiatry, New York , NY, USA

^a/b/t^University of Iowa, Psychiatry, Iowa City, IA, USA

^a/b/u^National Cancer Institute, Division of Cancer Epidemiology and Genetics, Bethesda, MD, USA

^a/b/v^University of Southern California, Psychiatry & The Behavioral Sciences, Los Angeles, CA, USA

**OBESSIVE COMPULSIVE DISORDER (OCD)**

Kelly Anderson, Paul Arnold^a^, Kathleen Askland^b^, Cristina Barlassina^c^, Laura Bellodi^d^, O. Joseph Bienvenu^e^, Donald Black^f^, Michael Bloch^g^, Rianne Blom^h^, Helena Brentani^i^, Christie Burton^j^, Beatriz Camarena^k^, Carolina Cappi^i^, Danielle Cath^l^, Maria Cavallini^m^, David Conti^n^, Edwin Cook^o^, Vladimir Coric^p^, Bernadette Cullen^e^, Daniele Cusi^q^, Lea Davis^r^, Dieter Deforce^s^, Richard Delorme^t^, Damiaan Denys^u^, Eske Derks^h^, Valsama Eapen^v^, Christopher Edlund^n^, Karin Egberts^w^, Lauren Erdman^x^, Peter Falkai^y^, Martijn Figee^h^, Abby J Fyer^z^, Helena Garrido, Daniel Geller^a/a^, Fernando Goes^e^, Hans Grabe^a/b^, Marco Grados^e^, Benjamin Greenberg^a/c^, Wei Guo^a/d^, Edna Grünblatt^a/e^, Gregory Hanna^a/f^, Sian Hemmings^a/g^, Ana Hounie^i^ , Michael Jenike^a/h^, Clare Keenan^a/a/g^, James Kennedy^a/i^, Ekaterin Khramtsova^a/a/g^, James Knowles^a/j^, Anua Konkashbaev^a/a/g^, Cristophe Lange^a/k^, Nuria Lanzagorta^a/l^, Marion Leboyer^a/m^, Leonhard Lennertz^a/n^, Bingbin Li^x^, Kung-Yee Liang^a/o^, Christine Lochner^a/g^, Fabio Macciardi^a/a/k^, Brion Maher^e^, Wolfgang Maier^a/n^, Maurizio Marconi^a/p^, Carol A. Mathews^a/q^, Manuel Mattheisen^a/r^, James McCracken^a/u^, Nicole McLaughlin^a/c^, Euripedes Miguel^i^, Rainald Moessner^a/a/h^, Dennis Murphy^a/s^, Benjamin Neale^a/a^, Paul S Nestadt^a/a/i^, Gerald Nestadt^e^, Humberto Nicolini^a/t^, Erika Nurmi^a/u^, Lisa Osiecki^a/h^, Michele Pato^a/j^, Carlos Pato^a/j^, David Pauls^a/h^, John Piacentini^a/u^, Danielle Posthuma^a/v^, Ann Pulver^e^, Steven Rasmussen^a/c^, Scott Rauch^a/a^, Tobias Renner^w^, Margaret Richter^a/x^, Mark Riddle^e^, Stephan Ripke^a/a/j^, Maria Rosário^a/y^, David Rosenberg^a/z^, Stephan Ruhrmann^a/a/a^, Aline Sampaio^i^, Jack Samuels^e^, Jeremiah Scharf^a/a^, Yin Yao Shugart^a/d^, Jan Smit^a/a/b^, Dan Stein^a/g^, S Evelyn Stewart, Maurizio Turiel^d^, Homero Vallada^i^, Filip Van Nieuwerburgh^s^, Jeremy Veenstra-VanderWeele^a/a/d^, Nienke Vulink^h^, Michael Wagner^a/n^, Susanne Walitza^a/a/e^, Ying Wang^e^, Jens Wendland^a/a/f^, Dongmei Yu^a/a^, Gwyneth Zai^d^

^a^University of Calgary, School of Medicine, Psychiatry, Calgary, USA

^b^Butler Hospital, Psychiatry and Human Behavior, Providence, USA

^c^University of Milano, Dept Heath Sciences, Milano, Italy

^d^Università Vita-Salute San Raffaele Milano, Department of Neuropsychiatric Sciences, Milano, Italy

^e^John Hopkins University School of Medicine, Psychiatry and Behavioral Sciences, Baltimore, USA

^f^University of Iowa School of Medicine, Psychiatry, Iowa City, USA

^g^Yale University School of Medicine, Child Study Center, Psychiatry, Connecticut, USA

^h^Academic Medical Center, University of Amsterdam, Psychiatry, Amsterdam, The Netherlands

^i^University of São Paulo, Medical School, Psychiatry, Sao Paulo, Brazil

^j^Hospital for Sick Children, Genetics and Genome Biology Program, Toronto, Canada

^k^Instituto Nacional de Psiquiatría Ramón de la Fuente Muãiz, Schizophrenia Clinic, Mexico City, Mexico

^l^Utrecht University, Clinical and Health Psychology, Utrecht, The Netherlands

^m^Ospedale San Raffaele Milano, Clinical Neuroscience, Milano, Italy

^n^University of Southern California Keck School of Medicine, Department of Preventative Medicine, Division of Biostatistic, Los Angeles, USA

^o^Institute for Juvenile Research, University of Illinois Chicago School of Medicine, Psychiatry, Chicago, USA

^p^Yale University, Psychiatry, New Haven, USA

^q^Italian National Institute of Research, Institute of Biomedical Technologies, Milan, Italy

^r^Vanderbilt Genetics Institute, Department of Medicine, Division of Genetic Medicine, Vanderbilt University Medical Center, Nashville, USA

^s^University of Ghent, Laboratory of Pharmaceutical Biotechnology, Ghent, Belgium

^t^AP-HP, Robert Debré Hospital, Child and Adolescent Psychiatry, Paris, France

^u^Academic Medical Center and Netherlands Institute for Neuroscience, Institute of the Royal Netherlands Academy of Arts and Sciences (NIN-KNAW), Psychiatry, Amsterdam, The Netherlands

^v^University of New South Wales, Psychiatry, Sydney, Australia

^w^University Hospital of Würzburg, Child and Adolescent Psychiatry, Psychosomatics and Psychotherapy, Würzburg, Germany

^x^University of Toronto, Psychiatry, Toronto, Canada

^y^LMU, Psychiatry and Psychotherapy, Munich, Germany

^z^Columbia University Medical Center, Psychiatry, New York, USA

^a/a^Massachusetts General Hospital, Harvard Medical School, Psychiatry, Boston, USA

^a/b^Helios-Hospital Stralsund, University Medicine Greifswald, Psychiatry and Psychotherapy, Griefswald, Germany

^a/c^Alpert Medical School of Brown University, Psychiatry and Human Behavior, Butler Hospital, Providence, USA

^a/d^National Institute of Mental Health, Unit on Statistical Genomics, Bethesda, USA

^a/e^University of Zurich, Child and Adolescent Psychiatry, Zurich, Switzerland

^a/f^University of Michigan School of Medicine, Psychiatry, Ann Arbor, USA

^a/g^University of Stellenbosch, Psychiatry, MRC Unit on Anxiety and Stress Disorders, Cape Town, South Africa

^a/h^Harvard, Hospital, Medical School, Psychiatry, Boston, USA

^a/i^University of Toronto, Centre for Addiction and Mental Health, Toronto, Canada

^a/j^University of Southern California School of Medicine, Psychiatry and Behavioral Sciences, Los Angeles, USA

^a/k^Harvard T.H Chan School of Public Health, Biostatistics, Harvard, USA

^a/l^Universidad Autónoma de la Ciudad de México, Center for Genomic Sciences, Mexico City, Mexico

^a/m^Hôpitaux universitaires Henri-Mondor, Pôle de Psychiatrie, Créteil, France

^a/n^University of Bonn, Psychiatry and Psychotherapy, Bonn, Germany

^a/o^The Johns Hopkins Bloomberg School of Public Health, Biostatistics, Baltimore, USA

^a/p^Milano Cord Blood Bank, Blood Transfusion Center • Foundation IRCCS Ca' Granda • Ospedale Maggiore Policlinico, Milano, Italy

^a/q^University of Florida School of Medicine College of Medicine, Psychiatry, Gainseville, USA

^a/r^Aarhus University, Biomedicine, Aarhus, Denmark

^a/s^National Institute of Mental Health, Laboratory of Clinical Science, Bethesda, USA

^a/t^Universidad Autónoma de la Ciudad de México (UACM), Psychiatry, Mexico City, Mexico

^a/u^UCLA Semel Institute for Neuroscience and Human Behavior, Psychiatry and Biobehavioral Sciences, Los Angeles, USA

^a/v^Vrije Universiteit Amsterdam, Department of Complex Trait Genetics, Center for Neurogenomics and Cognitive Research, Amsterdam, The Netherlands

^a/w^University of Würzburg, Child and Adolescent Psychiatry, Psychosomatics and Psychotherapy, Würzburg, Germany

^a/x^Sunnybrook Health Sciences Centre, Psychiatry, Sunnybrook Health Sciences Centre,Toronto, Canada

^a/y^Universidade Federal de Sao Paulo (UNIFESP, Federal University of São Paulo), Psychiatry, Sao Paulo, Brazil

^a/z^Wayne State University School of Medicine, Psychiatry and Behavioral Neurosciences, Detroit, USA

^a/a/a^University of Cologne, Psychiatry and Psychotherapy, Cologne, Germany

^a/a/b^VU University Medical Center, Psychiatry, Amsterdam, The Netherlands

^a/a/c^British Columbia Mental Health and Addictions Research Institute, University of British Columbia, Psychiatry, Vancouver, Canada

^a/a/d^Vanderbilt University, Psychiatry, Nashville, USA

^a/a/e^University of Zurich, Child and Adolescent Psychiatry and Psychotherapy, Zürich, Switzerland

^a/a/f^Nestle Health Science, Brain Health, Novel Therapeutic Nutrition, Cambridge, USA

^a/a/g^University of Chicago, Department of Human Genetics, Department of Medicine University of Chicago, Chicago, USA

^a/a/h^Universitaet Tuebingen, Deparment of Psychiatry and Psychotherapy, Tuebingen, Germany

^a/a/i^John Hopkins Bloomberg School of Public Health, Mental Health, Baltimore, USA
^a/a/j^Broad Institute, Harvard Medical School, Cambridge, USA
^a/a/k^University of California, Irvine, Department of Psychiatry and Human Behavior, School of Medicine Irvine, California

**SCHIZOPHRENIA (SCZ) CLOZUK**

Antonio F. Pardiñas^a^, Peter Holmans^a^, Andrew J. Pocklington^a^, Valentina Escott-Price^a^, Stephan Ripke^b,c,^ Noa Carrera^a^, Sophie E. Legge^a^, Sophie Bishop^a^, Darren Cameron^a^, Marian L. Hamshere^a^, Jun Han^a^, Leon Hubbard^a^, Amy Lynham^a^, Kiran Mantripragada^a^, Elliott Rees^a^, James H. MacCabe^d^, Steven A. McCarroll^e^, Bernhard T. Baune^f^, Gerome Breen^g,h^, Enda M. Byrne^i^, Udo Dannlowski^j,k^, Thalia C. Eley^g^, Caroline Hayward^l^, Nicholas G. Martin^m,n^, Andrew M. McIntosh^o,p^, Robert Plomin^g^, David J. Porteous^l^, Naomi R. Wray^i^, GERAD1 Consortium^q^, David A. Collier^g,r^, Dan Rujescu^s,t^, George Kirov^a^, Michael J. Owen^a^*, Michael C. O'Donovan^a^*, James T. R. Walters^a^*

^a^ MRC Centre for Neuropsychiatric Genetics and Genomics, Institute of Psychological Medicine and Clinical Neurosciences, School of Medicine, Cardiff University, Cardiff, UK

^b^ Analytic and Translational Genetics Unit, Massachusetts General Hospital, Boston, Massachusetts, USA

^c^ Department of Psychiatry and Psychotherapy, Charité, Campus Mitte, 10117 Berlin, Germany

^d^ Department of Psychosis Studies, Institute of Psychiatry Psychology and Neuroscience, King's College London

^e^ Stanley Center for Psychiatric Research, Broad Institute of MIT and Harvard, Cambridge, Massachusetts, USA

^f^ Discipline of Psychiatry, University of Adelaide, Australia

^g^ Medical Research Council, Social, Genetic and Developmental Psychiatry Centre, Institute of Psychiatry, Psychology and Neuroscience, King’s College London, London, UK

^h^ NIHR Biomedical Research Centre for Mental Health, Maudsley Hospital and Institute of Psychiatry, Psychology and Neuroscience, King's College London, London, UK

^i^ Queensland Brain Institute, University of Queensland, Brisbane, Queensland, Australia

^j^ Department of Psychiatry and Psychotherapy University of Muenster, Muenster, Germany

^k^ University of Münster, Germany

^l^ Medical Genetics Section, Centre for Genomic and Experimental Medicine, Institute of Genetics and Molecular Medicine, University of Edinburgh, Edinburgh, UK

^m^ School of Psychology, University of Queensland

^n^ QIMR Berghofer Medical Research Institute, Brisbane, Queensland, Australia

^o^ Division of Psychiatry, University of Edinburgh, Edinburgh, UK

^p^ Centre for Cognitive Ageing and Cognitive Epidemiology, University of Edinburgh, Edinburgh, UK

^q^ Control data used in the preparation of this article were obtained from the Genetic and Environmental Risk for Alzheimer’s disease (GERAD1) Consortium. As such, the investigators within the GERAD1 consortia contributed to the design and implementation of GERAD1 and/or provided control data but did not participate in analysis or writing of this report.

^r^ Discovery Neuroscience Research, Eli Lilly and Company Ltd, Lilly Research Laboratories, Erl Wood Manor, Surrey, UK

^s^ Department of Psychiatry, University of Halle, Halle, Germany

^t^ Department of Psychiatry, University of Munich, Munich, Germany
