# Supplementary material for: Genetic influences on eight psychiatric disorders based on family data of 4 408 646 full and half-siblings, and genetic data of 333 748 cases and controls
Source: Psychol Med. 2018 Sep 17;49(7):1166–73. doi: 10.1017/S0033291718002039 (PMC6421104; doi:10.1017/S0033291718002039)
Supplement: Supplementary file 1 [file S0033291718002039sup.zip › S0033291718002039sup002.docx]

Sensitivity analyses

We conducted two sets of sensitivity analyses to examine whether the pre-specified age of the second sibling might have been too young. First, we conducted the analyses in the adult population only by including pairs in which the youngest sibling was 20 years old or older. Results are displayed in Appendix 1, and were very similar for all disorders except for OCD, for which the heritability estimate decreased from .38 to .24. However, due to a lack of maternal half-sibling cases, the standard error about this estimate was 0.20, indicating a lack of precision such that the two estimates did not differ significantly.

Second, we plotted the distributions of the age at first diagnosis for each disorder. We then re-ran the analyses but only included pairs where the younger sibling was at least as old as the median age of diagnosis. This way, the youngest siblings were more likely to have lived through the risk period of onset. Results are displayed in Appendix 2, and show that the estimates are remarkably similar to the original analyses (although the standard errors are larger).

In sum, the heritability estimates remained very similar regardless of whether we relied on our original age cutoffs, or on adults or the median age of onset.
